# BAP1 is required prenatally for differentiation and maintenance of postnatal murine enteric nervous system

**DOI:** 10.1172/JCI177771

**Published:** 2024-03-12

**Authors:** Sabine Schneider, Jessica B. Anderson, Rebecca P. Bradley, Katherine Beigel, Christina M. Wright, Beth A. Maguire, Guang Yan, Deanne M. Taylor, J. William Harbour, Robert O. Heuckeroth

**Affiliations:** 1 Children’s Hospital of Philadelphia Research Institute, Abramson Research Center, Philadelphia, Pennsylvania, USA.; 2 Perelman School of Medicine at the University of Pennsylvania, Philadelphia, Pennsylvania, USA.; 3 Department of Neurology, Perelman School of Medicine at the University of Pennsylvania, Philadelphia, Pennsylvania, USA.; 4 Department of Biomedical and Health Informatics, Children’s Hospital of Philadelphia Research Institute, Abramson Research Center, Philadelphia, Pennsylvania, USA.; 5 Department of Pediatrics, Perelman School of Medicine at the University of Pennsylvania, Philadelphia, Pennsylvania, USA.; 6 Department of Ophthalmology, University of Texas Southwestern Medical Center, Dallas, Texas, USA.

**Keywords:** Development, Gastroenterology, Neurodegeneration

## Abstract

Epigenetic regulatory mechanisms are underappreciated, yet are critical for enteric nervous system (ENS) development and maintenance. We discovered that fetal loss of the epigenetic regulator *Bap1* in the ENS lineage caused severe postnatal bowel dysfunction and early death in *Tyrosinase-Cre Bap1^fl/fl^* mice. *Bap1*-depleted ENS appeared normal in neonates; however, by P15, *Bap1*-deficient enteric neurons were largely absent from the small and large intestine of *Tyrosinase-Cre Bap1^fl/fl^* mice. Bowel motility became markedly abnormal with disproportionate loss of cholinergic neurons. Single-cell RNA sequencing at P5 showed that fetal *Bap1* loss in *Tyrosinase-Cre Bap1^fl/fl^* mice markedly altered the composition and relative proportions of enteric neuron subtypes. In contrast, postnatal deletion of *Bap1* did not cause enteric neuron loss or impaired bowel motility. These findings suggest that BAP1 is critical for postnatal enteric neuron differentiation and for early enteric neuron survival, a finding that may be relevant to the recently described human BAP1-associated neurodevelopmental disorder.

## Abstract 2

BAP1 de-ubiquitinase regulates the polycomb repressor complex and its prenatal loss alters cell fate and causes progressive postnatal enteric neuron loss, mimicking chronic intestinal pseudo-obstruction.

## Graphical abstract

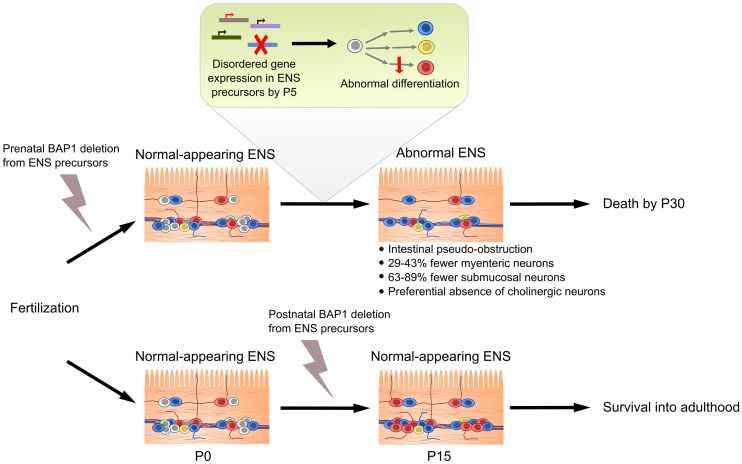

## Introduction

The enteric nervous system (ENS) rivals the spinal cord in complexity, with more than 20 neuron and at least 4 glial subtypes forming integrated circuits throughout the bowel (1, 2). These ENS cells regulate epithelial fluid flux, epithelial maintenance and repair, intestinal blood flow, and immune system activity and coordinate smooth muscle contraction and relaxation (2, 3). Congenital or acquired ENS defects (enteric neuropathies) disrupt coordinated smooth muscle activity, causing abdominal distention, constipation, vomiting, growth failure, and pain. ENS defects also impair intestinal epithelial and immune barriers, predisposing to sepsis and death (4).

The best-understood ENS defect is Hirschsprung disease (HSCR), which occurs when ENS precursors fail to fully colonize bowel during the first trimester of pregnancy. In HSCR, distal bowel completely lacks ENS at birth (4). Profound bowel dysfunction also occurs if the ENS is defective or damaged, a problem called neuropathic chronic intestinal pseudo-obstruction (nCIPO). Causes of nCIPO include abnormal ENS development, genetic changes that impair ENS function, ENS damage, and neurodegenerative diseases. More than 23 loci are linked to HSCR (4), including *RET*, *EDNRB*, and *SOX10*, but few human genetic causes of nCIPO are known (*ERBB2/3* [ref. 5], *TFAP2B* [ref. 6], *SOX10*, *FLNA*, *SGOL1*, *RAD21* [ref. 7], and mitochondrial genes *LIG3* [ref. 8], *POLG1*, and *TYMP* [ref. 7]). These loci explain only a small proportion of nCIPO cases (7).

During development, neural crest–derived ENS precursors undergo drastic changes in cell identity. These precursors arise via epithelial-mesenchymal transition from neural tube (9, 10) and generate immature enteric neurons and glia as they colonize fetal bowel. Ultimately, precursors become specialized mature neuron and glia subtypes (11). All these processes theoretically require epigenetic reorganization of chromatin landscapes to achieve coordinated regulation of cell type– and stage-specific gene expression.

Consistent with this hypothesis, mutations in murine epigenetic regulators *Aebp2* or *Ezh2* cause distal bowel aganglionosis (absence of ENS ganglia) mimicking HSCR (12, 13). AEBP2 and EZH2 are subunits of Polycomb repressor complex 2 (PRC2), an epigenetic regulator involved in many cell fate decisions (14). EZH2 (as part of PRC2) trimethylates histone 3, generating H3K27me3 (14), which recruits Polycomb repressor complex 1 (PRC1) to chromatin. PRC1 regulates gene expression by nucleosome compaction and mono-ubiquitylation of histone 2AK119 (H2AK119). H2AK119 ubiquitylation maintains and recruits PRC2 and thus H3K27me3 on chromatin, stabilizing PRC1/PRC2 (15) and restricting PRC1/PRC2 to specific chromatin regions (16). H2AK119 mono-ubiquitylation can be reversed by the BAP1-ASXL1 deubiquitylase (PR-DUB) (17, 18). Interestingly, PRC2 is regulated by retinoic acid receptors (RARs) after retinoic acid (RA) binds RAR/RXR (19). RA is derived from vitamin A. Both vitamin A deficiency (20) and expression of a dominant-negative RAR (21) in the ENS cause distal bowel aganglionosis. Moreover, RA signaling impacts enteric neuron differentiation (22). Critical ENS roles for PRC1/PRC2 are also supported by the observation that *SALL1* mutations predispose to HSCR (23). SALL1 stabilizes CBX4, a PRC1 component (24). Furthermore, reduced DNA methyltransferase 3B (DNMT3B) activity may predispose to HSCR (25), while loss of *Pcgf1* (a PRC1 subunit) alters enteric neuron subtype specification without causing aganglionosis (26). Beyond these observations, there are surprisingly few data about how epigenetics impacts the ENS, although mutations in the PRC components *EZH1*, *EZH2*, *SUZ12*, *EED2*, and H3K27 demethylases *KDM6A* and *KDM6B* all cause human central nervous system neurodevelopmental disorders (27–32).

Several questions remain unanswered. First, is PRC-associated epigenetic regulation simply required for ENS precursors to colonize fetal bowel, or does epigenetics more broadly dictate ENS cell fate specification as suggested by the *Pcgf1^–/–^* phenotype? Second, does PRC activity need to be reversed or restricted for later cell fate decisions? For example, differentiation to mature neurons and glia could require antagonism of PRC-mediated transcriptional repression.

We were thus intrigued by our serendipitous discovery that *Bap1* deletion in ENS lineages caused profound bowel dysfunction and early death in mice, mimicking human nCIPO or HSCR physiology. Based on links to *AEBP2*, *EZH2*, and RA signaling, and knowing that *BAP1* mutations reduce *SOX10* and *EDNRB* levels in uveal melanoma (33), we initially hypothesized that ENS *Bap1* loss would cause distal bowel aganglionosis, the defining feature of HSCR. However, BAP1 has many roles in addition to H2AK119ub deubiquitylase activity (18) that could impact ENS biology, including DNA replication fork elongation (34) and double-stranded DNA damage repair (35). BAP1 also represses or activates transcription by associating with or regulating other transcription factors (36). Furthermore, BAP1 stabilizes cytoplasmic type 3 inositol-1,4,5-trisphosphate receptor (IP3R3) by deubiquitylation, facilitating apoptosis in cells that accumulate DNA damage (37). Thus, it is not surprising that human *BAP1* mutations predispose to cancer (18) and global *Bap1* knockout causes early embryonic lethality in mice (38). Similarly, *Xenopus* *bap1* is required for commitment of pluripotent cells to germ layers and for development of neural crest–derived lineages (39). These observations suggest that *Bap1* could affect ENS development or function in many ways.

To test the hypothesis that conditional *Bap1* loss in ENS lineages causes HSCR-like disease, we bred floxed *Bap1* to *Wnt1-Cre* and *Tyrosinase-Cre* (*Tyr-Cre*) mice. *Wnt1-Cre^+^* express *Cre* in all fetal ENS precursors starting embryonic day 8.5 (E8.5) (40). *Tyr-Cre^+^* express *Cre* in most (>80%) ENS precursors by E10.5 (41). These Cre drivers also induce DNA recombination outside the ENS (e.g., *Wnt1-Cre* induces DNA recombination in the central nervous system and in neural crest that gives rise to bones of the face). Unexpectedly, ENS appeared to form normally before birth in *Bap1^fl/fl^* *Wnt1-Cre^+^* (called *Wnt1Bap1* KO) and *Bap1^fl/fl^ Tyr-Cre^+^* (called *TyrBap1* KO) mice. *TyrBap1* KO, however, lost most Cre lineage–marked enteric neurons between postnatal day 0 (P0) and P15. Single-cell RNA sequencing (scRNA-Seq) at P5 showed that without *Bap1*, ENS precursor differentiation was impaired. ENS defects were accompanied by massive bowel dilation, profound dysmotility, and death by P30. In contrast, *TyrBap1* KO glia were normal in abundance at P15 and had relatively minor changes in gene expression at P5. *Bap1*-mutant mice provide what we believe to be a new nCIPO model and an unusual example of rapid postnatal enteric neuron loss, highlighting critical changes in ENS that occur during this early postnatal developmental window. These findings may be relevant not only to ENS biology, but also to the recently described human syndromic neurodevelopmental disorder attributed to *BAP1* variants (42).

## Results

### Loss of Bap1 in ENS causes failure to thrive.

**Figure 1:**
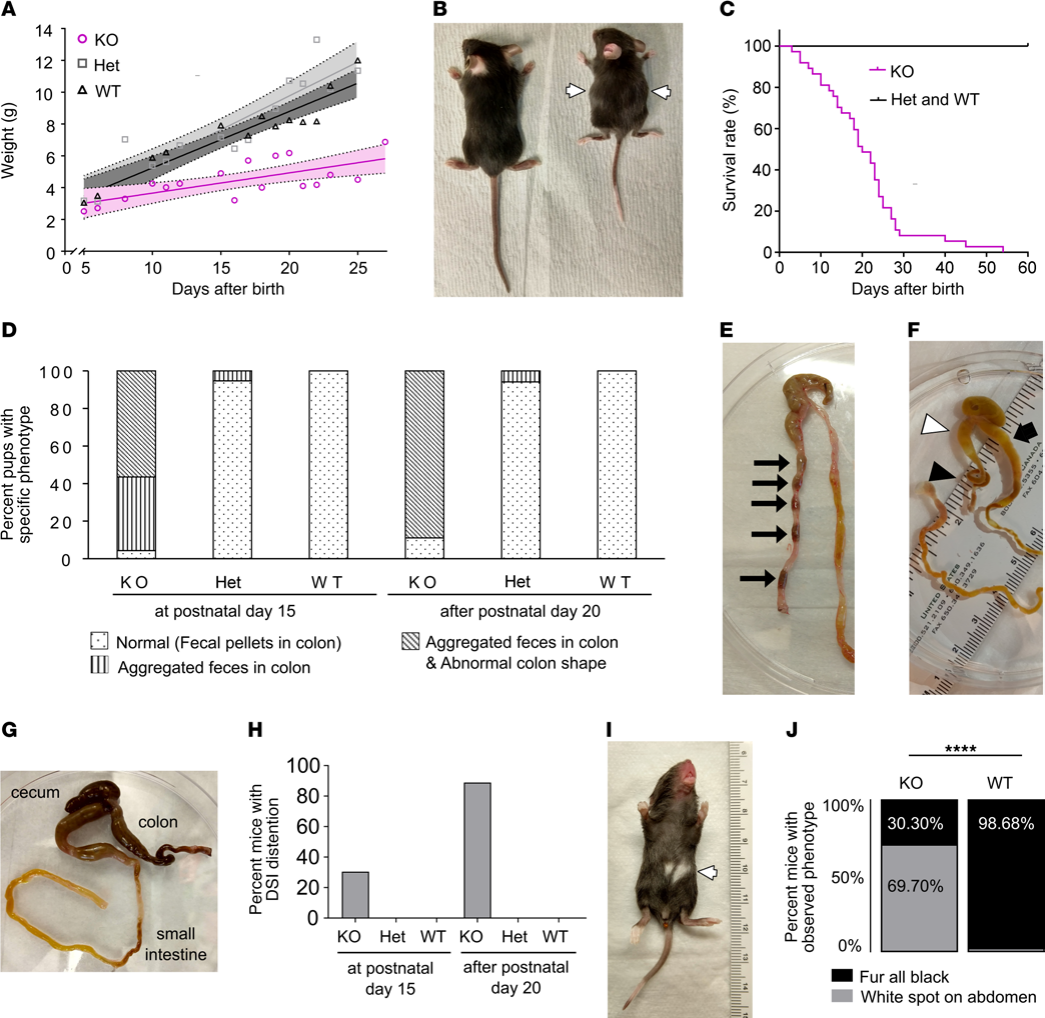
*TyrBap1^+^* mice fail to thrive and die with massively dilated bowel. (**A**) *TyrBap1* KO (KO) mice gained weight more slowly than *Bap1^fl/wt^ Tyr-Cre^+^* heterozygous (Het) and WT littermates. Points indicate mean weight. Linear regression and 95% confidence interval are shown. (**B**) *TyrBap1* KO mice (right) were smaller than WT (left) or heterozygous (not shown) littermates. A representative P20 *TyrBap1* KO mouse had visible abdominal distention (white arrows). (**C**) *TyrBap1* KO died early (median survival 20 days, *n* = 37). Only mice left with parents and soft moist food lived beyond P25. (**D**) Most *TyrBap1* KO had an abnormal colon by P15, with increased severity as age increased. (**E**) P15 WT typically had well-formed stool pellets in mid- and distal colon (arrows). (**F**) P15 *TyrBap1* KO colon was often deformed in ways never seen in controls. Black arrowhead highlights twisted and stiff mid-colon. The more proximal colon (white arrowhead) and distal small intestine (black arrow) accumulated loose feces. (**G**) *TyrBap1* KO colon and distal small intestine became severely distended owing to aggregated feces later in life. Representative P27 *TyrBap1* KO bowel. (**H**) Feces accumulated in the distal small intestine (DSI) of *TyrBap1* KO, increasing with age (30.4% at P15 and 88.9% at >P20). (**I**) White fur spots were common on *TyrBap1* KO abdomen (white arrow) indicating incomplete melanocyte colonization (P10 mouse). (**J**) Quantitative analysis of incomplete skin colonization by melanocytes. (**A**–**J**) KO refers to *Bap1^fl/fl^ Tyr-Cre^+^*, Het refers to *Bap1^fl/wt^ Tyr-Cre^+^*, and WT refers to *Bap1^wt/wt^ Tyr-Cre^+^* genotype, except in **A**, where WT includes *Bap1^wt/wt^ Tyr-Cre^+^* and mice lacking *Cre*. ****P < 0.0001. (**A**) Repeated-measures 1-way ANOVA. (**C**) Log-rank (Mantel-Cox) test. (**J**) Two-tailed binomial test.

*TyrBap1* KO could not be distinguished from WT (*Bap1^wt/wt^ Tyr-Cre^+^*) littermates at birth but grew slowly (Figure 1, A and B, and Supplemental Figure 1; supplemental material available online with this article; https://doi.org/10.1172/JCI177771DS1) and never reached WT size. Median survival was 20 days (Figure 1C). Few *TyrBap1* KO survived beyond P40 even if they remained with parents and had soft, moist food. By P15, *TyrBap1* KO colon rarely contained well-formed fecal pellets. Instead, proximal colon was distended by feces (Figure 1, D–F). Toward the end of life, *TyrBap1* KO had distended abdomens (Figure 1B) with marked stool accumulation in proximal colon and distal small intestine (Figure 1G). Distal colon typically remained normal diameter. In some *TyrBap1* KO, mid- to proximal colon became deformed (twisted and rigid) by P15 (Figure 1, F and G). Beyond P20, 8 of 9 *TyrBap1* KO had deformed colons with accumulated stool (Figure 1G). Distal small intestine stool accumulation increased with age (Figure 1H). This never occurred in heterozygous (Het) or WT mice. These *TyrBap1* KO phenotypes resemble HSCR, a neurocristopathy. Neural crest also gives rise to tyrosinase-expressing melanocytes, and most *TyrBap1* KO had white abdominal fur (Figure 1, I and J).

### Bowel motility is reduced in Bap1 conditional knockout mice.

**Figure 2:**
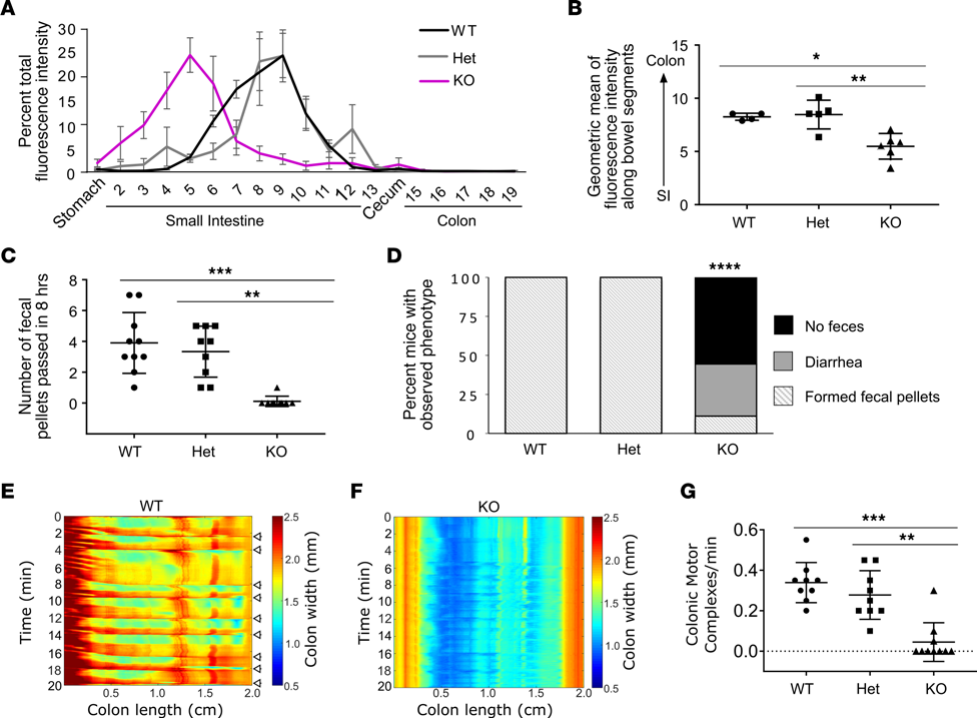
Proximal small intestine and colon motility are significantly impaired in P15 *TyrBap1* KO mice. (**A**) *TyrBap1* KO have slow transit of FITC-dextran through proximal small bowel. Mean FITC fluorescence distribution ± SEM 90 minutes after gavage. Numbered segments of bowel from stomach (segment 1) to distal rectum (segment 19) are arranged along the *x* axis. (**B**) Geometric center of FITC fluorescence was more proximal for *TyrBap1* KO than for Het and WT. Numbered bowel segments are indicated on the *y* axis. SI, small intestine. (**C**) P15 *TyrBap1* KO passed few stool pellets over 8 hours compared with Het or WT. (**D**) Most P15 *TyrBap1* KO had abnormal stool (diarrhea or no feces at all). (**E** and **F**) Representative kymographs depicting bowel width as a function of time and distance along proximal-distal axis of cannulated colon for P15 WT (**E**) and *TyrBap1* KO (**F**). (**G**) Fewer colonic motor complexes were recorded for *TyrBap1* KO as compared with Het and WT colons. (**A**–**G**) Het refers to *Bap1^fl/wt^ Tyr-Cre^+^* genotype, and KO refers to *TyrBap1* KO. (**A** and **B**) WT includes *Bap1^wt/wt^ Tyr-Cre^+^* genotype and mice lacking *Cre*. (**C**–**G**) WT refers to *Bap1^wt/wt^; Tyr-Cre^+^* genotype. (**B**, **C**, and **G**) Data are shown as mean ± SD. Data not significant unless otherwise indicated. *P < 0.05, **P < 0.01, ***P < 0.001, ****P < 0.0001. (**B**) Welch’s ANOVA test with multiple comparisons. (**C** and **E**–**G**) Kruskal-Wallis test with Dunn’s multiple comparison. (**D**) Two-sided binomial test.

To define physiology underlying fecal stasis in *TyrBap1* KO, we assessed bowel motility. We first evaluated P15 mice since 83.3% of *TyrBap1* KO were alive, yet distal bowel appeared abnormal (Figure 1, C, D, F, and H). To assess small bowel motility in vivo, we gavage-fed FITC-dextran and assessed FITC distribution 90 minutes later. FITC-dextran moved slowly through normal-appearing *TyrBap1* KO proximal small bowel, suggesting that motility defects are not limited to distal small bowel or colon (Figure 2, A and B). Consistent with reduced motility, 8 of 9 P15 *TyrBap1* KO passed no fecal pellets in 8 hours, whereas WT and Het littermates passed approximately 8 fecal pellets in the same interval. Two *TyrBap1* KO that passed stool had small-volume watery diarrhea (Figure 2, C and D). To test the hypothesis that *TyrBap1* KO have colon-intrinsic motility defects, we evaluated motility ex vivo, converting videos of full-length P15 colons to kymographs (Figure 2, E and F). All WTs and Hets had contractions that propagated from proximal to distal colon (Figure 2E) and matched the definition of colonic motor complexes (CMCs) proposed by Corsetti et al. (43). As expected, CMCs disappeared after addition of tetrodotoxin, a voltage-gated sodium channel inhibitor (Supplemental Figure 2, A and B). In contrast, most *TyrBap1* KO had no neurogenic colon contractions (5/10; Figure 2, F and G, and Supplemental Figure 2, C and F) or had abnormal retrograde contractions (3/10) that were very slow or involved only part of colon (Supplemental Figure 2, E and F). A single *TyrBap1* KO colon had both abnormal contractions and one normal-appearing CMC. Two P15 *TyrBap1* KO had exclusively normal neurogenic motility (Supplemental Figure 2F). Consistent with this observation, some P15 *TyrBap1* KO looked healthy (Supplemental Figure 1), and a small percentage passed stool but had fewer neurogenic CMCs in vitro than WTs or Hets (Figure 2G). Motility defects were not due to tonic neuron-induced contraction or relaxation since colon diameter before and after tetrodotoxin was equivalent in *Bap1* WT and *TyrBap1* KO (Supplemental Figure 2, G and H).

### Loss of Bap1 in ENS alters bowel epithelium.

Deformed *TyrBap1* KO colon beyond P15 (Figure 1, D–G) suggested that normally flexible bowel might be fibrotic and/or inflamed. Stiff bowel could impair motility. To visualize collagen and anatomy, we evaluated trichrome-, hematoxylin and eosin–, and periodic acid–Schiff/Alcian blue–stained bowel (Supplemental Figure 3). *TyrBap1* KO and controls had similar amounts of collagen (Supplemental Figure 3, A–H) and immune infiltrates (Supplemental Figure 3, A–J, P–S, D′–G′, L′, and M′). In contrast, *TyrBap1* KO had more periodic acid–Schiff–positive goblet cells per crypt in distal small intestine and proximal colon (Supplemental Figure 3, R, S, A′, B′, F′, G′, and J′), reduced distal small bowel villus length (Supplemental Figure 3, R–T), and reduced villus/crypt cell ratio (Supplemental Figure 3, X and C′). These epithelial changes seem unlikely to cause colon deformity or dysmotility, but might result from ENS defects.

### Bap1 is not needed for fetal bowel colonization by ENS precursors nor for neonatal bowel motility.

**Figure 3:**
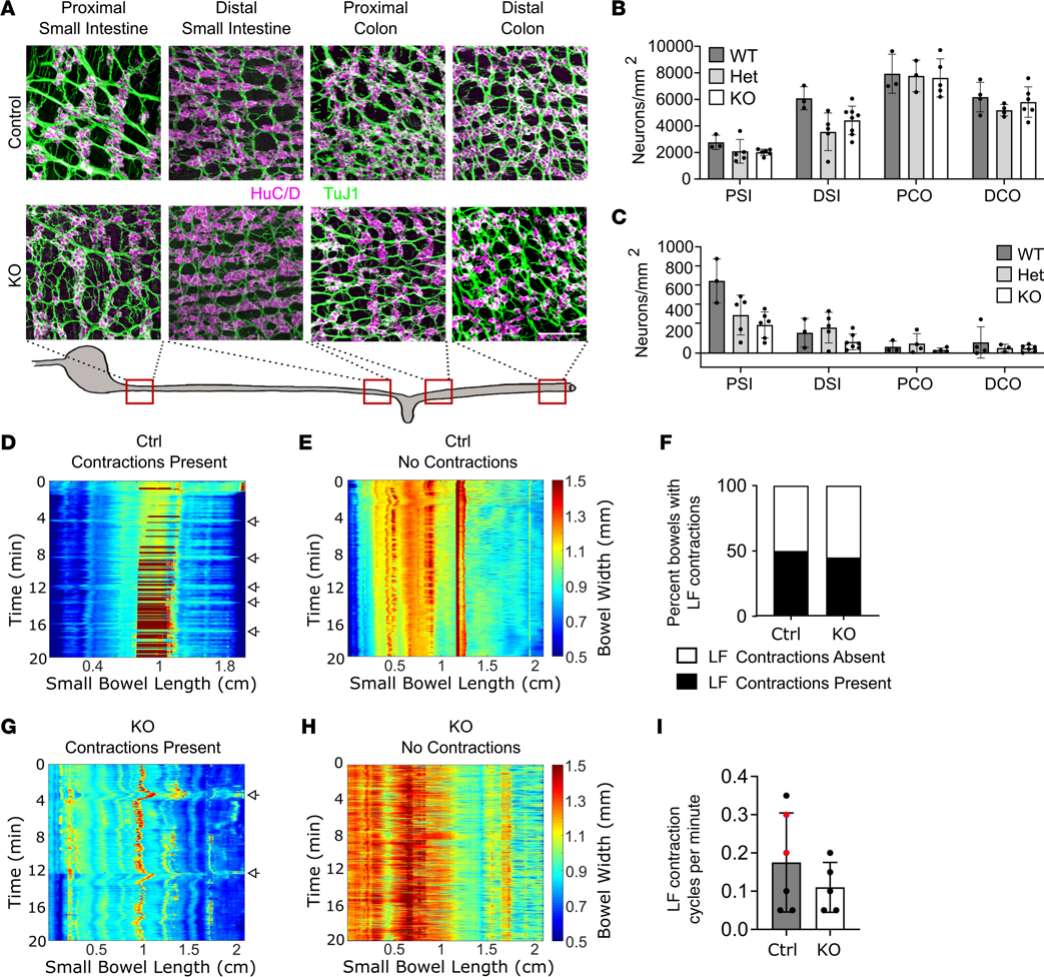
Neonatal *Wnt1Bap1^+^* mice have normal enteric neuron density and proximal small intestinal motility. (**A**–**C**) Enteric neuron density is similar to WT in most bowel regions in P0 *Wnt1Bap1* KO (KO). (**A**) Representative maximal-intensity projection *Z*-stacks of WT (top) and KO (bottom) along the bowel. Purple, HuC/D; green, TuJ1. Scale bar: 100 μm. (**B**) Quantification of P0 myenteric neuron density. (**C**) Quantification of P0 submucosal enteric neuron density. (**D**, **E**, **G**, and **H**) Representative kymographs depicting bowel width as a function of time and distance along proximal-distal axis proximal small intestine for P0 control (Ctrl; **D** and **E**) and KO (**G** and **H**). Low-frequency (LF) contractions (neurogenic) could be recorded for a subset of P0 Ctrl and KO pups (**D** and **G**, white arrows), while no LF contractions were detected for other Ctrl and KO (**E** and **H**). (**F**) Proportion of bowels where any LF contractions were recorded did not differ between P0 Ctrl and P0 KO. (**I**) Frequency of LF contractions did not differ between Ctrl and KO neonates if any LF contractions could be recorded. Data points from Hets are red. (**A**–**C**) WT refers to *Bap1^wt/wt^ Wnt1-Cre^+^* or any genotype without the *Wnt1-Cre* transgene. Het refers to *Bap1^fl/wt^ Wnt1-Cre^+^* genotype. KO refers to *Bap1^fl/fl^ Wnt1-Cre^+^* genotype. Data not significant unless otherwise indicated. (**D**–**I**) Ctrl refers to any genotype without the *Wnt1-Cre* transgene, or *Bap1^wt/wt^ Wnt1-Cre^+^* or *Bap1^fl/wt^ Wnt1-Cre^+^* genotype. KO refers to *Wnt1Bap1* KO genotype. (**B** and **C**) PSI, proximal small intestine; DSI, distal small intestine; PCO, proximal colon; DCO, distal colon. (**B**, **C**, and **I**) Data are shown as mean ± SD. (**B** and **C**) Welch’s ANOVA test with Dunnett’s T3 multiple-comparison test. (**I**) Two-sided Student’s *t* test.

*Bap1* impacts differentiation (39), supports *Sox10* and *Ednrb* expression (HSCR susceptibility genes) (33), and facilitates fetal melanocyte precursor migration (Figure 1, I and J). We therefore hypothesized that fetal *Bap1* loss would reduce bowel colonization by ENS precursors, causing neonatal HSCR-like distal bowel aganglionosis. To test this, we bred *Bap1* floxed to *Wnt1-Cre* to induce DNA recombination in almost all fetal ENS precursors starting at E8.5 (just before precursors enter bowel) (44). Surprisingly, neonatal *Bap1^fl/fl^ Wnt1-Cre^+^* (*Wnt1Bap1* KO) had normal-appearing ENS anatomy (Figure 3A) with normal myenteric neuron density in all regions (Figure 3, B and C). Small bowel motility in vitro also appeared similar in *Wnt1Bap1* KO and control littermates at birth. CMCs (low-frequency/neurogenic contractions) were observed in about half of *Wnt1Bap1* KO and controls as expected for neonates (45) (Figure 3, D–I, and Supplemental Figure 4, A–D). Frequency of CMCs (Figure 3I) and of myogenic high-frequency contractions was also similar for P0 *Wnt1Bap1* KO and controls (Supplemental Figure 4, E and F). Given significant motility defects observed by P15, we hypothesized that enteric neurons without BAP1 might be lost postnatally. Unfortunately, *Wnt1Bap1* KO die on P0 (Supplemental Figure 4G), so *TyrBap1* KO were analyzed to interrogate this hypothesis.

### Bap1 mutation causes progressive enteric neuron loss between P0 and P15 in TyrBap1 KO mice.

**Figure 4:**
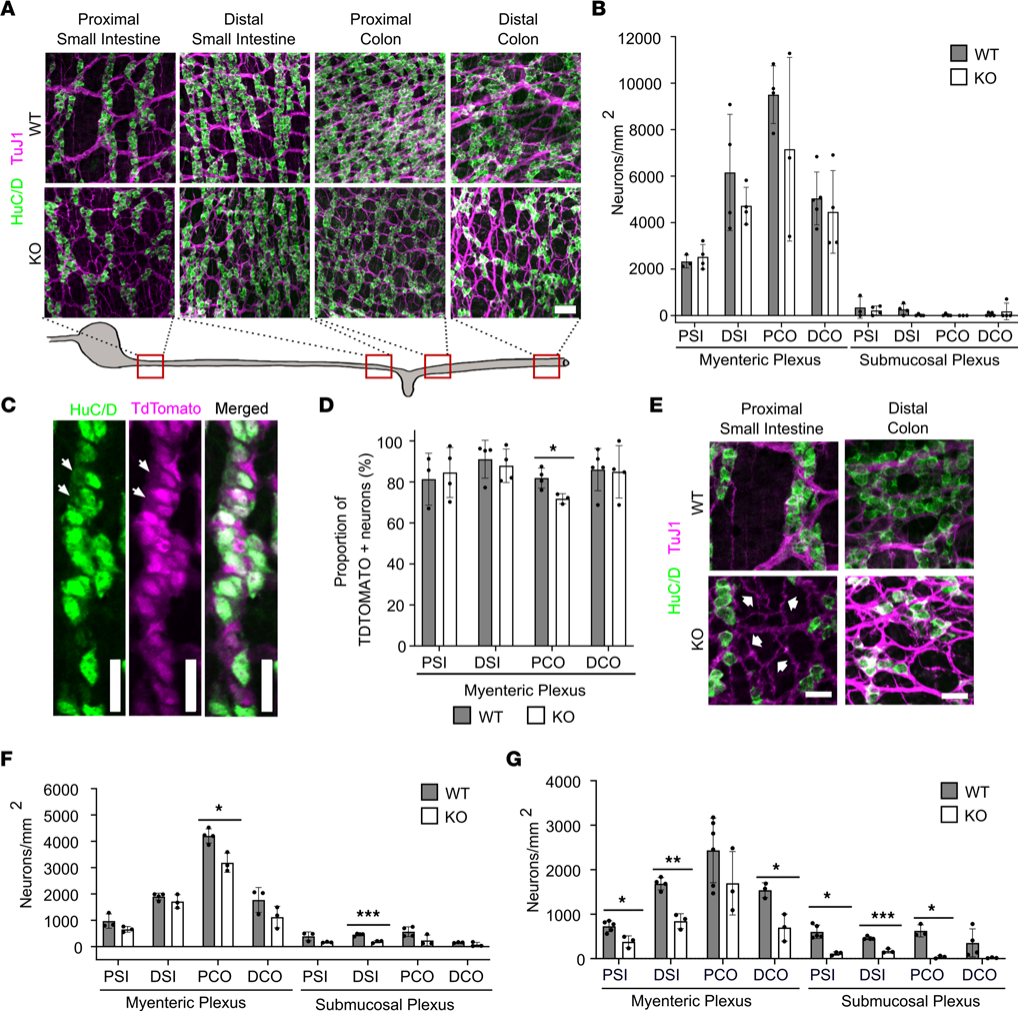
P0 mice have normal enteric neuron density with subtle differences in ENS anatomy, but neuron density declines with age. (**A**) Representative maximal-intensity *Z*-stacks of myenteric plexus from P0 WT and *TyrBap1* KO. Scale bar: 50 μm. (**B**) P0 *TyrBap1* KO enteric neuron density was similar to WT. (**C**) Representative maximal-intensity *Z*-stack of P0 myenteric plexus neurons (green, HuC/D^+^) and *Tyr-Cre* lineage (magenta, TdTomato^+^). Arrows highlight TdTomato-negative neurons. Scale bars: 25 μm. (**D**) Proportion of *Tyr-Cre*–lineage neurons is reduced in proximal colon of P0 *TyrBap1* KO versus WT. (**E**) Representative maximal-intensity projection *Z*-stacks illustrate subtle differences in P0 ENS of *TyrBap1* KO versus WT. Three images are enlarged from **A**. White arrows highlight fine neurite bundles connecting ganglia of some *TyrBap1* KO, whereas WT have fewer thicker fasciculated neurite bundles. Regional variation in distal colon ENS occurred in neonatal KO. Some regions had increased loosely connected neurons and/or small ganglia (bottom right) versus WT (top right). Scale bars: 25 μm. (**F**) Total neuron density in P5 *TyrBap1* KO is reduced in PCO myenteric and DSI submucosal plexus versus WT. (**G**) Neuron density in P10 *TyrBap1* KO is low in many myenteric and submucosal plexus regions versus WT. (**A**–**G**) WT, *Bap1^wt/wt^ Tyr-Cre^+^*. PSI, proximal small intestine; DSI, distal small intestine; PCO, proximal colon; DCO, distal colon. All data represent mean ± SD. *P < 0.05, **P < 0.01, ***P < 0.001. (**B**) Myenteric and submucosal PSI: Welch’s 2-tailed *t* test. Submucosal DSI: Mann-Whitney test. (**D**) Myenteric PSI: Welch’s 2-tailed *t* test. Myenteric DSI: Mann-Whitney test. (**F** and **G**) Welch’s 2-tailed *t* test except for (**G**) submucosal PSI, which used Mann-Whitney test.

**Figure 5:**
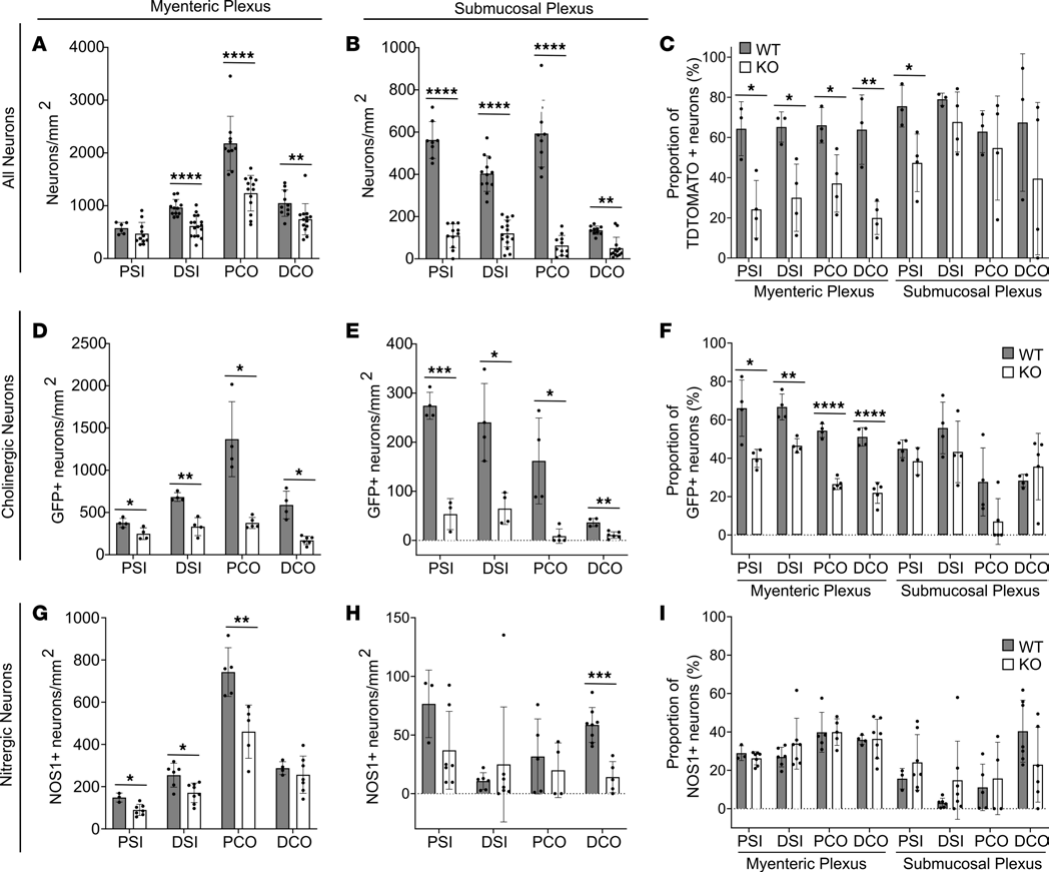
P15 *TyrBap1 KO* enteric neuron density is low in all regions with disproportionate *Tyr-Cre*–lineage and cholinergic neuron loss. (**A** and **B**) P15 *TyrBap1* KO neuron density is lower than WT in myenteric (**A**) and submucosal plexus (**B**) in all regions. (**C**) P15 *Tyr-Cre*–lineage (TdTomato^+^) myenteric plexus neurons are disproportionately reduced in all regions of *TyrBap1* KO. *Tyr-Cre*–lineage submucosal neurons are disproportionately reduced only in PSI. (**D** and **E**) P15 *TyrBap1* KO cholinergic neuron density is reduced in myenteric (**D**) and submucosal plexus (**E**) in all regions. (**F**) Cholinergic neurons are disproportionately reduced for P15 *TyrBap1* KO in all myenteric plexus regions but not in submucosal plexus. (**G** and **H**) P15 *TyrBap1* KO nitrergic neuron (NOS1-expressing neuron) density is reduced in all myenteric (**G**) and submucosal plexus (**H**) regions evaluated. (**I**) Proportion of nitrergic neurons is similar in *TyrBap1* KO and WT in all regions. (**D**–**F**) Cholinergic neurons are defined as GFP^+^ using *TyrBap1* *Chat-GFP* interbred lines. (**A**–**I**) WT is *Bap1^wt/wt^ Tyr-Cre^+^*. PSI, proximal small intestine; DSI, distal small intestine; PCO, proximal colon; DCO, distal colon. Data are represented as mean ± SD. *P < 0.05, **P < 0.01, ***P < 0.001, ****P < 0.0001. (**A** and **B**, PSI, DSI, and PCO): Unpaired 2-tailed *t* test with Welch’s correction. (**B**, DCO, and **E**): Two-tailed Mann-Whitney test. (**C**) Two-tailed unpaired *t* test with Welch’s correction. (**D** and **G**) Welch’s 2-tailed unpaired *t* test. (**F**) Welch’s 2-tailed unpaired *t* test, except for PCO, which used 2-tailed Mann-Whitney test. (**H**) Welch’s 2-tailed unpaired *t* test, except PSI, which used Mann-Whitney test. (**I**) Welch’s 2-tailed unpaired *t* test, except DSI, which used 2-tailed Mann-Whitney test.

*TyrBap1*^+^ induces Cre-mediated DNA recombination in about 80% of ENS precursors starting at E10.5 (41). Like *Wnt1Bap1* KO, neonatal *TyrBap1* KO had nearly normal-appearing ENS anatomy (Figure 4A) with normal neuron density in all regions examined (Figure 4B). Neonatal TdTomato^+^ neuron counts were also normal (except 11% proximal colon reduction) in *TyrBap1* KO *TdTomato^+^* mice that express *TdTomato* after Cre-mediated DNA recombination (Figure 4, C and D). Some neonatal *TyrBap1* KO appeared to have subtly altered ENS neurite organization (Figure 4E), but high variability made quantitative analysis challenging. Between P5 and P10, *TyrBap1* KO *TdTomato^+^* had progressive enteric neuron loss (Figure 4, F and G). By P15, *TyrBap1* KO had 29%–43% fewer myenteric neurons in almost all bowel regions (Figure 5, A and B, and Supplemental Figure 5) with preferential TdTomato^+^ neuron loss (Figure 4C and Figure 5C). P15 submucosal neuron loss was even more profound (63%–89%), but percentage of submucosal TdTomato^+^ neurons was similar in P15 *TyrBap1* KO and WT (Figure 5C). Cholinergic neurons were also lost preferentially from P15 myenteric and submucosal plexus of *TyrBap1* KO with relative nitrergic neuron preservation (Figure 5, D–I, and Supplemental Figure 6).

**Figure 6:**
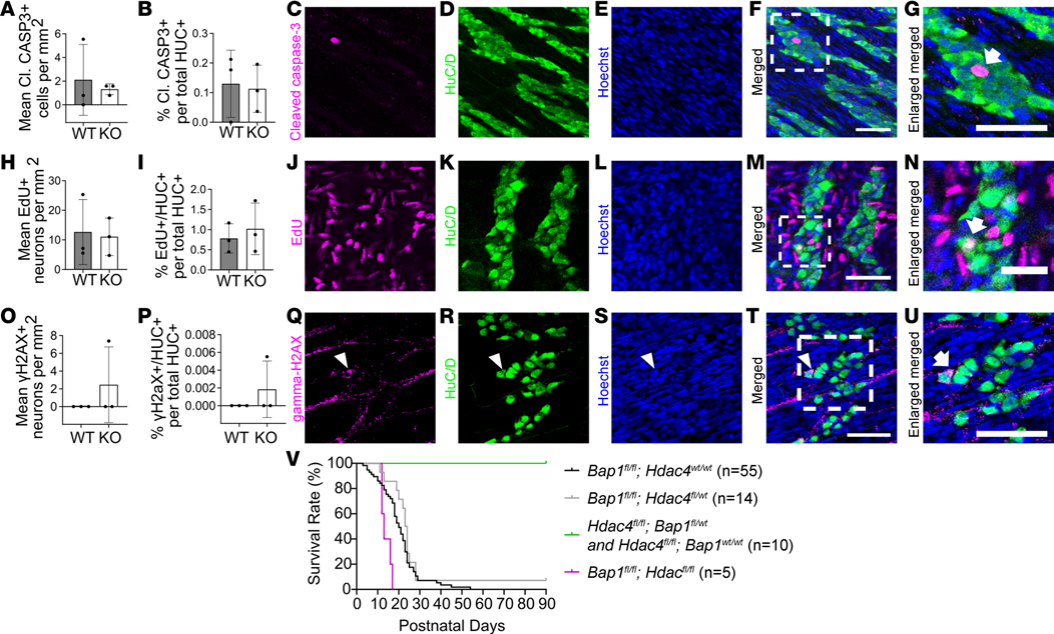
No evidence of increased apoptotic neuron loss, reduced neurogenesis, or increased DNA damage in distal colon of *TyrBap1* KO; no change in *TyrBap1* KO *Hdac4^–/–^* survival. (**A**) Cleaved caspase-3^+^ intraganglionic cell density is similar in P5 *TyrBap1* KO and WT. (**B**) Percentage cleaved caspase-3^+^ intraganglionic neurons is similar in P5 *TyrBap1* KO and WT. (**C**–**G**) Representative maximal-intensity *Z*-stacks show a cleaved caspase-3^+^ intraganglionic cell. (**C**) Purple, cleaved caspase-3. (**D**) Green, HuC/D. (**E**) Blue, Hoechst. (**F**) Merged. (**G**) Enlargement of region marked with dashed line in **F**. Arrow identifies intraganglionic cleaved caspase-3–expressing cell. Scale bars: 50 μm. (**H**) EdU^+^ neuron density is similar in P7 *TyrBap1* KO and WT that received daily EdU injections from P4 to P6. (**I**) Percentage EdU^+^ neurons. (**J**–**N**) Representative maximal-intensity *Z*-stacks show an EdU^+^ neuron at P7. (**J**) Purple, EdU. (**K**) Green, HuC/D. (**L**) Blue, Hoechst. (**M**) Merged. (**N**) Enlarged region marked with dashed line in **M**. Arrow points to EdU^+^ neuron. Scale bars: 50 μm, 25 μm in **N**. (**O**–**U**) DNA damage was not detectable in P5 *TyrBap1* KO or WT ENS by γH2AX staining. (**O**) γH2AX^+^ neuron density. (**P**) Percentage γH2AX^+^ neurons. (**Q**–**U**) Maximal-intensity *Z*-stack of P5 distal colon myenteric plexus shows one γH2AX^+^ neuron (arrowheads, arrow). (**Q**) Purple, γH2AX. (**R**) Green, HuC/D. (**S**) Blue, Hoechst. (**T**) Merged. (**U**) Enlarged region marked with dashed line in **T**. Arrow points to γH2AX^+^ neuron. Scale bars: 50 μm. (**V**) *Hdac4* loss did not rescue *TyrBap1* KO phenotype. *TyrBap1 Hdac4^fl/fl^* mice die early with massively dilated bowel. *Tyr-Cre^+^ Bap1^wt/wt^ Hdac4^fl/fl^* mice have shortened faces and malocclusion and may survive into old age with tooth trims. (**A**–**P**) Data are shown as mean ± SD. (**A**, **B**, **H**, **I**, **O**, and **P**) Unpaired 2-tailed *t* test. (**V**) Log-rank (Mantel-Cox) test.

To determine why neuron density is reduced in *TyrBap1* KO, we focused on distal colon. At P5, *TyrBap1* KO distal colon myenteric plexus neuron density was statistically equivalent to WT, although mean neuron density was 37% lower (Figure 4F). By P10, *TyrBap1* KO had 55% fewer myenteric neurons (Figure 4G and Figure 5A). We reasoned that neuron loss probably started by P5, and pursued mechanistic studies at this age. We found very few (0.1%) cleaved caspase-3–positive neurons (HuC/D^+^) in *TyrBap1* KO or WT (Figure 6, A–G). The number of P4–P6 myenteric neurons (HuC/D^+^) that incorporated ethynyl-2′-deoxyuridine (EdU) was equivalent in *TyrBap1* KO and WT (Figure 6, H–N). DNA damage in P5 myenteric neurons was equivalent based on γH2AX staining (Figure 6, O–U). Finally, because histone deacetylase *hdac4* loss rescues gastrulation and neurulation defects in *Xenopus bap1* morphants (39), we generated *TyrBap1* KO *Hdac4^–/–^* mice, but they did not live longer than *TyrBap1* KO (Figure 6V). Collectively, these studies failed to explain progressive postnatal reductions in enteric neuron density in *TyrBap1* KO.

### Prenatal Bap1 loss dramatically alters enteric neuron differentiation.

**Figure 7:**
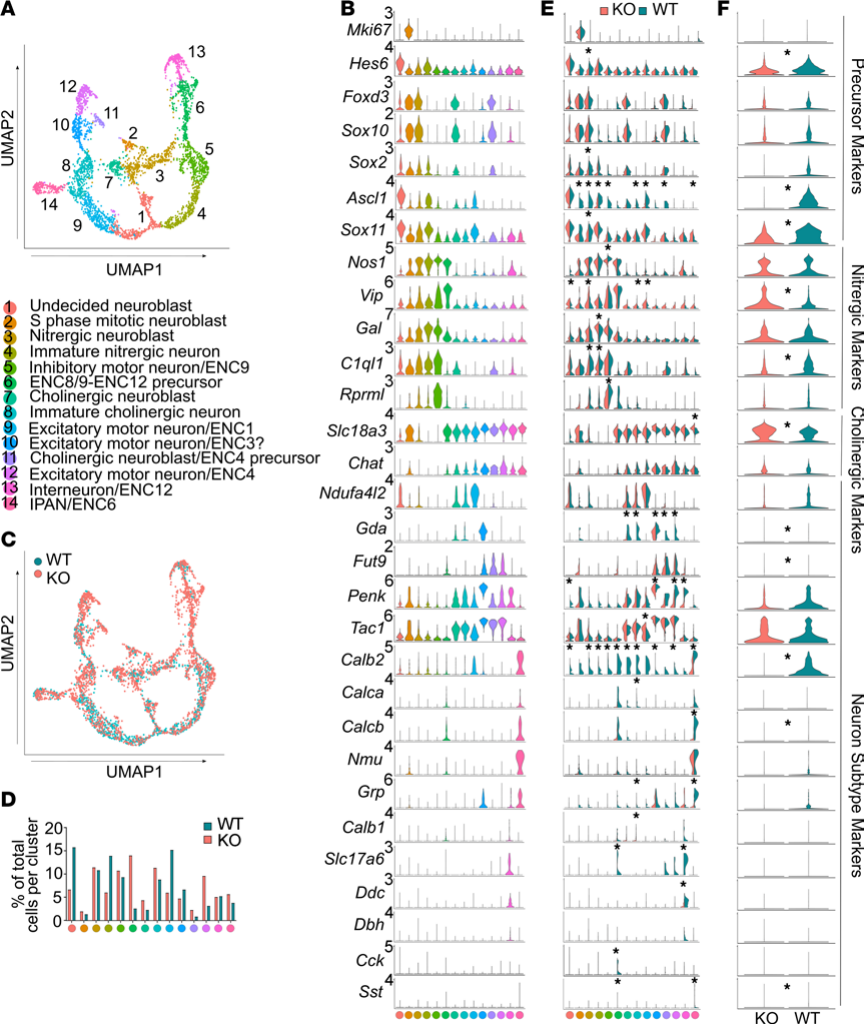
Enteric neuron subtype ratios and gene expression are abnormal at P5 in *Tyr-Cre*–lineage neurons of *TyrBap1* KO mice. (**A**) UMAP projection of 14 enteric neuron subtypes identified in P5 colon myenteric plexus using unsupervised clustering (Seurat single-cell sequencing analysis pipeline). Each dot represents a single cell, and color indicates neuron subtype (cluster) identity. (**B**) Violin plots show expression levels of select neuron subtype and precursor markers in each cluster. (**C**) UMAP projection shown in **A**. Blue color denotes cells from *Bap1^wt/wt^ Tyr-Cre^+^* (WT) tissue. Red color denotes cells derived from *TyrBap1* KO tissue. (**D**) Percentage of total cells in each individual cluster for WT or *TyrBap1* KO. (**E**) Expression levels of selected genes in individual WT or *TyrBap1* KO clusters color-coded by genotype (blue, WT; red, *TyrBap1* KO). (**F**) Expression levels of selected genes across all WT or *TyrBap1* KO neurons (blue, WT; red, *TyrBap1* KO). (**B**, **E**, and **F**) Expression level represents ln(normalized and scaled expression level) where mean expression level for each gene across all cells in data set is defined as ln(1). (**E** and **F**) Bonferroni-corrected statistical significance (defined as *P* < 0.05) is indicated by asterisks (exact *P* values are accessible in the Supplemental Data file).

To further explore mechanisms of ENS dysfunction, we pursued scRNA-Seq of *TdTomato^+^* P5 *TyrBap1* lineage–marked cells from colon muscularis. Using pooled data from 2,382 *TyrBap1* KO and 1,392 WT enteric neurons (18.91% of *TyrBap1* KO single cells and 17.52% of WT single cells), we performed principal component analysis–based dimensionality reduction and clustering (visualized as uniform manifold approximation and projection [UMAP]; Figure 7A). Neuron clusters were classified based on recent subtype categories (11) (Figure 7, A and B, and Supplemental Figure 7A). While many clusters had readily identifiable mature neuron phenotypes (Figure 7B), others appeared immature (neuroblast-like) based on simultaneous expression of cell cycle–related genes (Supplemental Figure 7, B and C), enteric precursor cell/neuroblast markers (*Sox2*, *Sox10*, *Sox11*, *Foxd3*, *Ascl1*, *Hes6*, *Tox3*, *Insm1*, *Bcl11b*, *St18*, and *Wwtr1*) (11) (Figure 7B and Supplemental Figure 7C), and reduced expression of neurotransmitters or established subtype markers (Figure 7B and Supplemental Figure 7D). Actively cycling, neuroblast-like cells were more abundant in WT than in *TyrBap1* KO (Supplemental Figure 7B), and some clusters were primarily WT cells (e.g., undecided neuroblast, immature inhibitory motor neuron, excitatory motor neuron/ENC1). In other clusters, *TyrBap1* KO predominated (ENC8/9–ENC12 precursor, excitatory motor neuron/ENC4) or there were similar numbers of *TyrBap1* KO and WT cells (e.g., S-phase mitotic neuroblast, nitrergic neuroblast, inhibitory motor neuron/ENC9, immature nitrergic neuron, immature cholinergic neuron, excitatory motor neuron/ENC3?, interneuron/ENC12, and IPAN/ENC6; Figure 7, C and D).

Remarkably, even for cells in the same cluster, expression of neuron subtype markers often varied dramatically between *TyrBap1* KO and WT neurons (Figure 7, B and E). For example, in IPAN/ENC6, *Calb2*, *Calcb*, *Grp*, and *Sst* transcripts were more abundant in WT than *TyrBap1* KO (Figure 7, E and F). Similarly, in interneuron/ENC12, glutamate transporter (*Slc17a6*) and DOPA decarboxylase (*Ddc*) mRNA were much more abundant in WT, even though WT and *TyrBap1* KO cells were approximately equally abundant (Figure 7, B, D, and E). Moreover, *Calb2*, *C1ql1*, *Ascl1*, and *Hand2* were expressed in many cell clusters but were much more abundant in WT than *TyrBap1* KO for most clusters. Similarly, *Ednrb* (which supports ENS precursor proliferation and migration) (46) and *Tgfb2* (which supports muscularis macrophage and enteric neuroblast identity) (11) were more abundant in WT than in *TyrBap1* KO neurons, as was *Ntrk3* (NT3 receptor; supports nitrergic neuron differentiation) (47) in ENC8/9–ENC12 precursors or IPAN/ENC6 neurons (Supplemental Figure 7E), and *Gfra2* (neurturin receptor) (48) in mature colonic cholinergic excitatory motor neurons/ENC4. In contrast, *Vip* and *Slc18a3* were more abundant in *TyrBap1* KO myenteric neurons than in WT. *Rarb* (21) and *Cntfr* (47), which influence enteric neuron subtype differentiation, were also more abundant in *TyrBap1* KO compared with WT neurons. The many differences in gene expression between WT and *TyrBap1* KO are likely to affect neuron function, differentiation, and survival. Consistent with this hypothesis, RNA velocity analysis suggested that *Bap1* loss impaired normal differentiation trajectories for many enteric neuron subtypes (Supplemental Figure 7, F–H). Finally, Molecular Signatures Database (MSigDB) pathway overlap analysis showed that compared with controls, *TyrBap1* KO enteric neurons had reduced mRNA for many genes involved in translation, rRNA processing, and mRNA processing, with relative enrichment for genes involved in glucose metabolism, the immune system, and RHO GTPases (Supplemental Figure 8).

### Prenatal Bap1 loss has only minor effects on enteric glia.

In contrast to enteric neurons, *TyrBap1* KO enteric glia had more subtle changes in gene expression, and glial cell density was normal at P15 (Supplemental Figure 9). Single-cell RNA-Seq showed altered ratios of some glial subtypes in *TyrBap1* KO (higher percentages of enteric glia type 1 and proinflammatory or apoptotic glia, and lower percentages of glial types 2 and 3 and early neurons) (Supplemental Figures 9 and 10). Fast Gene Set Enrichment Analysis (FGSEA) failed to identify any significant pathways when all glial clusters were analyzed. MSigDB overlap analysis indicated that *TyrBap1* KO enteric glia had reduced mRNA encoding translational and mRNA processing machinery (like enteric neurons), and elevated levels of mRNA in proinflammatory and extracellular matrix pathways (Supplemental Figure 11).

### Postnatal Bap1 loss in ENS does not affect survival, weight gain, or colon motility.

**Figure 8:**
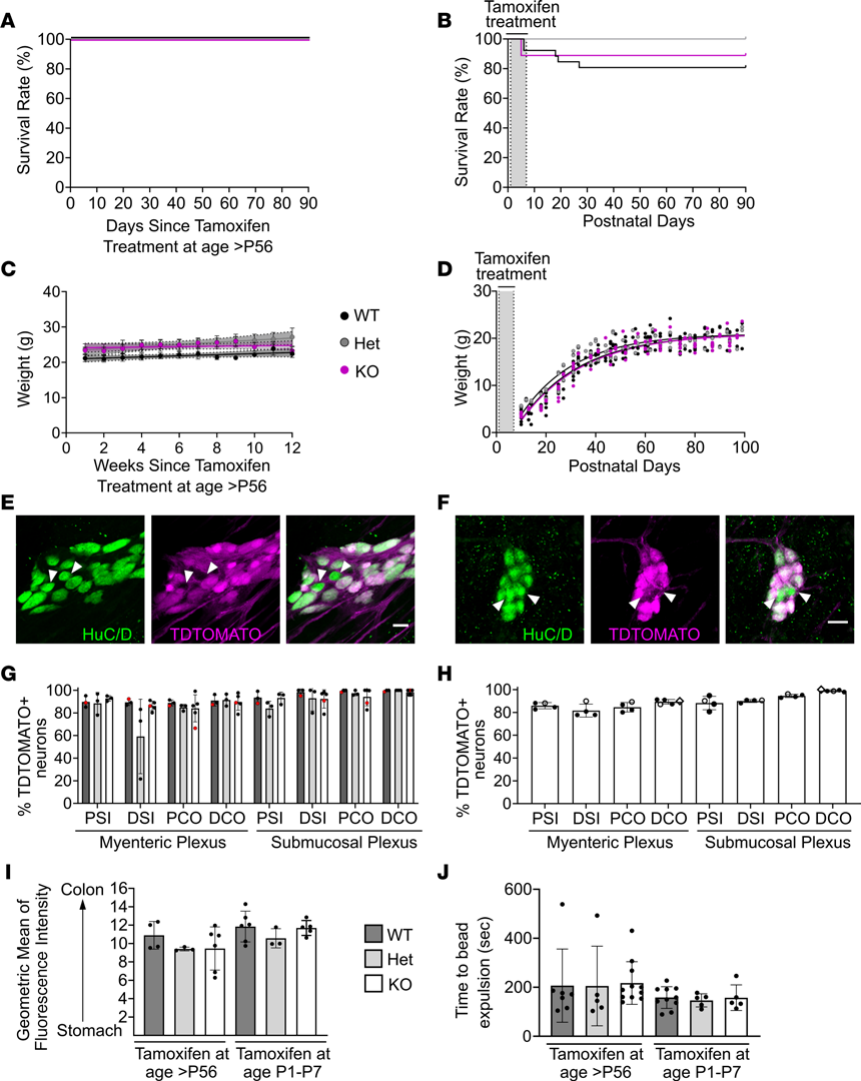
Tamoxifen-induced postnatal *Bap1* loss in *RetCreERT2Bap1* KO ENS did not affect survival, weight, bowel motility, or *Ret-CreERT2*–lineage neuron density. (**A** and **B**) *Bap1* loss after P56–P63 (**A**) or P1–P7 (**B**) tamoxifen did not cause death in *RetCreERT2Bap1* KO observed ≥90 days. (**C**) P56–P63 tamoxifen-treated *RetCreERT2Bap1* KO maintained weight like WT. (**D**) P1–P7 tamoxifen-treated *RetCreERT2Bap1* KO gained weight normally. (**E** and **F**) Representative single confocal planes from 9-week-old *RetCreERT2Bap1* KO proximal colon myenteric (**E**) and submucosal (**F**) plexus (1 week after tamoxifen). Green, HuC/D; magenta, TdTomato. Scale bars: 20 μm. Arrowheads highlight neurons without TdTomato. (**G**) P56–P63 tamoxifen induced Cre-mediated recombination in most neurons. Black dots, >90 days after tamoxifen; red dots, 1 week after tamoxifen. Similar proportions of *Ret-CreERT2*–lineage neurons in WT and *RetCreERT2Bap1* KO suggest that *Bap1*-deficient neurons are maintained when *Bap1* loss occurs in adults. (**H**) Cre-mediated DNA recombination occurred within 3 days of completion of P1–P7 tamoxifen in KO (filled circles), Het (open circles), and WT (open diamonds). Analysis at P8 or P9. (**I**) FITC-dextran transit through proximal small intestine was normal after *Bap1* loss in tamoxifen-treated *RetCreERT2Bap1* KO at >P56 or P1–P7. *Y* axis is numbered from proximal small intestine (segment 2) to mid-colon (segment 16). Analysis >90 days after tamoxifen, 90 minutes after FITC-dextran. (**J**) Colon bead expulsion latency was normal for *RetCreERT2Bap1* KO after adult (>P56) or P1–P7 tamoxifen. Testing was >90 days after tamoxifen. (**A**–**J**) WT, *Bap1^wt/wt^ Ret^wt/CreERT2^*, or any genotype without *CreERT2*. Het, *Bap1^fl/wt^ Ret^wt/CreERT2^*. KO, *RetCreERT2Bap1*. Data are shown as mean ± SD. (**A**) Unable to calculate log-rank (Mantel-Cox) test due to zero deaths. (**B**) Log-rank (Mantel-Cox) test. (**C**) Simple linear regression. (**D**) Repeated-measures 1-way ANOVA mixed-effects model with multiple comparisons. (**G**) Ordinary 1-way ANOVA. (**I** and **J**, P1–P7 tamoxifen): Brown-Forsythe ANOVA test. (**J**, adult tamoxifen): Kruskal-Wallis test.

The absence of obvious neonatal ENS defects, coupled with striking progressive postnatal ENS abnormalities, suggested that *Bap1* is not needed before birth in the ENS lineage. To test this hypothesis, we deleted *Bap1* from ENS in perinatal or adult *Bap1^fl/fl^ Ret-CreERT2^+^* (called *RetCreERT2Bap1* KO) mice by giving tamoxifen (P1–P7 or P56–P63). At both ages, tamoxifen-treated *RetCreERT2Bap1* KO survived as long as WT (Figure 8, A and B), maintained normal weight (8-week-old treated) or grew like WT (P1–P7 treated) (Figure 8, C and D), and appeared healthy, despite Cre-mediated DNA recombination in 80%–90% of enteric neurons (Figure 8, E–H). Furthermore, FITC-dextran transit through small bowel after oral gavage (Figure 8I) and colonic bead expulsion (Figure 8J) were normal in tamoxifen-treated *RetCreERT2Bap1* KO mice. These observations suggest that *Bap1* is required before birth for normal postnatal ENS development, maintenance, and function. Furthermore, while *Bap1* deficiency phenotypes manifest shortly after birth, *Bap1* is not needed in the ENS during the postnatal interval of progressive ENS dysfunction.

## Discussion

BAP1 regulates gene expression by reversing H2AK119 mono-ubiquitination, a repressive mark produced by PRC1 (18), and by altering activity of some transcription factors (36). BAP1 also impacts DNA replication fork elongation, DNA damage repair, and IP3R3 stability (enhancing apoptosis if DNA damage is excessive) (18). Consistent with these roles, *Bap1* is essential for early embryonic development, with roles in germ layer lineage commitment. *BAP1* mutations predispose to uveal melanoma, renal cell carcinoma, and mesothelioma (18). We are not aware of prior data linking *BAP1* to bowel motility disorders or the ENS. However, our data suggest that prenatal BAP1 deficiency in ENS lineages causes dramatic postnatal enteric neuron loss, severe bowel dysmotility, and early death.

*TyrBap1* KO mice were originally created to generate a uveal melanoma model since tyrosinase is expressed in neural crest–derived melanocytes, but no tumors were observed. Instead, *TyrBap1* KO had only mild coat pigmentation defects (Figure 1I). ENS phenotypes arise because *TyrBap1* KO undergo Cre-mediated recombination starting at E10.5–11.5 in lineages that generate approximately 85% of neonatal myenteric and approximately 70% of P15 myenteric and submucosal neurons. At E10.5, vagal crest–derived ENS precursors have just entered the stomach and started to migrate toward distal colon in WT mice (41). Remarkably, despite fetal *Bap1* loss, *TyrBap1* KO had normal-appearing ENS throughout the bowel at birth, as did *Wnt1Bap1* KO that lack *Bap1* in all prenatal ENS precursors starting at E8.5. Furthermore, small bowel contractility at P0 seemed similar in *Wnt1Bap1* KO and WT. Thus, *TyrBap1* KO and *Wnt1Bap1* KO are not HSCR models.

Enteric neuron numbers began to decline shortly after birth in *TyrBap1* KO, with reductions in some regions by P5 (Figure 4F). At P15, neuron density is reduced dramatically throughout *TyrBap1* KO bowel (29%–89% reductions compared with WT) and bowel motility is profoundly impaired, plausibly explaining 50% mortality by P20. The most dramatic reductions occurred in neurons marked by TdTomato in *TyrBap1* KO *TdTomato* reporters, suggesting primarily cell-autonomous BAP1 roles in ENS lineages as might be expected. However, many TdTomato-negative neurons were lost in submucosal plexus, suggesting that these cells rely on *TyrBap1* lineage–derived trophic factors. Mechanisms underlying reduced neuron numbers in *TyrBap1* KO remain uncertain. We did not find evidence of increased apoptosis (cleaved caspase-3, RNA-Seq), reduced proliferation (EdU), or increased DNA damage (γH2AX) at P5 when reduced neuron numbers were apparent. It is possible that changes in neuron number occur gradually (e.g., 5% each day over 10 days). In this case, causes of neuron loss could be difficult to define (i.e., too small to measure on any given day). In support of this hypothesis, scRNA-Seq at P5 indicates a 4.42% reduction in cycling neuroblasts in *TyrBap1* KO compared with controls (Supplemental Figure 7B). As one possible explanation for reduced cycling neuroblasts, we noted reduced *Ednrb* in some clusters (e.g., ENC8/9–ENC12 precursors, cholinergic neuroblasts; Supplemental Figure 7E). EDNRB promotes enteric neuron precursor proliferation and migration during fetal development (46). While these proliferation differences might contribute to differences in neuron density, even with 3 days of EdU labeling, only 0.7%–1% of HuC/D^+^ myenteric neurons incorporated EdU (<0.33% Edu^+^ per day), making it unlikely that differences in proliferation alone could explain markedly reduced P15 neuron density in *TyrBap1* KO mice.

Even if enteric neuron numbers were normal, *TyrBap1* KO mice are expected to have impaired bowel motility because of abnormal enteric neuron subtype differentiation. In addition, markedly reduced *Tgfb2* in many *TyrBap1* KO neuron subtypes (Supplemental Figure 7E) should reduce neuron-associated muscularis macrophages (NA-MMΦ) (49) that refine ENS synaptic connections. However, loss of NA-MMΦ function at this stage (P10) cannot explain enteric neuron loss and instead should increase enteric neuron numbers.

The reduced neuron density, altered ENS gene expression, severe dysmotility, and early death in *TyrBap1* KO mice provide what we believe to be a new model for the human disease called neuropathic chronic intestinal pseudo-obstruction (nCIPO). nCIPO is rare and may have onset in infancy, childhood, or adulthood. With few exceptions, causes of nCIPO remain poorly defined. *TyrBap1* KO mice have dramatic reductions (50%–70%) in undecided neuroblasts, immature nitrergic neurons, and excitatory motor neurons/ENC1, but markedly increased numbers of excitatory motor neurons/ENC4 and of ENC8/9–ENC12 precursors rarely seen in P5 WT. Furthermore, gene expression differs markedly between *TyrBap1* KO and WT neurons even within single clusters. For example, compared with WT, *TyrBap1* KO neurons had less mRNA for many neurotransmitters (*Penk*, *Grp*, *Cartpt*, *Sst*), enzymes that make neurotransmitters (*Ddc*), neurotransmitter receptors (*Oprk1*, *Cnr1*, *Kctd12*), proteins that modulate neurotransmitter receptors (*Lypd1*, *Gpc6*, *Crip1*, *Rgs4*, *Pirt*), neurotransmitter transporters or synaptic vesicle sorting proteins (*Slc17a6*, *Slc10a4*, *Scg2*), ion channel regulators (*Phactr1*, *Fgf13*, *Akap7*), calcium buffering proteins (*Calb2*, *Calb1*, *Calca*, *S100b*), proteins important for dendrite formation (*Gda*), axon guidance proteins (*Epha5*, *Nrp2*, *Sema3c*), cell adhesion–related proteins (*Chl1*, *Alcam*, *Cd9*), transcription factors important for nervous system function or development (*Ascl1*, *Hand2*, *Nfib*, *Zfhx3*, *Sox4*, *Tcf4*, *Tbx3*, *Tlx2*, *Casz1*, *Pbx3*), proteases or protease inhibitors critical for nervous system morphogenesis (*Adamts5*, *Serpini1*, *Serpine2*), proteins that regulate neural stem cell proliferation (*Btg1*, *Id4*), and growth factors or growth factor receptors (*Ednrb*, *Ntrk3*, *Gfra2*, *Tgfb2*, *Fgf13*, *Ngf*, *Kitl*). In contrast, compared with WT, *TyrBap1* KO neurons expressed more mRNA for other neurotransmitters and neuropeptides (*Vip*, *Pcsk1n*), enzymes that make or degrade neurotransmitters (*Slc18a3*, *Ache*, *Ass1*), proteins that regulate neurotransmitter receptor function (*Scgn*, *Ly6h*, *Caly*, *Cnih2*, *Necab2*), neural signaling proteins (*Crabp1*), cytoskeletal proteins impacting axon transport or nervous system development (*Prph*, *Tubb2a*, *Tubb3*, *Nefl*, *Nefm*), other proteins that influence nervous system development (*Olfm1*, *Pcp4*), trophic factors and trophic factor receptors (*Vgf*, *Pdgfa*, *Cntfr*, *Rarb*), vesicle-associated proteins (*Ndrg4*, *Rab3a*, *Vamp2*, *Syp*), and proteins that protect from oxidation (*Gpx3*, *Ngb*). Interestingly, compared with WT, RNA from *TyrBap1* KO neurons also encoded high levels of a long noncoding RNA (lncRNA) that promotes neuron death (*Meg3*) and lower levels of neuroprotective mRNA (*Cryab*, *Cst3*, *Timp3*), lncRNA with neuroprotective or neuronal differentiation roles (*Malat1*, *Hotairm1*), and an mRNA that prevents neuronal cell senescence (*Satb1*). Remarkably, MSigDB overlap analysis did not highlight these changes in mRNA, but instead showed that downregulated mRNAs were in pathways involved in protein and mRNA synthesis while upregulated mRNAs belonged to pathways for glucose metabolism, RHO GTPases, and the immune system. These data support the hypothesis that *Bap1* loss in the *TyrBap1* lineage disrupts ENS structure and function in many ways and that progressive postnatal reductions in enteric neuron density were due, at least in part, to altered expression of neuroprotective or neurodegeneration-inducing genes.

In contrast to dramatic changes in *TyrBap1* KO enteric neurons, *TyrBap1* KO enteric glia (which also lack BAP1) had subtle changes in gene expression. In fact, FGSEA identified no pathways associated with differentially expressed genes. There were, however, shifts in relative abundance of glial subtypes with more inflammatory glia and fewer early neurons in *TyrBap1* KO. Furthermore, MSigDB overlap analysis highlighted that *TyrBap1* KO glia had reduced mRNA in pathways for ribosomes, translation, and RNA processing, similar to findings highlighted in enteric neurons.

### BAP1 and the Polycomb repressor complex (PRC).

One striking aspect of the *Bap1* phenotype is that fetal *Bap1* loss in ENS lineages does not appear to affect neonatal ENS structure, bowel motility, or survival. Instead, prenatal *Bap1* loss profoundly impairs postnatal enteric neuron differentiation and induces postnatal enteric neuron loss. In contrast, mice with postnatal *Bap1* loss in most enteric neurons appear to remain healthy. Although BAP1 can directly regulate gene expression, this pattern suggests critical epigenetic roles for BAP1 during ENS cell lineage specification. Mechanistically, BAP1 deubiquitinates histone 2AK119-ub1 (H2AK119-ub1), an epigenetic mark that represses gene expression and can have long-lasting effects on transcription. H2AK119-ub1 is a modification created by PRC1 that is recruited to DNA after histone 3 K27 trimethylation (H3K27me3) by PRC2 (18). Inactivating mutations of PRC2-variant subunits *Aebp2* or *Ezh2* as well as defects in RA signaling (regulating PRC1/PRC2 activity) cause distal bowel aganglionosis in mice (12, 13, 20, 21). PRC1 includes CBX4, a protein stabilized by SALL1, which reduces CBX4 ubiquitination and proteasomal degradation (24). *SALL1* mutations cause Townes-Brocks–like syndrome, a disorder in which HSCR appears common (23). In addition, loss of PRC1 subunit *Pcgf1* in fetal ENS precursors impairs enteric neuron subtype specification, reducing somatostatin-expressing (*Sst*-expressing) (descending/inhibitory) neurons and increasing calbindin^+^ (*Calb1*^+^) (ascending/excitatory) neurons (26). Since BAP1 removes ubiquitin from H2AK119ub1 placed by PRC1, our observation that *Calb1* mRNA is lower in *TyrBap1* KO than WT fits with *Pcgf1^–/–^* data. However, we found fewer cells expressing *Sst1*, which is not expected if BAP1 only reversed PRC1-mediated H2AK119 ubiquitination (26). Collectively, these observations show critical roles for PRC1/PRC2 in the ENS. Our data show, what we believe to be for the first time, that epigenetic defects in prenatal ENS could cause postnatal ENS neurodegenerative phenotypes mimicking human nCIPO.

Our ENS observations may be relevant to a broad range of human neurodevelopmental and neurodegenerative diseases because BAP1 impacts PRC activity (17, 18). *EZH2*, *EED2*, and *SUZ12* mutations cause the neurodevelopmental disorders Weaver syndrome (OMIM #277590) (28), Cohen-Gibson syndrome (OMIM #617561) (30), and Imagawa-Matsumoto syndrome (OMIM #618786) (29). Mutations in *EZH1* (27) and in *KDM6B* (OMIM #611577) (32) and *KDM6A* (31) (H3K27 demethylases) cause syndromic neurodevelopmental disorders and Kabuki syndrome (OMIM #147920 and #300867). Altered PRC activity is implicated in ataxia-telangiectasia (50) and Huntington’s disease (51). Despite these links, only 2 prior studies suggested that BAP1 might impact nervous system function. Perhaps most interestingly, Küry et al. in 2022 described 11 individuals with de novo *BAP1* variants who had speech delay, hypotonia, behavioral problems, gross motor delay, feeding difficulty, and seizures (42). Disease-causing BAP1 variants impaired deubiquitinase activity, leading to elevated ubiquitin on H2A. Küry et al. point out that, to their knowledge, “*BAP1* is one of the rare genes in which different germline variants cause either a neurodevelopmental disorder or a tumor predisposition syndrome.” Future studies are warranted to investigate precise mechanisms through which BAP1 regulates ENS development and differentiation, and to identify additional BAP1 roles in the nervous system.

## Methods

Additional methods are available in Supplemental Methods.

### Sex as a biological variable.

Our study examined male and female animals, and similar findings are reported for both sexes.

### Mice.

*Bap1tm1.1Geno* (called *Bap1*; C57BL/6) mice were generated in-house (52). *Tg(Tyr-Cre)1Gfk* (called *Tyr-Cre*; RRID:MGI:3580524, C57BL/6) were a gift from Graham F. Kay (Queensland Institute of Medical Research, Herston, Queensland, Australia), *Hdac4tm2.1Eno* (called *Hdac4*; RRID:MGI:4418117, C57BL/6) a gift from Kelly A. Hyndman (University of Alabama at Birmingham, Birmingham, Alabama, USA), *B6.FVB(Cg)-Tg(Chat-EGFP/Rpl10a,Slc18a3)DW167Htz/J* (called *Chat-GFP*; RRID:IMSR_JAX:030250, C57BL/6J) a gift from Joseph Dougherty (Washington University School of Medicine in St. Louis, St. Louis, Missouri, USA), and *Rettm2(cre/ERT2)Ddg* (called *Ret-CreERT2*; RRID:MGI:4437245, C57BL/6) a gift from Jeffrey Milbrandt (Washington University School of Medicine in St. Louis). *H2az2(Tg(Wnt1cre)11Rth* [called *Wnt1-Cre*; RRID:IMSR_JAX:003829, (C57BL/6J × CBA/J)F_1_]) and *Gt(ROSA)26Sortm9(CAGtdTomato)Hze* (called *R26R-TdTomato*; RRID:IMSR_JAX:007909, C57BL/6) were from The Jackson Laboratory. *Wnt1-Cre* were bred to *Bap1* on a mixed (C57BL/6J × CBA/J)F_1_ × C57BL/6 background to generate *Bap1^fl/fl^ Wnt1-Cre^+^* (called *Wnt1Bap1* KO). *Bap1* were bred to *Tyr-Cre* to generate *Bap1^fl/fl^ Tyr-Cre^+^* (called *TyrBap1* KO) and to *R26R-TdTomato* on a pure C57BL/6 background (called *TyrBap1 R26R-TdTomato*). For specific experiments, *TyrBap1 R26-TdTomato* were bred to *Chat-GFP* or *Hdac4* on a pure C57BL/6 background. *Ret-CreERT2* were bred to *R26R-TdTomato* and *Bap1* on a pure C57BL/6J background. Husbandry information is in Supplemental Table 1. Genotyping employed published and novel primers (Supplemental Table 2) and Transnetyx using tail or ear biopsies. Vaginal plug day was considered E0.5.

### Tamoxifen treatment of Bap1 Ret-CreERT2 TdTomato.

Tamoxifen (20 mg/mL; Sigma-Aldrich, catalog T5648) dissolved in 200 μL ethanol at 37°C was added to 1,800 μL sunflower oil (Sigma-Aldrich, catalog S5007). Adult *Bap1 Ret-CreERT2 TdTomato* or nursing dams (P1–P3) were gavaged (200 mg/kg tamoxifen) once daily on 4 days in a 5-day interval.

### Euthanasia.

P0–P7 mice were euthanized by decapitation, P15 by cervical dislocation, and adult mice with carbon dioxide (CO_2_, 3 minutes) followed by cervical dislocation.

### Whole-mount immunofluorescence.

Full-length bowel in 1× PBS (Thermo Fisher Scientific, catalog 21600069), opened along the mesenteric border, was pinned serosa-side-up to plates coated with Sylgard 184 Elastomer (Ellsworth Adhesives, catalog 184 SIL ELAST KIT 0.5KG) using insect pins (Fine Science Tools, catalog 26002-20) and fixed for 20 minutes at room temperature (RT) with 4% paraformaldehyde (Thermo Fisher Scientific, catalog O4042-500). After fixation, tissue was washed 3 times (5 minutes, 1× PBS) and stored in 50% glycerol/50% 1× PBS (Quality Biological, catalog A611-E404-99; Sigma-Aldrich, catalog S2002-25G) with 0.05% sodium azide (Sigma-Aldrich, catalog G9012-2L) at 4°C (<1 week) or –20°C (>1 week) until use. Before staining, stored tissues were rinsed 3 times (5 minutes, 1× PBS) and blocked (2 hours, RT; 5% normal donkey serum [Jackson ImmunoResearch Laboratories, catalog 017-000-121] and 0.1%–0.5% Triton X-100 [Sigma-Aldrich, catalogT8787] in PBS [0.1%–0.5% PBST]). HuC/D antibody (ANNA1) was incubated with tissue either 3 hours (RT) or overnight (4°C), then transferred after washing once in PBS (5 minutes, RT) into other primary antibodies (Supplemental Table 3) in 5% normal donkey serum (0.1%–0.5% PBST) and incubated either 3 hours (RT) or overnight (4°C). Tissue was then washed 3 times (30 minutes, 1× PBS), transferred into secondary antibody (Supplemental Table 3) in 5% normal donkey serum in 0.1%–0.5% PBST, incubated (RT, 60–90 minutes), washed 3 times (RT, 10–30 minutes, 1× PBS), and mounted in 50% glycerol/50% 1× PBS on slides (Thermo Fisher Scientific, catalog 1255015) using coverslips (Thermo Fisher Scientific, catalog 125485P). Incubation and wash steps were performed on a rocker (Cole-Parmer, catalog S2035-CP-A).

### EdU injection.

Mice were given 12.5 μg/g 5-ethynyl-2′-deoxyuridine (EdU) (Thermo Fisher Scientific, catalog C10337 and C10340) intraperitoneally in 1× PBS over 3 days, starting P4 (at 24-hour intervals). On P7, mice were euthanized 26 hours after the final EdU injection.

### Histochemistry for paraffin-embedded samples.

P15 small bowel or colon (0.7 cm segments from proximal and distal end of each region) was maximally stretched, pinned to Sylgard 184 Elastomer–coated dishes, fixed (20 minutes, RT, 4% paraformaldehyde), rinsed (1× PBS), stored in 70% ethanol (>3 hours, RT), and paraffin embedded. Longitudinal sections (5 μm thick) cut on an HM 355S Microm microtome were stained using a standard H&E protocol (Harris Modified Method Hematoxylin Stains, Thermo Fisher Scientific, catalog SH30-500D; Scott’s tap water, Sigma-Aldrich, catalog S5134; Eosin Y Solution, Sigma-Aldrich, catalog HT110116; mounting: Richard-Allan Scientific Cytoseal XYL, Thermo Fisher Scientific, catalog 8312-4).

For periodic acid–Schiff/Alcian blue (PAS/AB) staining, sections were incubated with Alcian blue (pH 2.5, RT, 6 minutes; Sigma-Aldrich, catalog A5268), washed (running tap water, 2 minutes), briefly rinsed (dH_2_O), treated with 0.5% periodic acid solution (RT, 5 minutes; Thermo Fisher Scientific, catalog A223-25), and again washed (dH_2_O), then incubated with Schiff’s reagent (RT, 15 minutes; Sigma-Aldrich, catalog 3952016), washed (running tap water, 5 minutes), stained with hematoxylin (45–60 seconds), washed again (running tap water, 2 minutes), differentiated with acid alcohol (1% concentrated HCl in 70% ethanol, 22 seconds), blued in Scott’s tap water (1 minute; Sigma-Aldrich, catalog S5134), and rinsed (running tap water) before dehydration and mounting (Richard-Allan Scientific Cytoseal XYL, Thermo Fisher Scientific, catalog 8312-4).

### Colon bead expulsion.

Adult mice (P95–P210) in empty cages, allowed to eat ad libitum before testing, were anesthetized (2 L/min carbogen, 2.5% [vol/vol] isoflurane, 1.5 minutes) before bead insertion (with 2–4 minutes of additional sedation if mice aroused before bead insertion). A glass bead (3 mm; Sigma-Aldrich, catalog Z143928) lubricated with sunflower seed oil was inserted 2 cm into colon using a custom-made 3-mm rounded glass rod. Anesthesia was discontinued. Time to bead expulsion was recorded. The assay was repeated 3 times per mouse with more than 48 hours between procedures. If bead insertion met resistance due to feces, mice were allowed to regain consciousness, and bead insertion was attempted again after 10 minutes. If resistance persisted, a new attempt was made 48 hours later.

### FITC-dextran small intestinal transit assay.

P15 mice gavaged with 70–100 μL FITC-dextran (10 mg/mL, MW 70,000; Sigma-Aldrich, catalog FD70S) in 2% methylcellulose (Sigma-Aldrich, catalog 274429) were kept in usual cages 90 minutes after gavage without their mother and without food or water. Small intestine from euthanized mice was cut into 12 equal-length segments. Colon was cut into 5 equal-length segments plus cecum. Segments minced with scissors in 400 μL 1× PBS were vortexed for 40 seconds to release FITC, then centrifuged (4,000*g*, 10 minutes). The fluorescence intensity of 100 microliters of supernatant was measured in a 96-well plate (Modulus II Microplate Multimode Reader (Turner BioSystems Inc.), excitation 490 nm, emission 510–570 nm). Data are presented as percentage fluorescence intensity (([fluorescence intensity in a bowel segment]/[sum of fluorescence intensities for all bowel segments]) × 100). Geometric center was calculated as:


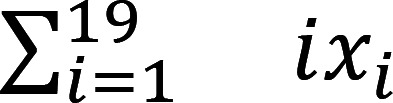


where *i* refers to each of the 19 bowel segments, ordered most proximal (stomach; segment 1) to most distal (distal colon; segment 19), and *x* refers to percentage fluorescence intensity in corresponding segments.

### In vivo analysis of bowel movement frequency and consistency.

P15 mice were separated into individual opaque containers without food around 8–9 am in a dark, undisturbed location. Welfare was confirmed every hour. Stool pellets in containers after 8 hours were counted. Presence of liquid diarrhea or feces-matted perianal fur was noted.

### In vitro organ bath experiments.

For P15 mice, full-length colon was immediately placed in warm (37°C) oxygenated (95% O_2_ and 5% CO_2_) Krebs-Ringer solution: 118 mM NaCl (Sigma-Aldrich, catalog S6191), 4.6 mM KCl (Thermo Fisher Scientific, catalog BP366-500), 2.5 mM CaCl_2_ (Sigma-Aldrich, catalog C-7902), 1.2 mM MgSO_4_ (Sigma-Aldrich, catalog M-7506), 1 mM NaH_2_PO_4_ (Sigma-Aldrich, catalog S0751), 25 mM NaHCO_3_ (Sigma-Aldrich, catalog BP328-500), 11 mM d-glucose (Sigma-Aldrich, catalog G7021; pH 7.4). To evacuate stool, Krebs-Ringer was pushed through bowel lumen with 21-gauge needles (BD Biosciences, catalog BD305167), touching epithelium as little as possible. After flushing, the entire colon was transferred to an organ bath (Hugo Sachs Elektronik–Harvard Apparatus, catalog D-79232) prefilled with warm, oxygenated Krebs-Ringer flowing through the chamber (9.2 mL/min). Colon was cannulated on both ends (Hugo Sachs Elektronik–Harvard Apparatus, catalog D-79232) and secured to cannulas with thread. Intraluminal pressure was created with a liquid reservoir connected to proximal colon maintained 2 cm above the surface of the water bath. Distal colon was connected to an outflow tube that opened 1 cm above the water bath surface to facilitate continuous flow of oxygenated Krebs-Ringer through colon.

For newborn (P0) mice, the most proximal 3 cm of small bowel was immediately placed in warm Krebs-Ringer and transferred to an organ bath (Hugo Sachs Elektronik–Harvard Apparatus, catalog D-79232) prefilled with warm (37°C), oxygenated Krebs-Ringer (95% O_2_ and 5% CO_2_) flowing through the chamber (9.2 mL/min). The proximal end of bowel was cannulated using a 10 μL pipette tip (Thermo Fisher Scientific, catalog 21-277-2A) and secured using string. Distal small bowel was pinned to Sylgard 184 Elastomer through the mesentery and not cannulated. Proximal-end cannula was connected to a 3 mL syringe via a 2-mm-outer-diameter plastic L-shaped tube, which maintained intraluminal pressure via a liquid reservoir 1 cm above the surface level of the water bath. Fluid in the liquid reservoir was allowed to freely flow from distal bowel, facilitating continuous flow of warm, oxygenated Krebs-Ringer through bowel.

P0 and P15 bowels were equilibrated 30 minutes before recording of a 20-minute video. Tetrodotoxin (Abcam, catalog ab120054; 1 mM stock in sodium citrate buffer consisting of 40 mM citric acid monohydrate [EMD Millipore, catalog CX1725-1] and 60 mM trisodium citrate dihydrate [Sigma-Aldrich, catalog C3434], pH 4.8) was then added to warm oxygenated Krebs-Ringer at 1 μM (final concentration). An additional video was recorded for 20 minutes immediately after tetrodotoxin application.

### Video imaging and data processing.

An E-PM1 Olympus digital camera was mounted on a dissecting microscope (Zeiss Stemi 305 CAM Digital Stereo Zoom Microscope, ×0.8–4.0) to image bowel at 15 frames per second, resolution 1,920 × 1,080 pixels. Organ bath was illuminated using a dissecting microscope light source. Contrast was provided by the securing of black paper to the chamber bottom (P15 bowel) or below Sylgard Elastomer (P0 bowel). Video files were converted from.MTS to.mp4 using VLC Media Player. Spatiotemporal information was converted to matrices using a custom MATLAB script (https://github.com/christinawright100/BowelSegmentation/commit/095850905504f6ac29c7311f56fd9bf089ad4574, commit ID 958509). The script first thresholds images, then separates and distinguishes bowel from background. Thresholded images were used to determine bowel width for full length of imaged bowel for duration of videos and to create kymographs depicting bowel width over time for entire imaged bowel regions. Colonic motor complexes (CMCs), defined as repetitive neurogenic contractions containing a defined peak point of contraction, were scored in a blinded fashion.

### Microscopy.

Whole-mount immunofluorescence images were acquired with a Zeiss LSM 710 confocal microscope with a ×20/0.8 air or ×63/1.4 oil DIC M27 Plan-Apochromat objective and Zeiss Zen 2.3 SP1 FP3 (Black) LSM710 Release Version 14.0.0.0. Images of H&E- or PAS/AB–stained tissue were acquired with a Leica DMC 2900 camera attached to a Leica DM 6000B epifluorescence microscope with ×20/0.7 air HC Plan-Apochromat objective and Leica Application Suite X version 2.0.0.14332.2 (Leica Microsystems). Confocal images show either single optical slices or maximum-intensity *Z*-projections (see figure legends). ImageJ (NIH) was used to uniformly color-adjust images. ImageJ or Inkscape (http://www.inkscape.org/) was used to crop images.

### Biological sample size and technical replicates.

For H&E and PAS/AB immunohistochemistry, 5 sections separated by at least 200 μm were imaged for each bowel region in each mouse. For proximal small intestine and distal colon, only fields containing at least one “intact” villus and/or crypt were imaged. For distal small intestine and proximal colon, entire sections were imaged and analyzed.

For whole-mount immunofluorescence at P0 and P15, at least 4 randomly chosen full-thickness confocal *Z*-stacks obtained with a ×20 objective were analyzed as technical replicates for each individual bowel region. For P5 and P15 ENS quantifications, cells in the entire field of view (180,625 μm^2^) were counted. For P0 ENS quantifications, cells in 2 randomly chosen quadrants of imaged fields of view (90,312.5 μm^2^) were counted. Alternative quadrants were randomly selected for analysis if tissue appeared damaged. For P0 *TyrBap1* ENS quantification only, at least 4 randomly chosen full-thickness confocal *Z*-stacks obtained with a ×63 objective were quantified as technical replicates for each individual bowel region.

### Whole-cell enteric neuron isolation from P5 mouse colon.

P5 *TyrBap1 R26R-TdTomato* and *Bap1-wt/wt Tyr-Cre R26-TdTomato* pups (Supplemental Table 4) were euthanized and rapidly dissected on ice. Full-length colons were opened at the mesenteric border, and muscularis was removed using fine forceps. For each pup, muscularis in carboxygenated (5% CO_2_/95% O_2_) 1× Hanks balanced salt solution (HBSS; Thermo Fisher Scientific, catalog 14025092) was cut into small pieces with insulin needles and dissociated with Liberase (0.625 mg/mL; Sigma-Aldrich, catalog 5401135001) plus DNase I (0.1 mg/mL; Sigma-Aldrich, catalog 11284932001) and MgCl_2_ (6 mM; Thermo Fisher Scientific catalog BP214-500) in DMEM/F-12 (Thermo Fisher Scientific, catalog 11320033) for 40 minutes at 37°C. Cells were triturated with a P1000 pipette tip every 10–15 minutes during Liberase digestion. Dissociated cells were passed through Falcon 35-μm filters (Corning, catalog 352235), pelleted (150*g*, 5 minutes, 4°C) after addition of 0.04% wt/vol BSA (Thermo Fisher Scientific, catalog AM2618) in HBSS (1/3 final volume), and then resuspended in 1% wt/vol BSA (Thermo Fisher Scientific, catalog AM2618) in HBSS. After an additional pass through Falcon 35-μm filters, the cells were sorted (BD FACSJazz, BD Biosciences; 100 μm nozzle) into 300-μL 0.04% BSA in HBSS. Sorted cells were pelleted (150*g*, 5 minutes, 4°C). Supernatant was removed, leaving only about 30 μL that was submitted for scRNA-Seq.

### Library generation, sequencing, and data processing.

Libraries prepared with Chromium Single Cell 3′ Reagent Kits v3 (10x Genomics) were sequenced on an Illumina NovaSeq 6000 system. Cell Ranger pipeline (10x Genomics, v3.1.0) was used to convert BCL into FASTQ files, perform STAR alignment (53) to mm10 genome, filter, count unique molecular identifiers (UMIs), and generate gene-barcode matrices.

### Single-cell RNA-Seq analysis.

Using Seurat version 3.1.2 (54, 55), gene-barcode matrices were imported into R (version 3.6.2), filtered to remove low expressors or doublets (nGene &gt, 1,500; UMI &lt, 50,000) and mitochondrial contaminants (percent mitochondrial RNA >10%), normalized, and scaled to regress out variance due to differing percent mitochondrial RNA per individual cell (SCTransform function) (WT data set: 9,128.41 ± 6,058.95 mean ± SD number of UMIs and 3,105.59 ± 1,008.59 mean ± SD number of unique genes; KO data set: 9,821.48 ± 8,079.56 mean ± SD number of UMIs and 3,054.88 ± 1,228.31 mean ± SD number of unique genes). Cells for each data set (WT and KO data sets) were separately clustered setting resolution to 0.8 and using the most statistically significant principal components up to the number at which additional principal components contributed <5% of standard deviation and principal components cumulatively contributed 90% of SD, or at which variation changed by <0.1% between consecutive principal components (48) (13 principal components for KO data set and 16 principal components for WT data set). After uniform manifold approximation and projection (UMAP) clustering, neuron cell clusters were identified as high expressors of pan-neuronal markers *Elavl14* and *Tubb3* but with low or absent expression of enteric glial markers *Plp1* and *Sox10*, and low expression of markers for myenteric plexus–associated cell types *Actg2* (visceral smooth muscle), *Kit* (interstitial cells of Cajal), and *Pdgfra* (PDGFRα cells). Non-neuronal cell types were removed from analysis, and data sets were renormalized and rescaled to regress out variance due to differing percent mitochondrial RNA per individual cell (SCTransform function). Two separate neuron data sets were then integrated in their normalized and scaled forms (IntegrateData function) followed by normalization (Seurat default natural log-transformed RP10k normalization) and scaling to regress out variance due to differing percent mitochondrial RNA per individual cell. Fifteen principal components were determined via the method described above, and cells were clustered using resolution 0.6. The FindAllMarkers function (assay “RNA”) was used to compare gene expression across neuron clusters in integrated data sets. Gene expression was also compared across all neurons derived from original WT versus KO data sets as well as WT and KO data set–derived neurons within each individual neuron cluster in combined data sets. For all gene expression analyses, only genes expressed by more than 10% of cells in a given cluster were included. Genes enriched by more than 0.25 ln(fold change of mean expression level) compared with cells in all other clusters were considered differentially expressed. For RNA velocity analysis performed on WT and KO data sets, velocyto version 0.17.17 (run command) (56) was used to quantify spliced and unspliced transcripts from position-sorted aligned and unaligned reads and filtered_feature_bc_matrix from Cell Ranger. Spliced and unspliced counts were merged in Python version 3.10.10 (anndata version 0.9.1, ref. 57; and scanpy version 1.9.3, ref. 58) with clustering metadata and UMAP coordinates from integrated Seurat object (R version 4.3.1 and Seurat version 4.3.0.1) of neurons and glia. scVelo version 0.2.5 (59) was run using the dynamical model, and velocities were projected onto UMAP embedding.

### Statistics.

We used Prism 7.03–9.4.1 (GraphPad Software) for statistical analysis. All images or videos were blinded prior to analyses. *Bap1*-mutant mice were always compared with *Bap1* controls within the same background strain. *P* values less than 0.05 were considered significant. All data were tested for normality using the Shapiro-Wilk normality test. Appropriate tests were chosen as detailed in the figure legends and Supplemental Table 4.

### Study approval.

Studies adhered to ARRIVE (Animal Research: Reporting of In Vivo Experiments) guidelines. Mouse experiments were performed in accordance with Institutional Animal Care and Use Committee (IACUC) approval from the Children’s Hospital of Philadelphia (IACUC#19-001041) and the University of Miami Miller School of Medicine (Miami, Florida, USA) (IACUC#21-180).

### Data availability.

Raw and processed single-cell RNA sequencing data are available at the NCBI’s Gene Expression Omnibus database (GEO GSE242001). Values for all data in graphs are reported in the Supporting Data Values file. Code for all analyses is available on GitHub at github.com/HeuckerothLab/Bap1_Schneider2024/. Processed data files are available on the Open Science Framework at https://osf.io/jgnve/

## Author contributions

SS and ROH conceived the study and designed the project. SS, JBA, RPB, KB, CMW, and ROH developed methodology. SS, JBA, RPB, KB, and ROH pursued formal data analysis. SS, JBA, RPB, KB, GY, and BAM generated data. DMT, JWH, and ROH provided resources. SS, JBA, KB, and ROH wrote the original draft. All authors (SS, JBA, RPB, KB, CMW, BAM, GY, DMT, JWH, and ROH) reviewed and edited the final manuscript. ROH, DMT, and JWH provided funding. ROH supervised all aspects of this work.

## Supplementary Material

Supplemental data

Supporting data values

## Version 1 05/01/2024

Electronic publication
