# Supplementary material for: BAP1 is required prenatally for differentiation and maintenance of postnatal murine enteric nervous system
Source: J Clin Invest. 2024 Mar 12;134(9):e177771. doi: 10.1172/JCI177771 (PMC11060734; doi:10.1172/JCI177771)
Supplement: Supplemental data [file jci-134-177771-s067.pdf]

Supplemental Methods

Pathway Analysis

MSigDB Overlap Analysis was performed using MSigDB's online tool (https://www.gsea-msigdb.org/gsea/msigdb/mouse/annotate.jsp). Overlap analysis was run using the Hallmark ("MH") and Canonical Pathways ("M2:C2:CP") gene sets (Mouse MSigDB v2023.2.Mm) (1-4). For each cell type (neurons and glia), top genes (by greatest log2FC, maximum of 200 genes) more abundant in WT and top genes more abundant in *TyrBap1* KO were used as the input gene lists. Tables of results were downloaded and visualized as dotplots in R using ggplot2 (5).

Gene set enrichment analysis was performed using fgsea (version 3.17) (6). All genes from the differential expression analysis were used for fgsea analysis, with log2 fold change as the ranking metric and a minimum pathway size of 3. The MSigDB pathway gene sets Hallmark (H) and Canonical Pathways (CP) for mouse (MSigDB v7.5.1) used for pathway analysis in fgsea were accessed using msigdbr (v7.5.1) (7).

Glial Single Cell RNA Expression Analysis

Glial cells were defined as all non-neuronal cell types remaining in the KO and WT datasets after removal of neuronal and myenteric-plexus associated cell types as described in the "Single cell RNAseq Analysis" methods. The data sets were re-normalized and re-scaled to regress out variance due to differing percent mitochondrial RNA per individual cell (SCTransform function). WT and KO datasets were separately clustered with resolution to 0.6 and 15 and 13 principal components for KO and WT datasets, respectively. The number of principal components was determined in the same method as described in the "Single cell RNAseq Analysis" section of main text methods. The two separate neuron datasets were then integrated in their normalized and scaled forms (IntegrateData function) followed by normalization (Seurat default natural log-transformed RP10k normalization), and scaling to regress out variance due to differing percent

mitochondrial RNA per individual cell. 15 principal components were determined in the same method as described in the "Single cell RNAseq Analysis" section of main text methods and cells were clustered using resolution 0.6. The "FindAllMarkers" function (assay = "RNA") was used to compare gene expression across glia clusters. Gene expression was also compared across all glia derived from the original WT versus KO datasets as well as WT and KO dataset-derived glia within each individual neuron cluster in the combined dataset. For all gene expression analyses, only genes expressed by >10% of cells in a given cluster were included. Genes enriched by >0.25 ln(fold change of mean expression level) compared with cells in all other clusters were considered differentially expressed.

Supplemental Figures

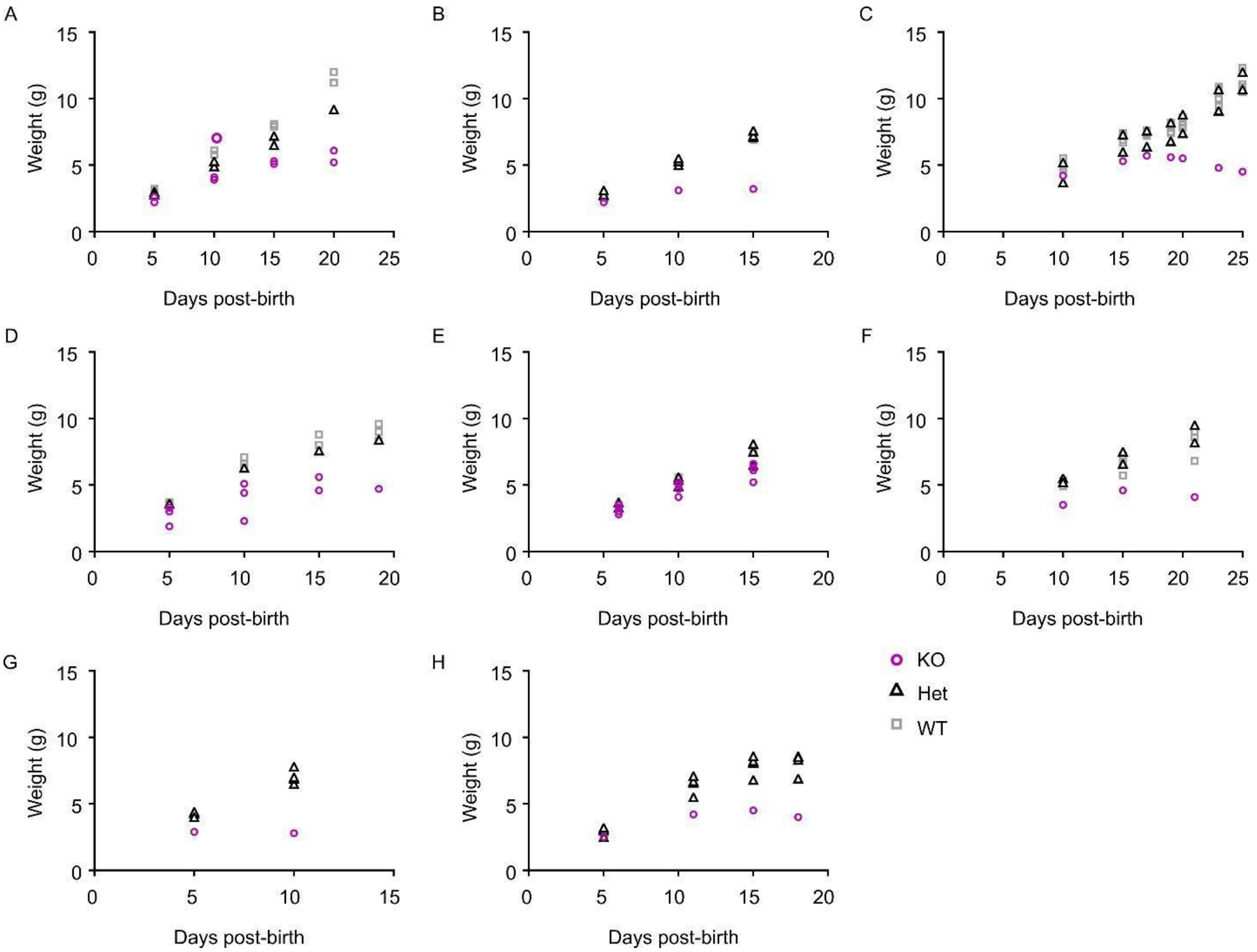

Supplemental Figure 1: The failure to thrive phenotype for individual *TyrBap1* KO mice becomes apparent at different postnatal ages.

(A-H) Weights for individual pups in early postnatal life. Each graph includes all pups in a single litter. *TyrBap1* KO mice gain weight more slowly beginning between P5 and P15, then lose weight and die early. KO = *TyrBap1* KO. Het =*Bap1*$^{fl/wt}$; *Tyr-Cre*$^{+}$ WT = *Bap1*$^{wt/wt}$;*Tyr-Cre*$^{+}$.

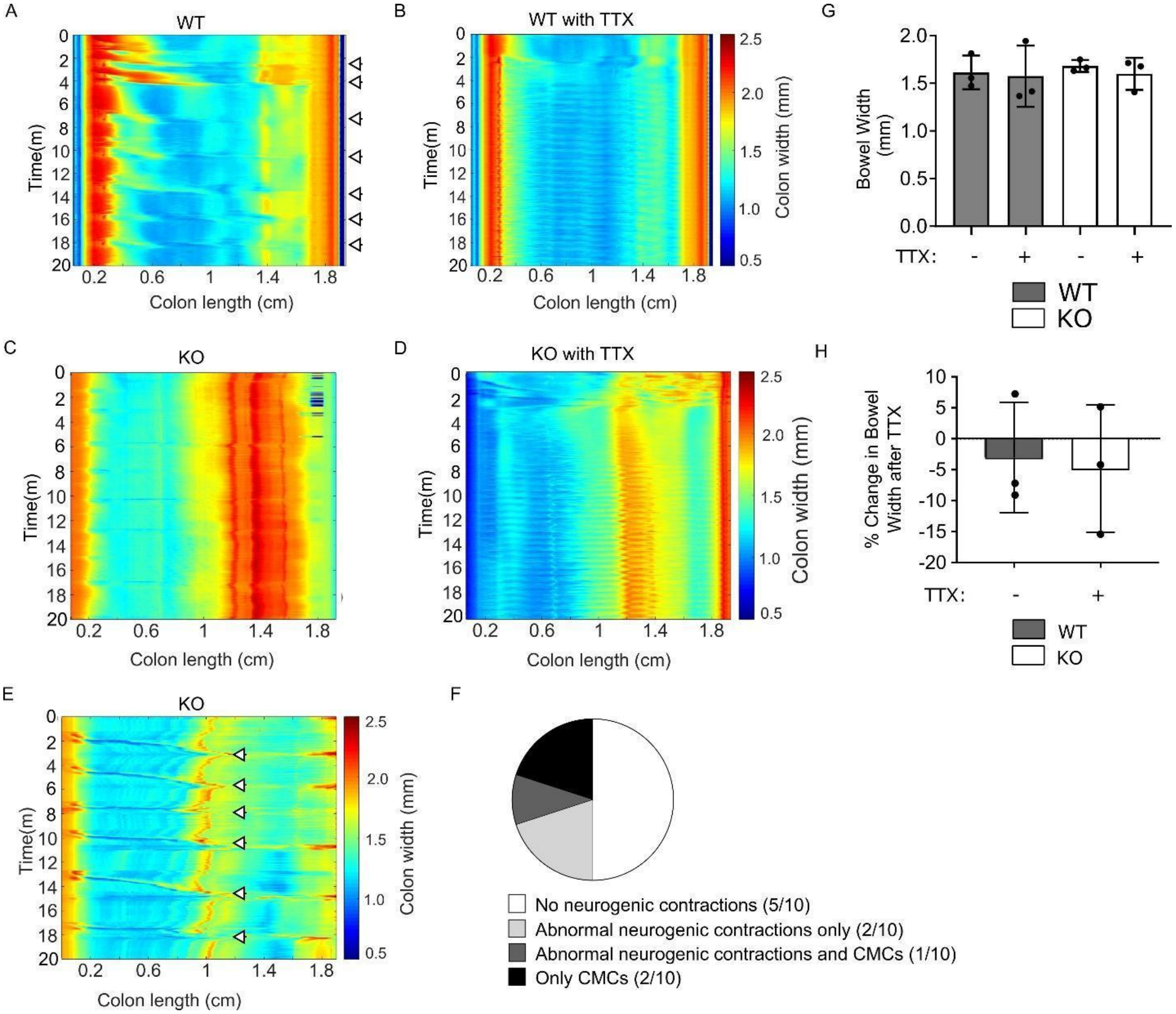

Supplemental Figure 2: Nervous system mediated motility patterns were often abnormal or absent in *TyrBap1* KO mice at P15.

(A-D) Representative kymographs depict colon width in an oxygenated organ bath as a function of time and distance along the proximo-distal axis for WT (*Bap1*$^{wt/wt}$*; Tyr-Cre*$^{+}$; A, B) and *TyrBap1* KO (C, D) mice at P15. (A, B) Colonic motor complexes (CMC) (A; white arrows) disappeared in the presence of tetrodotoxin (TTX, B). (C, D) For some P15 *TyrBap1* KO mice, no CMC was recorded (C) and this was also true in the presence of TTX (D). (E) Example kymograph for a P15 *TyrBap1* KO mouse with regular but abnormal neurogenic propagating motility patterns. (F) Contraction patterns observed for P15 *TyrBap1* KO mice (n=10). (G) Colon width did not change

significantly in the presence of TTX for either P15 WT or *TyrBap1* KO mice. (H) Percent change in colon width after TTX as compared to baseline also did not differ significantly for P15 WT and *TyrBap1* KO mice. (F-G) Graphs show mean ± SD. Data not significant unless otherwise indicated. (G) Brown-Forsythe ANOVA test. (H) Unpaired T-test with Welch's correction.

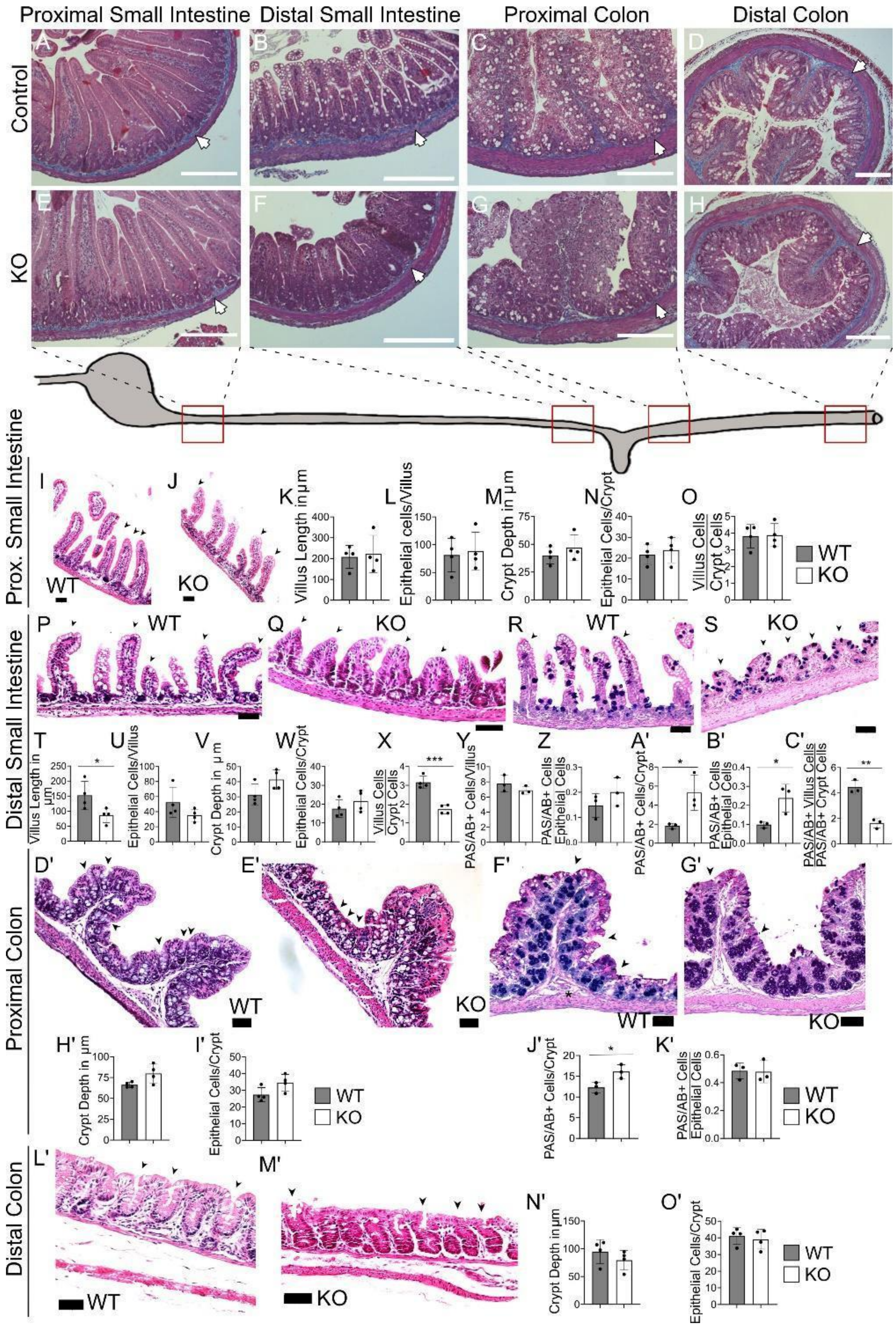
Proximal Small Intestine
Distal Small Intestine
Proximal Colon
Distal Colon
Control
KO
Prox. Small Intestine
Villus Length in μm
Epithelial cells/Villus
Crypt Depth in μm
Epithelial Cells/Crypt
Villus Cells / Crypt Cells
WT
KO
Distal Small Intestine
PAS/AB+ Cells/Villus
PAS/AB+ Cells / Epithelial Cells
PAS/AB+ Cells/Crypt
PAS/AB+ Villus Cells / PAS/AB+ Crypt Cells
Proximal Colon
Distal Colon

Supplemental Figure 3: *TyrBap1* KO mice at P15 had altered bowel epithelium.

(A-H) Trichrome stained bowel had similar abundance of collagen in control (A-D) and *TyrBap1* KO (E-H) proximal small intestine (A, E), distal small intestine (B, F), proximal colon (C, G), and distal colon (D, H). White arrows indicate trichrome stained collagen in submucosa (blue). Representative images shown. (I, J) Representative images of hematoxylin and eosin (H&E) - stained proximal small intestine of WT (I) and *TyrBap1* KO (J) mice. Black arrowheads indicate villi that were included in the quantification. (K-O) WT and *TyrBap1* KO had similar proximal small intestine villus length (K), number of epithelial cells per villus (L), crypt length (M), number of epithelial cells per crypt (N), and ratio of villus epithelial cells to crypt epithelial cells (O). (P-Q) Representative images of H&E-stained distal small intestine of WT (P) and *TyrBap1* KO (Q) mice. Black arrowheads highlight villi included in the quantification. (R, S) Alcian Blue/Periodic Acid-Schiff (AB/PAS) staining was used to visualize goblet cells (dark purple spots within epithelium) in distal small intestine of WT (R) and *TyrBap1* KO (S) mice. Black arrowheads highlight villi included in quantification. (T) *TyrBap1* KO mice had shorter distal small intestine villi compared to WT mice. (U-W) WT and *TyrBap1* KO had similar number of epithelial cells per villus (U), crypt depth (V), and the number of epithelial cells per crypt (W) in distal small intestine. (X) Ratio of villus epithelial cells to crypt epithelial cells was lower in *TyrBap1* KO compared to WT distal small intestine consistent with (T). (Y, Z) WT and *TyrBap1* KO had similar numbers of goblet cells per villus (Y) or per villus epithelial cell numbers (Z). (A', B') *TyrBap1* KO distal small intestine crypts contain significantly greater numbers of goblet cells per crypt compared to WT (A') with an increased goblet cell to epithelial cell ratio (B'). (C') A reduced ratio of villus PAS+ goblet cells to crypt PAS+ goblet cells in *TyrBap1* KO is consistent with increased PAS+ goblet cell numbers in *TyrBap1* KO distal small intestine crypts compared to WT (A'). (D'-E') Representative hematoxylin and eosin-stained proximal colon of WT (D') and *TyrBap1* KO (E') mice. Black arrowheads highlight crypts included in the quantification. (F', G') AB/PAS staining visualized goblet cells (dark

purple spots within epithelium) in the proximal colon of WT (F’) and *TyrBap1* KO (G’) mice. Black arrowheads highlight crypts included in the quantification. (H’, I’) *TyrBap1* KO and WT had similar proximal colon epithelial crypt depth (H’) and similar proximal colon epithelial cells per crypt (I’). (J’) *TyrBap1* KO mice had significantly greater numbers of PAS+ goblet cells in proximal colon crypts compared to WT mice. (K’) However, this increase in PAS+ goblet cell number disappeared when normalized to the number of epithelial cells per crypt. (L’, M’) Representative images of hematoxylin and eosin-stained distal colon of WT (L’) and *TyrBap1* KO (M’) mice. Black arrowheads highlight crypts included in the quantification. (N’, O’) *TyrBap1* KO and WT had similar distal colon crypt depth (N’) and number of epithelial cells per crypt (O’). (A-D) Control refers to $Bap1^{wt/wt}$;*Tyr-Cre*$^{+}$ genotype or $Bap1^{fl/wt}$; *Tyr-Cre*$^{+}$. (I-O’) WT refers to $Bap1^{wt/wt}$;*Tyr-Cre*$^{+}$. Data show mean ± SD. *p<0.05. (A-H) Scale bar = 200 µm. (I-M’) Scale bar = 50 µm. (K-O, T-X, Y, Z, A’, B', C', H’, I’, J’, K’, N’, O’) Unpaired two-tailed t-test.

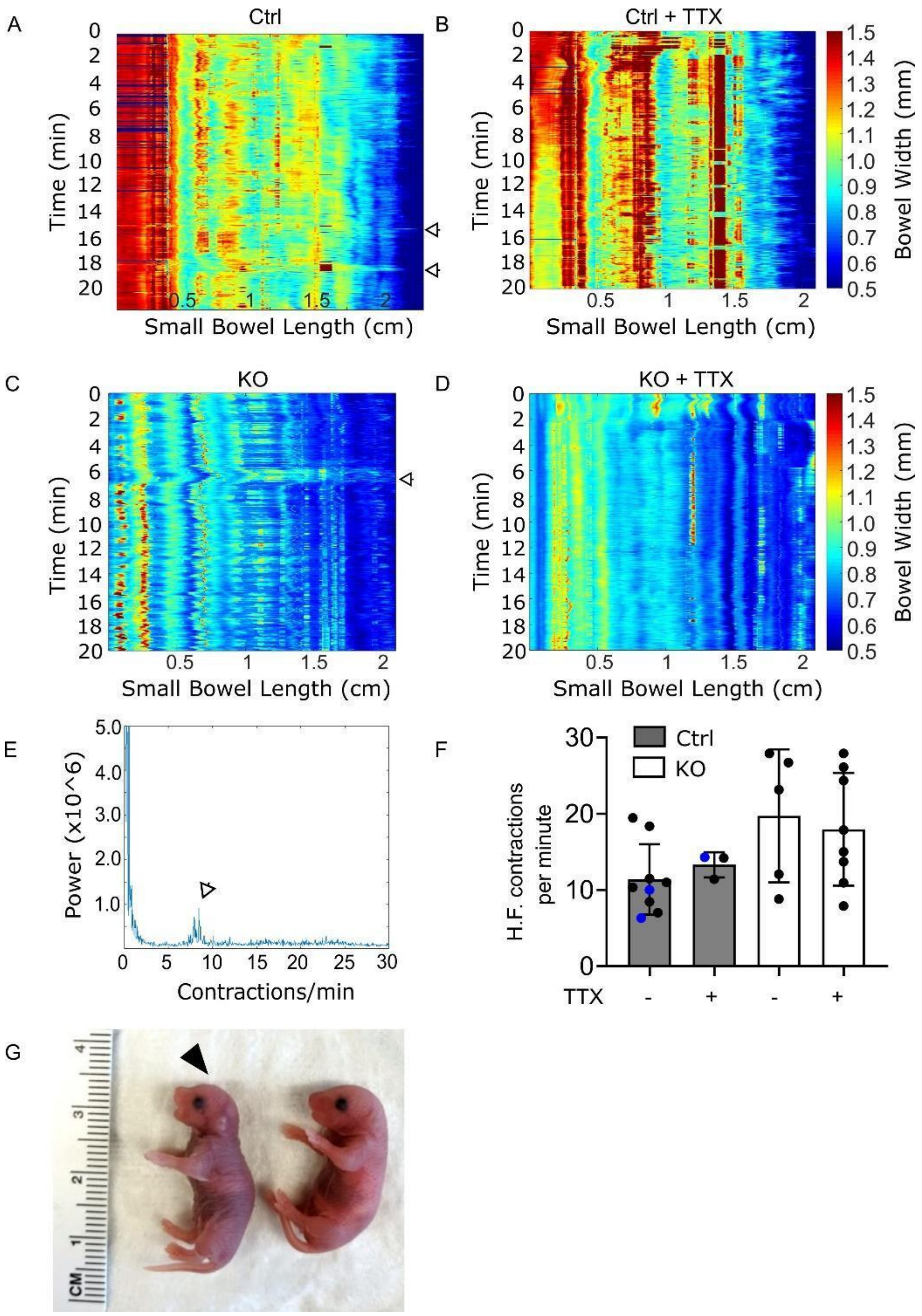

Supplemental Figure 4: Neurogenic and myogenic neonatal proximal small intestine motility patterns *ex vivo* appear similar in *Wnt1Bap1* KO and control mice at birth.

(A-D) Representative kymographs depicting proximal small bowel width as a function of time and distance along the proximo-distal axis of P0 control (Ctrl; A, B) and *Wnt1Bap1* KO (C, D) mice in an oxygenated organ bath. (A, B) Low frequency (L.F.) neurogenic contractions could be recorded

for a subset of control mice (A; white arrows). These L.F. contractions disappeared in the presence of tetrodotoxin (TTX, B). (C, D) L.F. contractions could also be recorded for a subset of *Wnt1Bap1* KO mice (C; white arrows). These L.F. contractions also disappeared in the presence of tetrodotoxin (TTX, D). (E) Sample Fourier transform shows the frequency spectrum of regularly occurring myogenic higher frequency contractions (H.F. contractions; white arrow). (F) P0 *Wnt1Bap1* KO and control had similar myogenic H.F. contractions frequency. The H.F. contractions frequency was unaffected by tetrodotoxin as expected for myogenic contractions. Data points derived from Het pups are marked in blue. (G) *Wnt1Bap1* KO pup at postnatal day 0 (black arrowhead) gasping for air and cyanotic next to control littermate. (A-F) WT refers to *Bap1*$^{wt/wt}$*; Wnt1-Cre*$^{+}$ or any genotype without the *Wnt1-Cre* transgene. Het refers to *Bap1*$^{fl/wt}$*; Wnt1-Cre*$^{+}$. KO refers to *Wnt1Bap1* KO. (F) Data show mean ± SD. Data not significant unless otherwise indicated. (F) Brown-Forsythe ANOVA test.

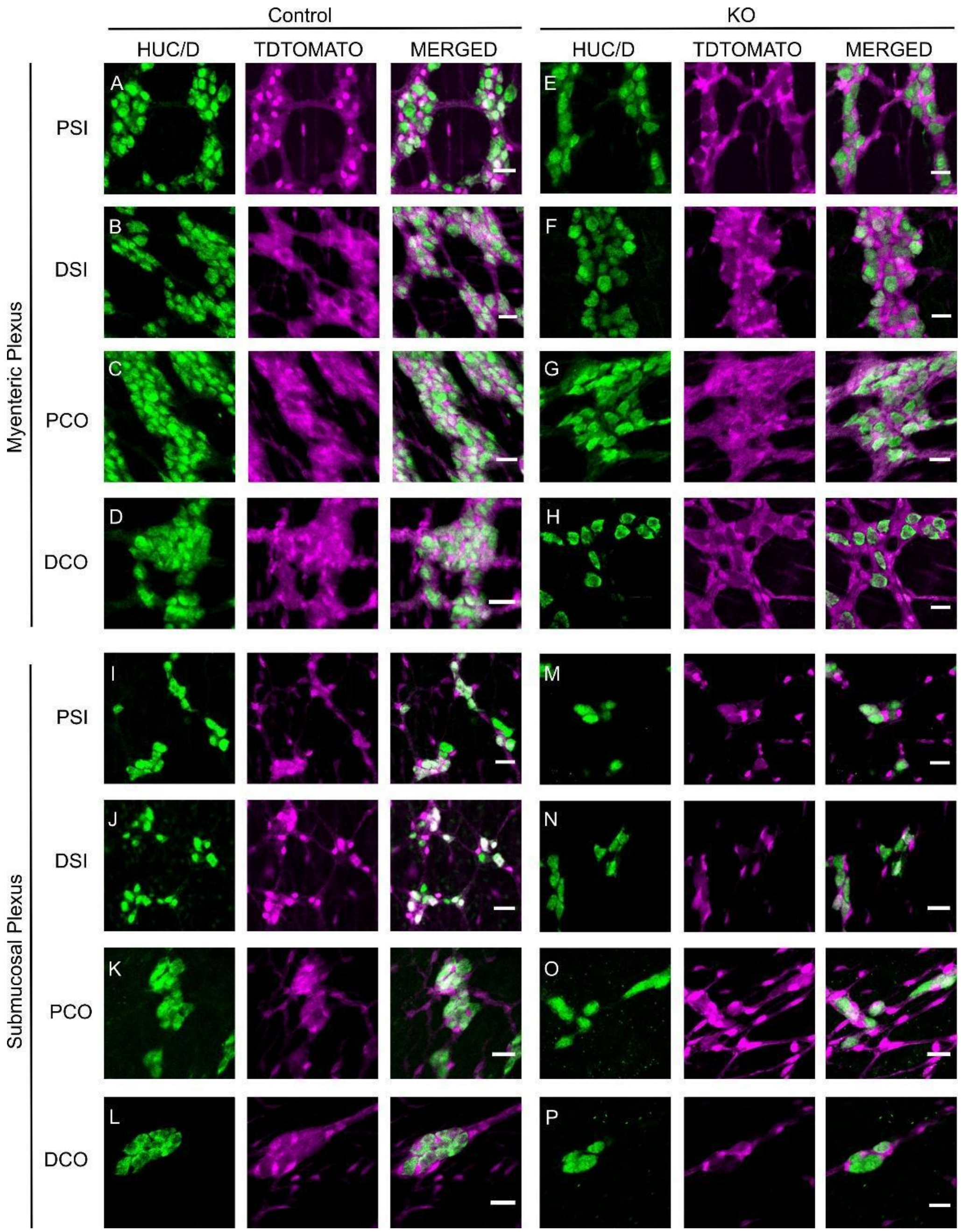

Supplemental Figure 5: Tyr*Bap1* KO mice have reduced numbers of *Tyr-Cre* lineage neurons by postnatal day 15.

(A-P) Representative images of myenteric (A–H) and submucosal plexus (I-P) in various bowel regions for *Bap1*$^{wt/wt}$*; Tyr-Cre*$^{+}$ (Control; A-D, I-L) and *TyrBap1* KO (KO; E-H, M-P) mice at P15. Purple = TDTOMATO (*Tyr-Cre* lineage neurons), green = HuC/D (neurons). (E, K, M, O)

Maximum intensity Z-projections. All other images are single confocal optical slices. PSI= proximal small intestine, DSI = distal small intestine, PCO = proximal colon, and DCO = distal colon. Scale bar = 25 μm.

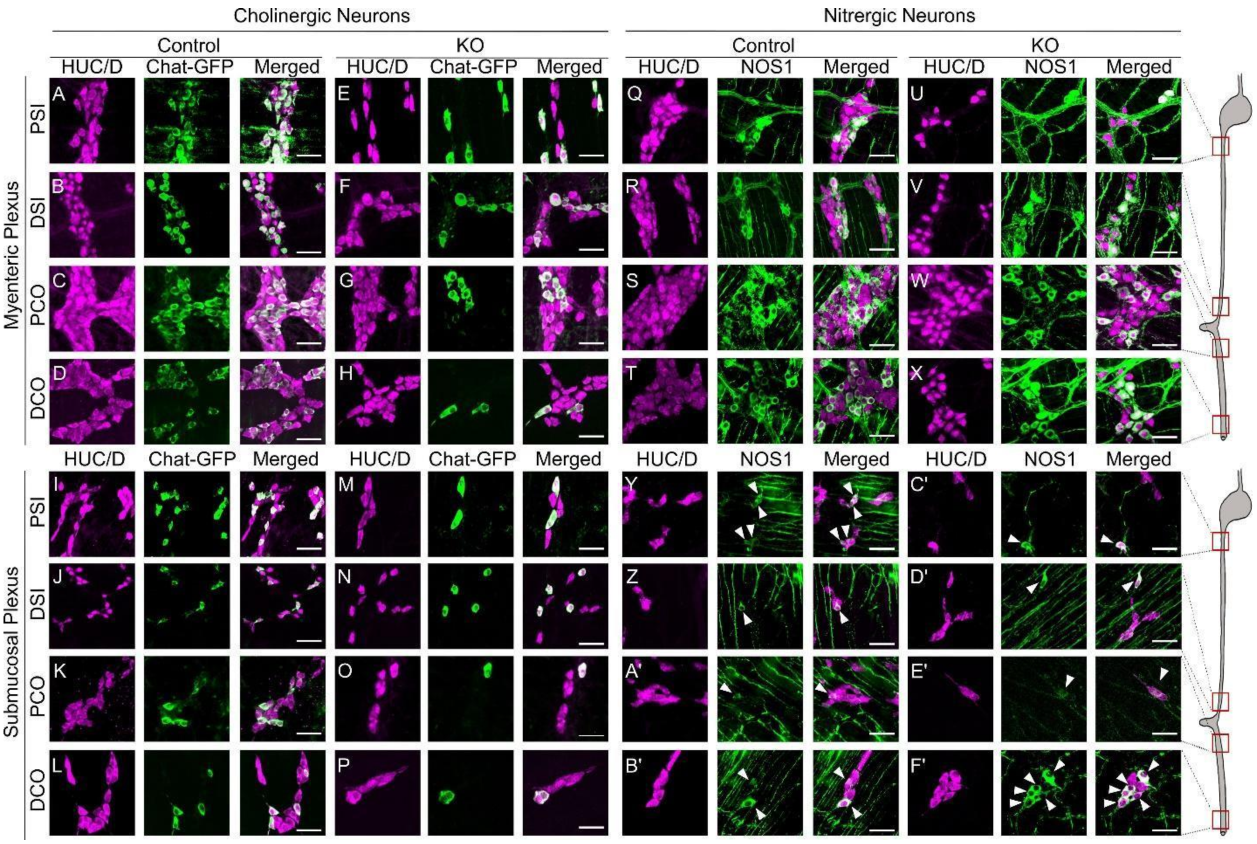

Supplemental Figure 6: *TyrBap1* KO mice at P15 had decreased total enteric neuron density in all bowel regions examined with disproportionate loss of Chat-GFP+ neurons and proportionate loss of nitrergic enteric neurons.

(A-P) Representative images of cholinergic neurons in the myenteric plexus (A–H) and submucosal plexus (I-P) in various bowel regions of *Bap1*$^{wt/wt}$*; Tyr-Cre*$^{+}$ (Control; A-D, I-L) and *TyrBap1* KO (KO; E-H, M-P) mice at P15. Purple = HUC/D (neurons), green = GFP (cholinergic neurons identified based on Chat-GFP expression). (Q-F') Representative images of nitrergic neurons in the myenteric plexus (Q-X) and sample images of nitrergic neurons in the submucosal plexus (Y-F') along the bowel for *Bap1*$^{wt/wt}$*; Tyr-Cre*$^{+}$ (Control; Q-T,Y-B') and *TyrBap1* KO (KO; U-X,C'-F') mice at P15. Submucosal plexus images are not representative due to low absolute nitrergic neuron count and great variability in nitrergic neuron density. Purple = HuC/D (neurons), green = NOS1 (nitrergic neurons). (A-F') Maximum intensity Z-projections. PSI = proximal small

intestine, DSI = distal small intestine, PCO = proximal colon, DCO = distal colon. Scale bar = 50 μm.

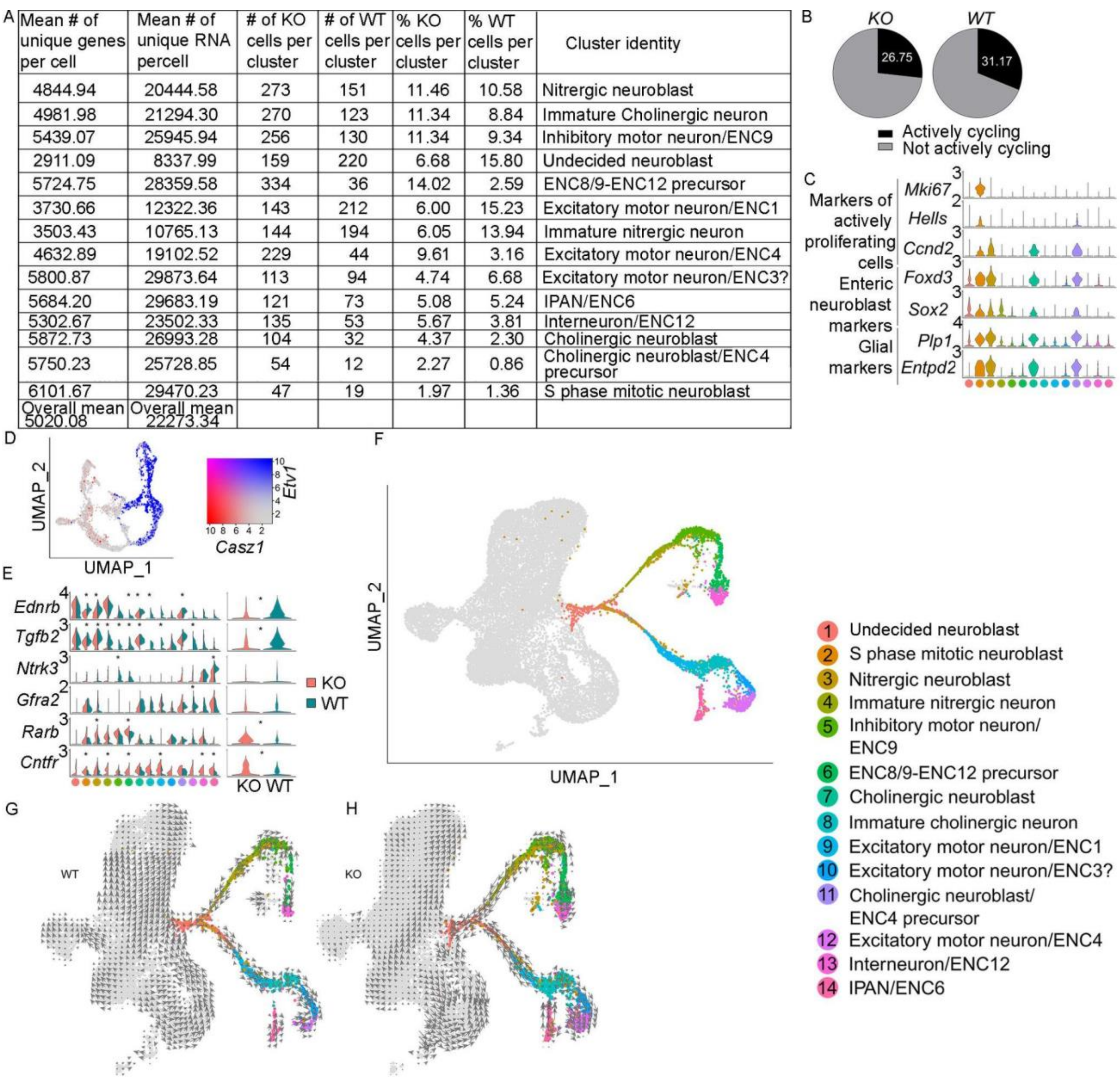

Mean # of unique genes per cell	Mean # of unique RNA percell	# of KO cells per cluster	# of WT cells per cluster	% KO cells per cluster	% WT cells per cluster	Cluster identity
4844.94	20444.58	273	151	11.46	10.58	Nitrergic neuroblast
4981.98	21294.30	270	123	11.34	8.84	Immature Cholinergic neuron
5439.07	25945.94	256	130	11.34	9.34	Inhibitory motor neuron/ENC9
2911.09	8337.99	159	220	6.68	15.80	Undecided neuroblast
5724.75	28359.58	334	36	14.02	2.59	ENC8/9-ENC12 precursor
3730.66	12322.36	143	212	6.00	15.23	Excitatory motor neuron/ENC1
3503.43	10765.13	144	194	6.05	13.94	Immature nitrergic neuron
4632.89	19102.52	229	44	9.61	3.16	Excitatory motor neuron/ENC4
5800.87	29873.64	113	94	4.74	6.68	Excitatory motor neuron/ENC3?
5684.20	29683.19	121	73	5.08	5.24	IPAN/ENC6
5302.67	23502.33	135	53	5.67	3.81	Interneuron/ENC12
5872.73	26993.28	104	32	4.37	2.30	Cholinergic neuroblast
5750.23	25728.85	54	12	2.27	0.86	Cholinergic neuroblast/ENC4 precursor
6101.67	29470.23	47	19	1.97	1.36	S phase mitotic neuroblast
Overall mean 5020.08	Overall mean 22273.34					

Supplemental Figure 7: Reduced number of proliferating neuroblasts and abnormal differentiation of a subset of enteric neuron subtypes at P5 in *Tyr-Cre* lineage neurons of *TyrBap1* KO mice.

Descriptive statistics of single cell RNA seq neuron dataset derived from *TyrBap1* KO (KO) versus *Bap1*$^{wt/wt}$; *Tyr-Cre*$^{+}$ (WT) tissue. (B) Percentage of total WT (right) or *TyrBap1* KO (left) cells in neuron dataset with gene expression patterns indicating cell division. (C) Violin plots show relative abundance of selected proliferation markers, enteric neuroblasts markers, and enteric glial

markers expressed in early neuroblasts. (D) FeaturePlot showing expression of cholinergic neuron-specific transcription factor *Casz1* (red) and nitrergic neuron transcription factor *Etv1* (blue) across the neuron dataset. (E) Violin plots show relative abundance of differentially expressed growth factor receptors and the stem cell niche growth factor *Tgfb2* in *Bap1*$^{wt/wt}$*;Tyr-Cre*$^{+}$ (WT, blue color) and *TyrBap1* KO (KO, red color) neurons for each cluster (left side) and all WT versus KO neurons (right side). (F) UMAP projection of enteric neuron subtypes and glia in P5 colon myenteric plexus using unsupervised clustering of integrated *TyrBap1* KO and WT cells. Each dot on the plot represents a single cell and the color indicates the neuron subtype (cluster) identity. Gray dots represent enteric glia. (G, H) RNA velocity vector field superimposed onto the UMAP in Figure 7F suggesting the direction of colonic myenteric neuron differentiation (indicated by arrow direction) at P5 for WT (G) and *TyrBap1* KO (H) neurons and glia. (C-F) Data shows ln(normalized & scaled expression level) where mean expression level of each gene of interest across all cells in the dataset is defined as ln(1).

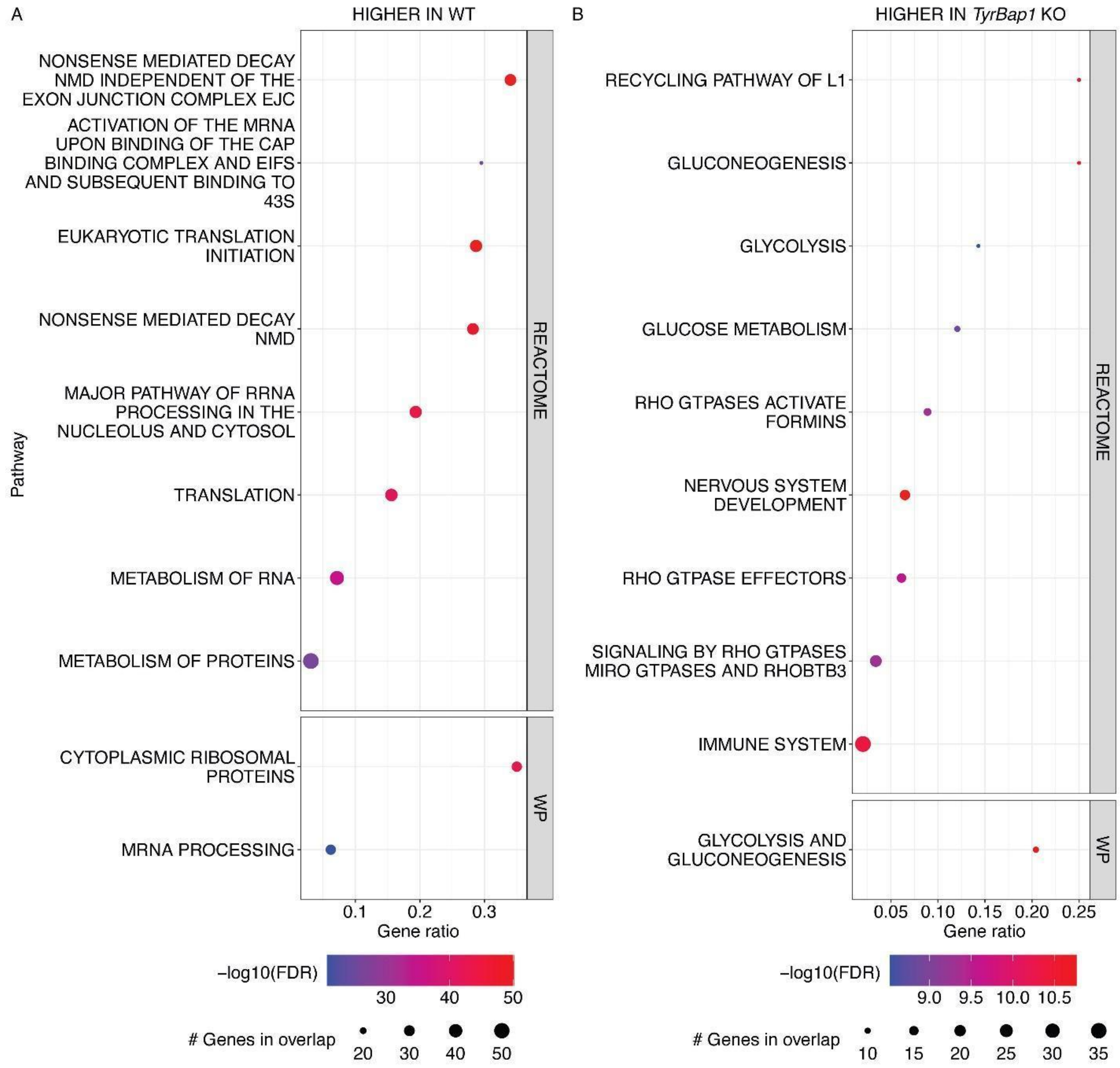

Supplemental Figure 8. Pathway analysis of differentially expressed genes (pval<0.05) in enteric neurons.

Dotplots show the top 10 pathways from MSigDB's Compute Overlaps analysis tool for the top genes (maximum 200 genes). (A) Top 10 significant pathways for top genes more abundant in WT (*Bap1*$^{wt/wt}$;*Tyr-Cre*$^{+}$) neurons and for (B) top genes more abundant in *TyrBap1* KO neurons. For each dot plot, the y-axis shows the pathway name (left) with the database source for that pathway (gray panel on right side of dot plot; WP = WikiPathways). The x-axis shows the gene

ratio (number of genes in overlap / genes in pathway gene set). Color of the dots represents the -log(FDR) for the pathway and the size of the dot indicates the number of genes in overlap (number of genes in common between the pathway gene set and the list of DEGs).

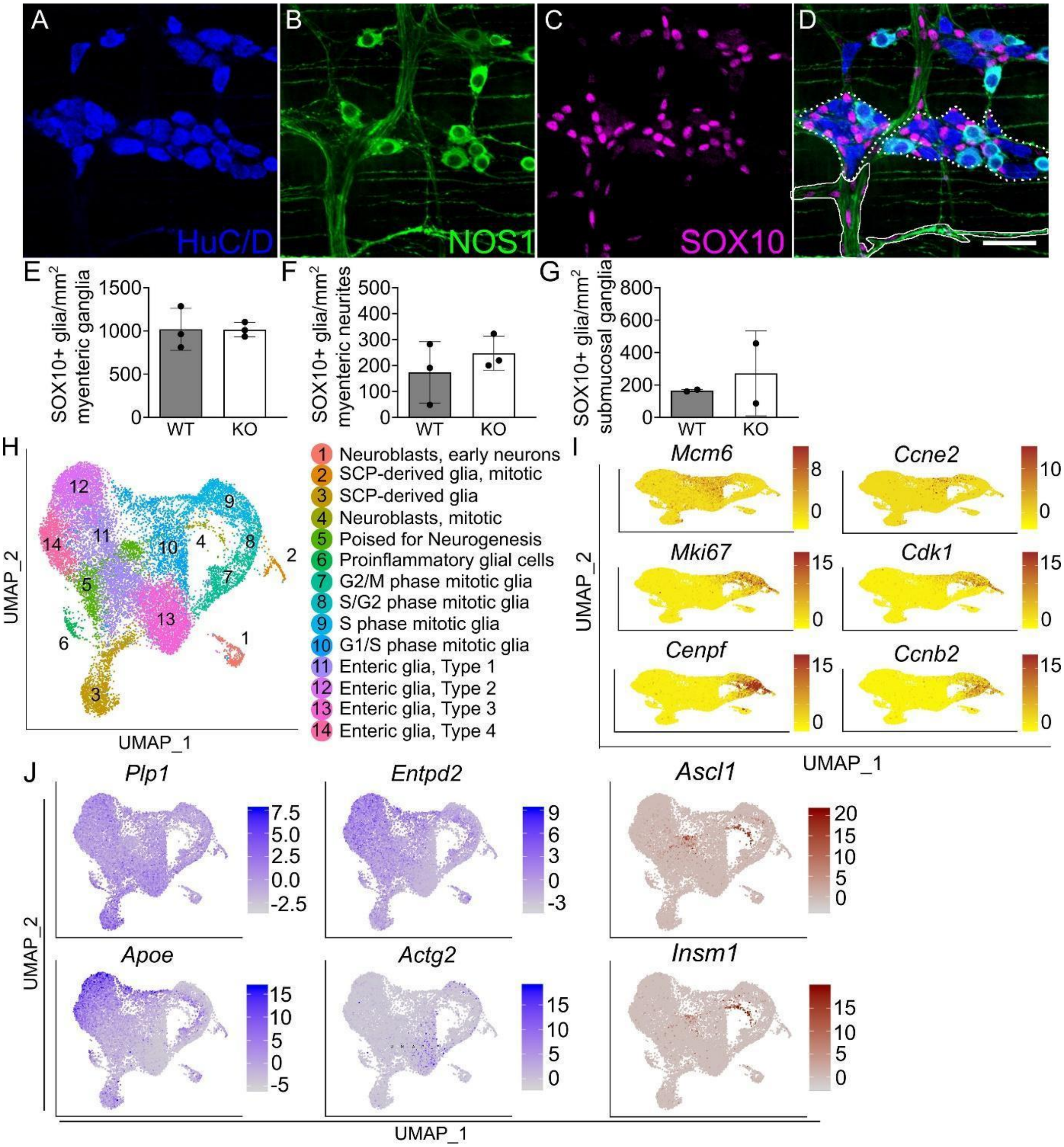

Supplemental Figure 9. Enteric glia are only mildly affected in *TyrBap1* KO mice based on cell counting and single cell RNA sequencing.

(A-D) Representative maximal intensity projection Z-stacks of P15 *TyrBap1* KO distal small intestine myenteric plexus showing (A) neurons (HuC/D = blue), (B) nitrergic neurons (NOS1 = green), and (C) enteric glia (SOX10 = magenta). (D) Corresponding merged image. Dotted white lines delineate representative areas from which ganglia-associated glial counts were derived.

Solid white line surrounds a representative area from which neurite-associated glial counts were derived. Scale bar = 50 μm. (E-G) Quantification of glial cell density in distal small intestine at P15 (E) within myenteric ganglia, (F) associated with myenteric plexus neurite bundles, and (G) within submucosal ganglia comparing WT (*Bap1*$^{wt/wt}$*;Tyr-Cre*$^{+}$) and *TyrBap1* KO mice. (H) UMAP showing enteric glial subtypes at P5 (combined data from WT and *TyrBap1* KO). (I) Relative RNA expression levels superimposed on vertically compressed UMAP shown in (H) for selected cell cycle markers. (J) Relative RNA expression levels superimposed on UMAP shown in (H) for selected enteric glial markers (*Plp1, Entpd2, Apoe*), a marker of visceral smooth muscle (*Actg2*), and markers of enteric neuroblasts (*Ascl1* and *Insm1*). (I-J) Expression level defined as ln(normalized & scaled expression level) where mean expression level for each gene across all cells in the dataset is defined as ln(1).

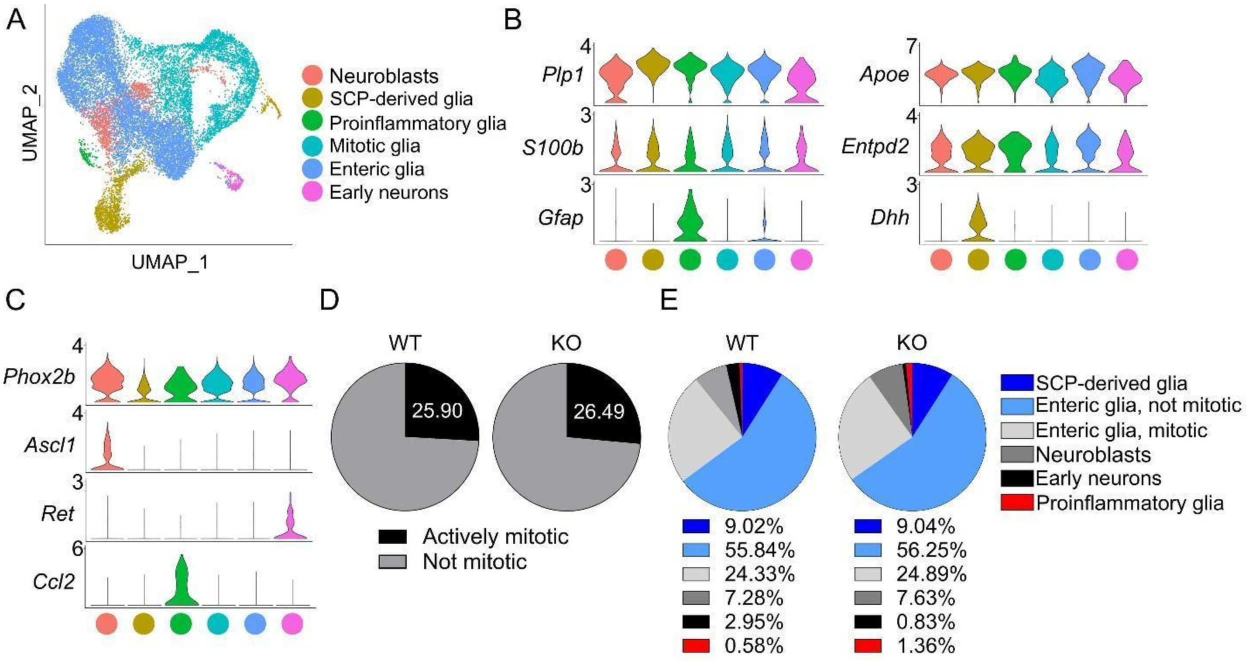

F

Mean # unique genes per cluster	Mean # unique RNA molecules per cluster	# of KO cells	# of WT cells	% of total KO cells per cluster	% of total WT cells per cluster	
2398.863	5893.837	2450	861	23.99	13.14	Enteric glia, type 1
2439.534	6121.465	1413	1242	13.83	18.96	Enteric glia, type 3
2631.309	6540.722	1064	1093	10.42	16.68	Enteric glia, type 2
2991.211	8156.109	999	528	9.78	8.06	G1/S phase mitotic glia
3223.553	9106.055	826	552	8.09	8.43	S phase mitotic glia
2801.395	7688.475	809	529	7.92	8.08	SCP-derived glia
2866.703	7702.373	819	462	8.02	7.05	Enteric glia, type 4
2539.464	6607.024	729	436	7.14	6.66	Poised for neurogenesis
2545.799	6343.06	531	374	5.20	5.71	G2/M phase mitotic glia
3447.96	10302.804	186	140	1.82	2.14	S/G2 phase mitotic glia
2648.518	6969.525	85	193	0.83	2.95	Early neurons
2932.305	8531.102	139	38	1.36	0.58	Proinflammatory glia
3615.903	11395.273	114	62	1.12	0.95	SCP-derived glia, mitotic
3622.758	11868.055	50	41	0.49	0.63	Neuroblasts, mitotic
Mean: 2907.52	Mean: 8087.57	Total: 10214	Total: 6551			

Supplemental Figure 10. Enteric glial subpopulations are only mildly affected in *TyrBap1* KO at P5 based on single cell RNA sequencing analyses.

(A) The UMAP in Supplemental Figure 9H was re-labeled. Clusters were manually combined to identify broader categories of enteric glial cell types. Colors identify each specific enteric glial subtype. (B-C) Violin plots show relative expression of (B) known markers for enteric glia (*Plp1, S100b, Gfap, Apoe, Entpd2, Phox2b*), Schwann cell precursor-derived enteric glia (*Dhh*), neuroblasts (*Phox2b*, *Ascl1*), early neurons (*Phox2b*, *Ret*), and the proinflammatory marker

(*Ccl2*). Expression level defined as ln(normalized & scaled expression level) where mean expression level for each gene across all cells in the dataset is defined as ln(1). (D) Relative abundance of glial cells actively undergoing mitosis comparing *Bap1*$^{wt/wt}$*;Tyr-Cre*$^{+}$ (WT) and *TyrBap1* KO (KO) mice. (E) Relative abundance of individual glial subtype clusters in the glial dataset comparing WT and *TyrBap1* KO mice. (F) Descriptive statistics of single cell RNA seq glial dataset derived from *TyrBap1* KO and WT tissue.

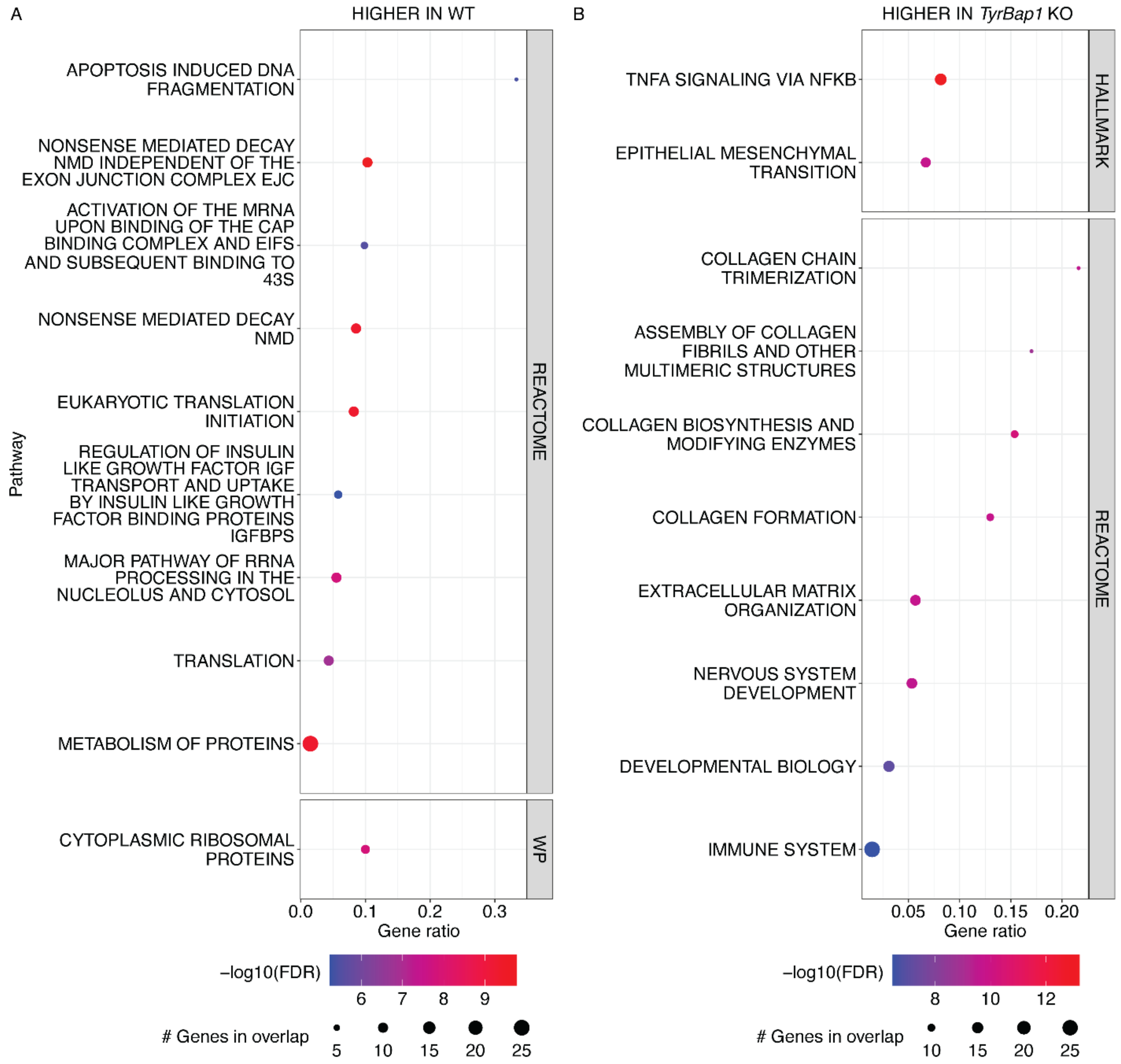

Supplemental Figure 11. Pathway analysis results of differentially expressed genes (pval<0.05) in enteric glia.

Dotplots show the top 10 pathways from MSigDB's Compute Overlaps analysis tool for the top genes in each condition (maximum 200 genes). (A) Top 10 significant pathways for top genes more abundant in WT (*Bap1*$^{wt/wt}$;*Tyr-Cre*$^{+}$) glia and (B) top genes more abundant in *TyrBap1* KO glia. For each dot plot, the y-axis shows the pathway name (left) with the database source for that pathway (gray panel on right side of dot plot; WP = WikiPathways). The x-axis shows the gene ratio (number of genes in overlap / genes in pathway gene set). Color of the dots represents the

-log(FDR) for the pathway and the size of the dot indicates the number of genes in overlap (number of genes in common between the pathway gene set and the list of DEGs).

Supplemental Table 1: ARRIVE Guidelines – General animal husbandry information

**Location of Animal facility (types of experiments)**	**CHOP (all experiments)**
Facility Type	Conventional: cages are opened in room air. Face masks are not required when handling mice
Facility Type	Specific pathogen free, Pathogens detected in room within the past 2 years: MNV (mouse norovirus), Helicobacter not tested but likely present
Bedding	¼ inch corn cob (The Andersons, Product 4B) (2018-2020);<br>Shepherd's cob blend (50:50 corncob + ALPHA-dri, Shepherd Specialty Papers) (2021-2023)
Cage type	Lab Products (Seaford, DE) 75 sq. in. Ventilated.
Cage cleaning/sterilization	Standard tunnel washer
Mouse diet	Mouse Diet 5015 (Lab Diet), direct from manufacturer.<br>Not autoclaved, not irradiated
Light/dark cycle	12 hour/12 hour
Temperature	72°F ± 2°F
Humidity	30-70% depending on the day/season
Water pH and quality	Reverse Osmosis, pH~7, Edstrom automatic watering system
Number of mice per	1-5 (20-30g)

cage	
Cage Enrichment	House/dome (Bioserve, S3174) and nestlet (Ancare)
Mating strategy	Continuous
Age at weaning	19-21 days, unless during specific experiments (survival curve, Tyr-Cre KO mice not weaned but allowed to co-house with mother until death)
Access to food and water	Continuous
Animal welfare assessment	Daily
Cage changes	1x/week (2018-2020), 1x/every 2 weeks (2020-2023)

Supplemental Table 2: List of PCR primers for mouse genotyping

Gene (Strain)	Primer Sequence	Band size	Genotyping solution	Reference
Bap1	F: 5'-AGC GTG CTT CTG AAC TGC AGC AAT GTG GAT-3'<br>R: 5'-TTC TGA AAG GGC AGT GGT GGC AAA TGA GAC-3'	Mut: 520bp<br>WT: 423bp	Taq (NEB, Cat #M0271L)	Guo *et al.* [36]
Gfp (ChAT-EGFP-L10a)	F: 5'-TCA TAG AGG CGC AGA GTT CC-3'<br>R: 5'-CTG AAC TTG TGG CCG TTT AC-3'	Mut: 250bp	KAPA (KAPA Biosystems, Cat #KK7352)	JAX genotyping protocol (Stock No: 030250)
Cre (Wnt1-Cre and *Tyr-Cre)*	F: 5'-GCA TTA CCG GTC GAT GCA ACG AGT GAT GAG-3'<br>R: 5'-GAG TGA ACG AAC CTG GTC GAA ATC AGT GCG-3'	408bp	KAPA (KAPA Biosystems, Cat #KK7352), Taq (NEB, Cat #M0271L)	https://mgc.wustl.edu/protocols/pcr_genotyping_primer_pairs
R26R-TdTomato	Common F: 5'-AAA GTC GCT CTG AGT TGT TAT-3'<br>Tg R: 5'-GCG AAG AGT TTG TCC TCA ACC-3'	Mut: ~350bp<br>WT: ~600bp	KAPA (KAPA Biosystems, Cat	JAX genotyping protocol

	WT R:<br>5'-GGA GCG GGA GAA ATG GAT ATG-3'		#KK7352), Taq (NEB, Cat #M0271L)	(Stock No: 007909)
Hdac4	F: 5'-ATC TGC CCA CCA GAG TAT GTG-3'<br>R: 5'-CTT GTT GAG AAC AAA CTC CTG CAG CT-3'	Mut: 620bp<br>WT: 480bp	Taq (NEB, Cat #M0271L)	Personal communication with Dr. Kelly A. Hyndman

Supplemental Table 3: List of antibodies

**Antibody**	**Concentration**	**Catalog number**	**Source**
Rabbit anti-NOS1	1:200	AB5380	Sigma; RRID:AB_91824
ANNA-1 (HuC/D)	N/A	N/A	Kind gift from Dr. V. Lennon, Mayo Clinic
Chicken anti-GFP	1:500	GFP-1020	Aves Labs, RRID:AB_10000240
Goat anti-SOX10	1:100	AF2864	R&D Systems, RRID:AB_442208
Rabbit anti-Cleaved Caspase-3	1:400	9661S	Cell Signaling, RRID:AB_2341188
Rabbit anti-H2AX	1:250	A300-081A	Bethyl Laboratories, RRID:AB_203288
Alexa Fluor goat anti-human 647	1:400	A21445	Thermo Fisher Scientific; RRID:AB_2535862
AlexaFluor donkey anti-rabbit 488	1:400	A21206	Thermo Fisher Scientific; RRID:AB_2535792

AlexaFluor donkey anti-rabbit 594	1:400	A21207	Thermo Fisher Scientific; RRID:AB_141637
AlexaFluor donkey anti-rabbit 647	1:400	A31573	Thermo Fisher Scientific; RRID:AB_2536183
AlexaFluor donkey anti-goat 594	1:400	A11058	Thermo Fisher Scientific; RRID:AB_2534105
AlexaFluor goat anti-chicken 488	1:400	A11039	Thermo Fisher Scientific; RRID:AB_142924
AlexaFluor donkey anti-goat 647	1:400	A21447	Thermo Fisher Scientific; RRID:AB_141844
AlexaFluor donkey anti-goat 488	1:400	A11055	Thermo Fisher Scientific; RRID:AB_2534102
AlexaFluor goat anti-rabbit 488	1:400	A31556	Thermo Fisher Scientific; RRID:AB_221605

DyLight donkey anti-human 488	1:200	ab102424	Abcam; RRID:AB_10710634
DyLight donkey anti-human 650	1:200	ab102427	Abcam; RRID:AB_10711474

Supplemental Table 4: Descriptive statistics

**Figure, Panel**	**Description**	**Genotypes (sample number, sex)**	**Statistical Test**	**Descriptive Statistics (Mean, SD - if sample normally distributed or n ≥ 3), otherwise [25%ile; Median; 75%ile]**
Figure 1A	Weights of *Tyr-Cre;Bap1* mice	KO: n=111 measurements across all ages, 6.76 ± 10.25 measurements per time	p=0.0001, Repeated Measures	Simple regression with 95%

		point || Het: n=177 measurements across all ages, 10.50 ± 15.58 measurements per time point || WT: n=168 measurements across all ages, 11.06 ± 15.49 measurements per time point<br>Sexes unknown	One-way ANOVA mixed effects model	confidence interval of mean weight per time point (KO: R^2=0.4861, Het: R^2=0.8334, WT: R^2=0.8873)
Figure 1C	Survival of *Tyr-Cre;Bap1* mice	KO: n = 37, WT: n = 40<br>Sexes unknown	p<0.0001, Log-rank (Mantel-Cox) Test	[25%ile; Median; 75%ile]<br>KO: [13.5; 20.0; 25]
Figure 1, D and H	Bowel phenotype of *Tyr-Cre;Bap1* mice at P15 and >P20	Sample size at P15: KO: n=23, Het: n=19, WT: n=15<br>Sample size for ages >P20: KO: n=9, Het: n=17, WT: n=20<br><br>Sexes unknown	N/A	N/A

Figure 1J	Percentage of *Tyr-Cre;Bap1* mice with incomplete melanocyte migration	KO: n=33, WT: n=76 Sexes unknown	p<0.0001, Two tailed binomial test	WT: 98.68% black fur, KO: 30.3% black fur
Figure 2A	Mean FITC fluorescence distribution along the length of the bowel 90 minutes after gavage	KO (n=6, sex unknown), Het (n=5, sex unknown), WT (n=4, sex unknown)	N/A	N/A
Figure 2B	Geometric center of FITC fluorescence along the length of the bowel 90 minutes after gavage	KO: n=6 (sex unknown), Het: n=5 (sex unknown), WT: n=4 (sex unknown)	p=0.0045 (KO vs Het: P=0.0014, KO vs WT: P=0.0049), Welch's ANOVA test with multiple comparisons	(mean ± SD) KO: 5.48 ± 1.21, Het: 8.47 ± 1.35, WT: 8.26 ± 0.33

Figure 2C	Number of stool pellets passed in 8 hours	KO: n=9 (4 females/3 males/2 unknown),<br>Het: n=9 (4 females/5 males),<br>WT: n=10 (6 females/4 males)	p=0.0001, Kruskal-Wallis Test with Dunn's Multiple Comparisons (KO vs Het: p=0.0016, KO vs WT: p=0.0004)	[25%ile; Median; 75%ile]<br>KO [0.00; 0.00; 0.00], Het [1.50; 4.00; 5.00], WT [2.75; 3.50; 5.50]
Figure 2D	Character of stool passed in 8 hours	KO: n=9 (4 females/3 males/2 unknown),<br>Het: n=9 (4 females/5 males),<br>WT: n=10 (6 females/4 males)	P<0.0001, Two-sided Binomial test	N/A
Figure 2E-G, Supplemental Figure 2F	Ex vivo motility analysis of P15 colons (quantification of colonic motor complexes)	KO: n=10 (4 females/ 6 males), Het: n=9 (6 females/ 3 males), WT: n=9 (6 females/ 3 males)	P=0.0003, Kruskal-Wallis Test with Multiple Comparisons (Het vs cKO: P = 0.0065, WT	(mean ± SD; CMC/min)<br>KO: 0.05 ± 0.10, Het: 0.28 ± 0.12, WT: 0.34 ± 0.10

			vs. cKO: P=0.0003)	
Figure 3B	Enteric Myenteric Neuron Density in P0 *Bap1;Wnt1-Cre* mice	WT: PSI n=3, DSI n=3, PCO n=3, DCO n=4, sexes for all mice are unknown | Het: PSI n=5 (1 male/ 1 female/ 3 unknown), DSI n=5 (1 male/ 1 female/ 3 unknown), PCO n=3 unknown, DCO n=4 (1 male/ 1 female/ 2 unknown) | KO: PSI n=6 (4 males/ 1 female/ 1 unknown), DSI n=8 (5 males/ 3 females), PCO n=5 (3 males/ 2 females), DCO n=6 (3 males/ 3 females)	PSI: p=0.2196, DSI: p=0.0506, PCO: p=0.9622, DCO: p=0.2682, Welch's ANOVA test with Dunnett's T3 multiple comparisons test	(mean ± SD; cells per mm^2) WT: PSI 2760.67 ± 539.82, DSI 6082.67 ± 867.32, PCO 7934.33 ± 1473.2, DCO 6173.00 ± 1114.35 | Het: PSI 2087.20 ± 893.70, DSI 3556.60 ± 1416.16, PCO 7764.67 ± 1192.57, DCO 5138.50 ± 435.065 | KO: PSI

				2015.83 ± 234.72, DSI 4419.53 ± 1071.62, PCO 7618.07 ± 1441.61, DCO 5797.63 ± 1150.12
Figure 3C	Enteric Submucosal Neuron Density in P0 *Bap1;Wnt1-Cre* mice	WT: PSI n=3, DSI n=3, PCO n=3, DCO n=4, sexes for all mice are unknown | Het: PSI n=5 (1 male/ 1 female/ 3 unknown), DSI n=5 (1 male/ 1 female/ 3 unknown), PCO n=3 unknown, DCO n=4 (1 male/ 1 female/ 2 unknown) | KO: PSI n=6, DSI n=7, PCO n=4, DCO n=6	PSI: p=0.0797, DSI: p=0.2603, PCO: p=0.4580, DCO: p=0.7805, Welch's ANOVA test with Dunnett's T3 multiple comparisons test	(mean ± SD; cells per mm^2) WT: PSI 680.07 ± 207.61, DSI 191.01 ± 135.98, PCO 59.05 ± 54.34, DCO 100.34 ± 147.25 | Het: PSI 358.75 ± 187.14, DSI 239.70 ± 144.41, PCO

				89.28 ± 93.28, DCO 47.98 ± 34.84 | KO: PSI 264.83 ± 122.95, DSI 105.25 ± 74.00, PCO 28.86 ± 20.30, DCO 43.16 ± 31.99
Figure 3H	Ex vivo motility analysis of P0 proximal small intestines (quantification of low frequency (L.F.) contractions)	Ctrl: n=10 (WT: 5 males/2 females and Het: 1 male/ 2 unknown) | KO: n=9 (6 females/ 1 male/ 2 unknown)	N/A	Ctrl (contractions - 5 mice, no contractions - 5 mice; WT: contractions - 3 mice, no contractions - 4 mice, Het: contractions - 2 mice, no contractions - 1 mouse) |

				KO (contractions - 4 mice, no contractions - 5 mice)
Figure 3I	Ex vivo motility analysis of P0 proximal small intestines (quantification of low frequency (L.F.) contractions)	Ctrl: n=10 (WT: 5 males/2 females and Het: 1 male/ 2 unknown) | KO: n=9 (6 females/ 1 male/ 2 unknown)	p=0.3368, Two-sided Student's t-test	(mean ± SD; L.F. contractions/ min)<br>Ctrl: 0.18 ± 0.13, KO: 0.11 ± 0.07
Figure 4B	Enteric Myenteric Neuron Density in P0 *Tyr-Cre;Bap1* mice	KO: PSI/DSI/PCO n=4 (2 males/ 2 females), DCO n=3 (1 male/ 2 females) | WT: PSI n=3 (1 male/ 2 females), DSI n=4 (1 male/ 3 females), PCO n=4 (1 male/ 3 females), DCO n=5 (1 male/ 4 females)	PSI: p=0.5505, DSI: p=0.3422, PCO: p=0.4139, DCO: p=0.5979, Welch's two-tailed t-test	(mean ± SD; cells per mm^2)<br>KO: PSI 2525 ± 530.2, DSI 4721 ± 796.9, PCO 7146 ± 3950, DCO 4461 ± 1779<br>WT: PSI

				2326 ± 274, DSI 6155 ± 2500, PCO 9501 ± 1240, DCO 5039 ± 1138
Figure 4B	Enteric Submucosal Neuron Density in P0 *Tyr-Cre;Bap1* mice	KO: PSI/DSI/DCO n=4 (2 males/ 2 females), PCO n=3 (1 male/ 2 females) | <br>WT: PSI n=3 (1 male/ 2 females), DSI n=4 (1 male/ 3 females), PCO n=4 (1 male/ 3 females), DCO n=5 (1 male/ 4 females)	PSI: p=0.6877, Welch's two-tailed t-test<br>—----------<br>DSI: p=0.1429, PCO: p>0.9999, DCO: p>0.9999, Mann-Whitney test	(mean ± SD; cells per mm^2)<br>KO: PSI 221.0 ± 192.9 | WT: PSI 348.4 ± 459.7<br>—----------<br>[25%ile; Median; 75%ile]<br>KO: DSI [0.00; 0.00; 61.80], PCO [0.00; 0.00; 0.00], DCO [0.00; 0.00; 535.4] |

				WT: DSI [0.00; 260.8; 487.3], PCO [0.00; 0.00; 74.10], DCO [0.00; 0.00; 132.7]
Figure 4D	%TdTOMATO + enteric myenteric neurons in P0 *Tyr-Cre;Bap1* mice	KO: PSI/DSI/DCO n=4 (2 males/ 2 females), PCO n=3 (1 male/ 2 females) | <br>WT: PSI n=3 (1 male/ 2 females), DSI n=4 (1 male/ 3 females), PCO n=4 (1 male/ 3 females), DCO n=5 (1 male/ 4 females)	PSI: p=0.7483, PCO: p=0.0184, DCO: p=0.8965, Welch's two-tailed t-test<br>—-----------<br>DSI: p=0.8857 Mann-Whitney Test	(mean ± SD; %)<br>KO: PSI 84.63 ± 12.22, PCO 71.83 ± 2.43, DCO 86.02 ± 10.29<br>WT: PSI 81.37 ± 12.67, PCO 81.90 ± 4.87, DCO 84.95 ± 12.77<br>------------<br>[25%ile; Median; 75%ile]

				KO: DSI [80.60; 86.45; 96.30] | WT: DSI [81.78; 95.40; 96.13]
Figure 4F	Enteric myenteric neuron density in P5 *Tyr-Cre;Bap1* mice	KO: all bowel regions n=3 (1 male/ 2 females)<br><br>WT: all bowel regions n=3-4 (3-4 females)	PSI: p=0.1735, DSI: p=0.3332, PCO: p=0.0171, DCO: p=0.1468, Welch's two-tailed t-test	(mean ± SD; cells per mm^2) KO: PSI 653.7 ± 106.8, DSI 1710 ± 257.8, PCO 3185 ± 364.2, DCO 1119 ± 408.8, WT: PSI 967.7 ± 271.9, DSI 1900 ± 127.2, PCO 4203 ± 270.2, DCO 1772 ± 475.0

Figure 4F	Enteric submucosal neuron density in P5 *Tyr-Cre;Bap1* mice	KO: all bowel regions n=3 (1 male/ 2 females)<br><br>WT: all bowel regions n=3-4 (3-4 females)	PSI: p=0.1401, DSI: p=0.0009, PCO: p=0.0884, DCO: p=0.2570, Welch's two-tailed t-test	(mean ± SD; cells per mm^2) KO: PSI 164.2 ± 19.50, DSI 184.8 ± 45.00, PCO 232.6 ± 203.5, DCO 73.94 ± 87.92, WT: PSI 391.5 ± 166.6, DSI 452.3 ± 48.24, PCO 563.5 ± 184.4, DCO 153.1 ± 16.68
Figure 4G	Enteric myenteric neuron density in P10 *Tyr-Cre;Bap1* mice	KO: n=3 all bowel regions (1 male/ 2 females) | <br>WT: PSI n=5 (1 unknown/ 1 male/ 3 females), DSI n=4 (1 male/ 3 females), PCO n=6	PSI: p=0.0207, DSI: p=0.0024, PCO: p=0.2169,	(mean ± SD; cells per mm^2) KO: PSI 379.5 ± 133.4, DSI

		(1 unknown/ 2 males/ 3 females), DCO n=4 (1 male/ 3 females)	DCO: p=0.0231, Welch's two-tailed t-test	841.8 ± 168.6, PCO 1694 ± 714.1, DCO 698.4 ± 304.4, WT: PSI 728.5 ± 122.8, DSI 1685 ± 138.5, PCO 2433 ± 725.1, DCO 1538 ± 168.6
Figure 4G	Enteric submucosal neuron density in P10 *Tyr-Cre;Bap1* mice	KO: n=3 all bowel regions (1 male/ 2 females) | <br>WT: PSI n=5 (1 unknown/ 1 male/ 3 females), DSI n=4 (1 male/ 3 females), PCO n=3 (1 male/2 females), DCO n=4 (1 male/ 3 females)	PSI: p=0.0011, DSI: p=0.0003, PCO: p=0.0140, DCO: p=0.1237, Welch's two-tailed t-test —------------ PSI: p=0.0357, Mann-	(mean ± SD; cells per mm^2) KO: PSI 114.7 ± 38.37, DSI 143.8 ± 6.00, PCO 36.53 ± 31.05, DCO 17.55 ± 10.29, WT: PSI 602.9 ± 149.6, DSI 461.7 ±

			Whitney test	35.37, PCO 624.1 ± 136.6, DCO 353.1 ± 316.1<br>--------------<br>[25%ile; Median; 75%ile]<br>KO: PSI [71.02; 142.8; 150.2], WT: PSI [484.9; 516.2; 764.1]
Figure 5A	Myenteric neuron density in P15 *Tyr-Cre;Bap1* mice	KO: PSI n=11 (6 males/1 female/4 unknown), DSI n=16 (6 males/3 females/7 unknown), PCO n=13 (5 males/2 females/6 unknown), DCO n=13 (6 males/5 females/2 unknown) | <br>WT: PSI n=6 (4 males/2 females), DSI n=12 (4	DSI: p<0.0001, Two-tailed unpaired t test with Welch's correction<br><br>—-------------<br>PSI: p=0.1215,	(mean ± SD; cells per mm^2)<br>KO: DSI 616.1 ± 204.7 | WT: DSI 965.6 ± 265.6<br>—----------<br>[25%ile; Median;

		males/ 6 females/2 unknown), PCO n=10 (4 males/5 females/1 unknown), DCO n=10 (4 males/6 females)	PCO: $p<0.0001$, DCO: $p=0.0032$, Two-tailed Mann-Whitney test	75%ile] KO: PSI [330.2; 378.7; 602.4], PCO [941.5; 1315; 1499], DCO [580.5; 739.2; 835.9] | WT: PSI [464.0; 584.7; 680.7], PCO [1936; 2083; 2301], DCO [867.9; 992.3; 1306]
Figure 5B	Submucosal neuron density in P15 *Tyr-Cre;Bap1* mice	KO: PSI n=11 (6 males/1 female/4 unknown), DSI n=15 (5 males/3 females/7 unknown), PCO n=11 (5 males/2 females/4 unknown), DCO n=13 (6	PSI: $p<0.0001$, DSI: $p<0.0001$, PCO: $p<0.0001$, Two-tailed	(mean ± SD; cells per mm^2) KO: PSI 109.2 ± 56.47, DSI 120.2 ±

		males/5 females/2 unknown) | <br>WT: PSI n=8 (5 males/3 females), DSI n=12 (4 males/ 6 females/2 unknown), PCO n=9 (4 males/5 females), DCO n=10 (4 males/6 females)	unpaired t test with Welch's correction<br><br>—------------<br>DCO: p=0.0032, Two-tailed Mann-Whitney test	63.46, PCO 62.70 ± 48.12 | WT: PSI 562.6 ± 87.15, DSI 403.4 ± 83.95, PCO 593.1 ± 158.2<br>—--------------<br>[25%ile; Median; 75%ile]<br>KO: DCO [19.10; 27.67; 56.33] | WT: DCO [122.0; 137.2; 151.7]
Figure 5C	% TDTOMATO+ myenteric neurons per total neurons in	KO: n=4 (1 male/ 3 females) | WT: n=3 (PSI: 2 males/ 1 female, DSI/PCO/DCO: 1 male/ 2 females)	PSI: p=0.0147, DSI: p=0.0173, PCO:	(mean ± SD; %)<br>KO: PSI 24.25 ± 14.32, DSI

	P15 *Tyr-Cre;Bap1* mice		p=0.0220, DCO: p=0.0330, Two-tailed unpaired t-test with Welch correction	30.00 ± 16.733, PCO 37.11 ± 14.27, DCO 19.95 ± 8.23 | WT: PSI 64.35 ± 13.45, DSI 65.20 ± 7.55, PCO 66.04 ± 8.82, DCO 63.92 ± 17.30
Figure 5C	% TDTOMATO+ submucosal neurons per total neurons in P15 *Tyr-Cre;Bap1* mice	KO: n=4 (1 male/ 3 females) | WT: n=3 (PSI: 2 males/ 1 female, DSI/PCO/DCO: 1 male/ 2 females)	PSI: p=0.0295, DSI: p=0.2288, PCO: p=0.5976, DCO: p=0.3570, Two-tailed unpaired t-test with Welch correction	(mean ± SD; %) KO: PSI 47.36 ± 14.34, DSI 67.72 ± 14.93, PCO 54.73 ± 25.92, DCO 39.51 ± 37.91 | WT: PSI 75.56 ±

				10.36, DSI 79.01 ± 3.09, PCO 62.89 ± 10.44, DCO 67.45 ± 34.22
Figure 5D	*Chat*-GFP+ myenteric neuron in P15 *Tyr-Cre;Bap1* mice	KO: PSI n=4 (3 males/1 female), DSI n=4 (2 males/2 females), PCO n=5 (3 males/2 females), DCO n=6 (3 males/3 females) | <br>WT: PSI n=4 (3 males/1 female), DSI n=4 (2 males/ 2 females), PCO n=4 (2 males/2 females), DCO n=4 (2 males/2 females)	PSI: p=0.0284, DSI: p=0.0031, PCO: p=0.0203, DCO: p=0.0117, Welch's two-tailed unpaired t-test	(mean ± SD; cells per mm^2)<br>KO: PSI 248.6 ± 69.38, DSI 333.0 ± 106.1, PCO 382.7 ± 61.83, DCO 158.9 ± 44.85 | WT: PSI 376.7 ± 53.36, DSI 683.1 ± 51.47, PCO 1369 ± 443.9, DCO 589.1 ± 163.2

Figure 5E	*Chat*-GFP+ submucosal neuron in P15 *Tyr-Cre;Bap1* mice	KO: PSI n=3 (3 males), DSI n=4 (2 males/2 females), PCO n=5 (3 males/2 females), DCO n=6 (3 males/3 females) | <br>WT: PSI n=4 (3 males/1 female), DSI n=4 (2 males/ 2 females), PCO n=4 (2 males/2 females), DCO n=4 (2 males/2 females)	PSI: p=0.0006, DSI: p=0.0150, DCO: p=0.0087 —-------------- PCO: p=0.0159, Two-tailed Mann-Whitney test	(mean ± SD; cells per mm[2]) KO: PSI 53.45 ± 31.64, DSI 64.68 ± 32.17, PCO 10.63 ± 14.04, DCO 15.33 ± 10.63 | WT: PSI 274.1 ± 27.39, DSI 240.2 ± 79.14, PCO 161.7 ± 87.48, DCO 36.52 ± 8.33 —------------ [25%ile; Median; 75%ile] KO: PCO

				[0.00; 0.00; 21.61]<br>WT: PCO [88.74; 147.0; 249.4]
Figure 5F	% *Chat*-GFP+ myenteric neurons per total neurons in P15 *Tyr-Cre;Bap1* mice	KO: PSI n=4 (3 males/1 female), DSI n=4 (2 males/2 females), PCO n=5 (3 males/2 females), DCO n=6 (3 males/3 females) | <br>WT: PSI n=4 (3 males/1 female), DSI n=4 (2 male/ 2 females), PCO n=4 (2 males/2 females), DCO n=4 (2 males/2 females)	PSI: p=0.0320, DSI: p=0.0040, PCO: p<0.0001, DCO: p<0.0001, Welch's two-tailed unpaired t-test	(mean ± SD; %)<br>KO: PSI 39.89 ± 4.70, DSI 46.50 ± 3.70, PCO 26.51 ± 2.87, DCO 22.64 ± 6.34 | WT: PSI 66.08 ± 14.67, DSI 66.70 ± 6.73, PCO 54.36 ± 3.51, DCO 51.11 ± 4.82
Figure 5F	% *Chat*-GFP+ submucosal neurons per	KO: PSI n=3 (3 males), DSI n=4 (2 males/2 females), PCO n=5 (3 males/2	PSI: p=0.2568, DSI:	(mean ± SD; %)<br>KO: PSI

	total neurons in P15 *Tyr-Cre;Bap1* mice	females), DCO n=6 (3 males/3 females) | <br>WT: PSI n=4 (3 males/1 female), DSI n=4 (2 male/ 2 females), PCO n=4 (2 males/2 females), DCO n=4 (2 males/2 females)	p=0.2796, DCO: p=0.2022, Welch's two-tailed unpaired t-test<br>—----------<br>PCO: p=0.0556, Two-tailed Mann-Whitney test	38.44 ± 7.10, DSI 43.28 ± 16.04, DCO 35.63 ± 17.31 | WT: PSI 44.89 ± 4.61, DSI 55.76 ± 13.46, DCO 28.34 ± 3.26<br>—-----------<br>[25%ile; Median; 75%ile]<br>KO: PCO [0.00; 0.00; 17.60]<br>WT: PCO [14.31; 22.22; 46.39]
Figure 5G	NOS1+ myenteric neuron density in P15 *Tyr-Cre;Bap1* mice	KO: PSI n=7 (3 males/ 4 unknown), DSI n=8 (1 male/ 1 female / 4 unknown), PCO n=5 (1 male/ 4 unknown),	PSI: p=0.0147, DSI: p=0.0154, PCO:	(mean ± SD; cells per mm^2)<br>KO: PSI 89.87 ±

		DCO n=7 (3 males/ 2 female/ 2 unknown)] | <br>WT: PSI n=3 (2 males/ 1 female), DSI n=6 (2 males/ 4 females), PCO n=5 (2 males/ 3 females), DCO n=4 (2 males/ 2 females)]	p=0.0062, DCO: p=0.4196, Welch's two-tailed unpaired t-test	26.39, DSI 170.9 ± 46.13, PCO 460.5 ± 126.1, DCO 256.6 ± 88.17;<br>WT: PSI 148.5 ± 21.18, DSI 254.4 ± 56.71, PCO 742.7 ± 115.4, DCO 287.8 ± 30.31
Figure 5H	NOS1+ submucosal neuron density in P15 *Tyr-Cre;Bap1* mice	KO: PSI n=7 (3 males/ 4 unknown), DSI n=7 (3 males/ 4 unknown), PCO n=4 (1 male/ 3 unknown), DCO n=6 (2 males/ 3 females) | <br>WT: PSI n=3 (2 males/ 1 female), DSI n=6 (2 males/ 4 females), PCO n=5 (2	PSI: p=0.1227, DSI: p=0.4766, PCO: p=0.5434, DCO: p=0.0003, Welch's two-tailed	(mean ± SD; cells per mm^2)<br>KO: PCO 19.93 ± 23.29, DCO 14.11 ± 13.25;<br>WT: PCO 31.71 ±

		males/ 3 females), DCO n=7 (3 males/ 4 females)	unpaired t-test<br>—----------<br>PSI: p=0.1083, DSI: p=0.6562, Mann-Whitney test	31.98, DCO 58.59 ± 14.89<br>—------------<br>[25%ile; Median; 75%ile]<br>KO: PSI [9.69; 23.98; 75.82], DSI [1.85; 5.54; 16.60], WT: PSI [43.35; 92.27; 94.08], DSI [3.18; 11.62; 15.22]
Figure 5I	% NOS1+ myenteric neurons per total neurons in P15 *Tyr-Cre;Bap1* mice	Same sex breakdown as for Figure 5G	PSI: p=0.3632, DSI: p=0.2358, PCO: p=0.9881, DCO: p=0.9086, Welch's two-	(mean ± SD; %)<br>KO: PSI 26.25 ± 3.42, DSI 33.87 ± 13.28, PCO 39.87 ± 6.92, DCO 36.35 ± 10.23;

			tailed unpaired t-test<br>—----------<br>DSI: p=0.2774, Two-tailed Mann-Whitney test	WT: PSI 28.93 ± 3.84, DSI 26.91 ± 5.10, PCO 39.78 ± 10.40, DCO 35.86 ± 2.35<br>—----------<br>[25%ile; Median; 75%ile]<br>KO: DSI [23.76; 33.42; 35.78], WT: DSI [22.22; 27.13; 31.91]
Figure 5I	% NOS1+ submucosal neurons per total neurons in P15 *Tyr-Cre;Bap1* mice	Same sex breakdown as for Figure 5H	PSI: p=0.2134, DSI: p=0.1773, PCO: p=0.6863, DCO: p=0.1136,	(mean ± SD; %)<br>KO: PSI 24.12 ± 14.47, DSI 14.81 ± 20.36, PCO 15.75 ±

			Welch's two-tailed unpaired t-test —-------------- DSI: p=0.1807, Mann-Whitney test	18.87, DCO 22.93 ± 19.50; WT: PSI 15.63 ± 5.35, DSI 30.00 ± 2.47, PCO 11.09 ± 12.10, DCO 40.36 ± 16.15 —---------- [25%ile; Median; 75%ile] KO: DSI [1.24; 7.63; 21.13], WT: DSI [0.07; 3.00; 4.52]
Figure 6A	Quantification of the number of cleaved Caspase-3+ cells per mm^2 at P5	KO: n=3 (1 male/ 2 females) WT: n=3 (1 male/ 2 females)	p=0.6742, Two tailed Unpaired t-test	(mean ± SD; cells per mm^2) KO: 1.317 ± 0.456, WT: 2.108 ± 2.993

Figure 6B	Proportion of cleaved Caspase-3+ cells at P5	KO: n=3 (1 male/ 2 females) WT: n=3 (1 male/ 2 females)	p=0.8432, Two tailed Unpaired t-test	(mean ± SD; %) KO: 0.1128 ± 0.000, WT: 0.1297 ± 0.000
Figure 6H	Quantification of the number of EdU+ neurons per mm^2 at P7	KO: n=3 (1 male/ 2 females) WT: n=3 (2 males/ 1 female)	p=0.8393, Two tailed Unpaired t-test	(mean ± SD; neurons per mm^2) KO: 11.07 ± 6.33, WT: 12.65 ± 10.98
Figure 6I	Proportion of EdU+ neurons per mm^2 at P7	KO: n=3 (1 male/ 2 females) WT: n=3 (2 males/ 1 female)	p=0.6057, Two tailed Unpaired t-test	(mean ± SD; %) KO: 1.018 ± 0.000, WT: 0.7802 ± 0.000
Figure 6O	Quantification of the number of γH2AX+ neurons per mm^2 at P5	KO: n=3 (2 males/ 1 female) WT: n=3 (1 male/ 2 females)	p=0.4226, Two tailed Unpaired t-test	(mean ± SD; neurons per mm^2) KO: 2.460 ± 4.260; WT: 0.000 ± 0.000

Figure 6P	Proportion of γH2AX+ neurons per mm^2 at P5	KO: n=3 (2 males/ 1 female)<br>WT: n=3 (1 male/ 2 females)	p=0.3739, Two tailed Unpaired t-test	(mean ± SD; %)<br>KO: 0.1840 ± 0.000; WT: 0.0000 ± 0.000
Figure 6V	Survival of *Tyr-Cre;Bap1;Hdac4* mice	*Bap1 fl/fl; Hdac4 wt/wt*: n=57<br>*Bap1 fl/fl; Hdac4 fl/wt*: n=13<br>*Bap1 fl/fl; Hdac4 fl/fl*: n=5<br>*Bap1 fl/wt; Hdac4 fl/fl* plus *Bap1 wt/wt; Hdac4 fl/fl*: n=5	p<0.0001, Log-rank (Mantel-Cox) Test	(median survival, days)<br>*Bap1 fl/fl; Hdac4 wt/wt*: 20 | *Bap1 fl/fl; Hdac4 fl/wt*: 23.5 | *Bap1 fl/fl; Hdac4 fl/fl*: 13 | *Bap1 fl/wt; Hdac4 fl/fl* and *Bap1 wt/wt; Hdac4 fl/fl*: Undefined

Figure 7 and Supplemental Figure 7	Single cell sequencing samples, distal colon myenteric plexus, P5	KO: n=3 males<br><br>WT: n=5 (3 males/ 2 females)	N/A	N/A
Figure 8A	Survival of *Ret-CreERT2;Bap1* mice treated with tamoxifen at >8 weeks age	KO: n=13 (8 females/ 5 males), Het: n=6 (3 females/ 3 males), WT: n=5 (4 females/1 male)	Unable to calculate Log-rank (Mantel-Cox) Test due to zero deaths in testing period	N/A
Figure 8B	Survival of *Ret-CreERT2;Bap1* mice treated with tamoxifen at age P1-P7	KO: n=9 (5 females/ 3 males/ 1 not known)<br>Het: n=7 (5 females/ 2 males)<br>WT: n=26 (11 females/ 11 males/ 4 not known)	p=0.4550, Log-rank (Mantel-Cox) Test	N/A
Figure 8C	Weights of *Ret-CreERT2;Bap1* mice treated with tamoxifen	KO: n=8 (3 females/ 5 males), Het: n=5 (2 females/ 3 males), WT: n=3 females	P=0.2807, Simple Linear Regression	(mean ± SD; grams)<br>KO: 24.49 ± 0.9826, Het:

	at >8 weeks age			25.20 ± 1.133, WT: 21.87 ± 0.7956
Figure 8D	Weights of *Ret-CreERT2;Bap1* mice treated with tamoxifen at age P1-P7	KO: n=7 (5 females/ 2 males), Het: n=5 (3 females/ 2 males), WT: n=16 (6 females/ 10 males)	p=0.2076, Repeated Measures One-way ANOVA mixed effects model with multiple comparisons	(mean ± SD; grams) KO: 16.68 ± 5.344), Het: 16.99 ± 5.210, WT: 16.09 ± 4.834
Figure 8G	Percentage of TdT+ enteric myenteric neurons in *Ret-CreERT2;Bap1* mice treated with tamoxifen in adulthood	WT: n=3 (3 males)<br>Het: n=3 (1 male/2 females)<br>KO: n=5 (3 males/2 females)	PSI MP: p=0.7134, DSI MP: p=0.1002, PCO MP: p=0.3123, DCO MP: p=0.9176, Ordinary One-Way	(mean ± SD; %)<br>WT | Het | KO<br>PSI MP: 89.78±5.041 | 88.49±9.585 | 92.79±2.361, DSI MP: 89.41±2.748 | 59.20±32.94 |

			ANOVA	86.18±4.217, PCO MP: 88.90±2.518 | 84.79±2.768 | 84.03±11.97, DCO MP: 90.92±4.789 | 91.47±5.274 | 89.67±7.280
Figure 8G	Percentage of TdT+ enteric submucosal neurons in *Ret-CreERT2;Bap1* mice treated with tamoxifen in adulthood	WT: n=3 (3 males)<br>Het: n=3 (1 male/2 females)<br>KO: n=5 (3 males/2 females)	PSI SMP: p=0.1382, DSI SMP: p=0.5945, PCO SMP: p=0.4266, DCO SMP: p=0.3753, Ordinary One-Way ANOVA	(mean ± SD; %)<br>WT | Het | KO<br>PSI SMP: 95.53±5.404 | 83.85±6.470 | 93.24±5.131, DSI SMP: 98.35±2.855 | 92.93±11.28 | 92.33±8.092, PCO SMP: 99.43±0.984 | 97.44±2.254 | 94.27±8.048, DCO SMP:

				99.59±0.713 | 100.00±0.000 | 98.34±2.272
Figure 8H	Percentage of TdT+ enteric neurons in *Ret-CreERT2;Bap1* P8-P9 pups treated with tamoxifen from P2-P7	KO: n=3 males<br>Het: n=1 male<br>WT: n=1 male (DCO only)	N/A	(mean ± SD; %)<br>PSI MP: 86.36±3.153, DSI MP: 81.84±6.974, PCO MP: 86.04±3.524, DCO MP: 88.90±2.065, PSI SMP: 90.75±3.753, DSI SMP: 89.76±1.435, PCO SMP: 94.24±1.677, DCO SMP: 98.85±0.869
Figure 8I	FITC Dextran small bowel	KO: n=6 (2 males/ 4 females)	p=0.3403, Brown-	(mean ± SD)<br>KO:

	transit assay for *Ret-CreERT2;Bap1* mice treated with tamoxifen between P1-P9, Geometric center	Het: n=3 (1 male/ 2 females)<br>WT: n=6 (3 males/ 3 females)	Forsythe ANOVA test	11.70±0.812, Het: 10.57±1.042, WT: 11.85±1.673
Figure 8I	FITC Dextran small bowel transit assay for *Ret-CreERT2;Bap1* mice treated with tamoxifen in adulthood, Geometric center	KO: n=6 (2 males/ 4 females)<br>Het: n=3 (2 males/ 1 female)<br>WT: n=4 females	p=0.0581, Brown-Forsythe ANOVA test	(mean ± SD) KO: 9.461±2.349, Het: 9.406±0.204, WT: 10.89±1.509
Figure 8J	Colon bead expulsion latency in *Ret-CreERT2;Bap1* mice treated with tamoxifen	KO: n=5 (3 males/ 2 females)<br>Het: n=5 (1 male/ 4 females)<br>Control: n=10 (3 males/ 7 females)	p=0.6497, Brown-Forsythe ANOVA test	(mean ± SD; sec) KO: 157.4 ± 52.22, Het: 146.5 ± 26.84, WT:

	between P1-P9			158.0 ± 44.25
Figure 8J	Colon bead expulsion latency in *Ret-CreERT2;Bap1* mice treated with tamoxifen in adulthood	KO: n=11 (4 males/ 7 females)<br>Het: n=5 (2 males/ 3 females)<br>Control: n=7 (1 male/ 6 females)	p=0.2937, Kruskal-Wallis test	[25%ile; Median; 75%ile] in sec<br>Adulthood = KO: [159.3; 189.0; 267.8], Het: [110.0; 144.5; 330.7], WT: [115.3; 171.3; 186.0]
Supplemental Figure 2G	*Tyr-Cre;Bap1* mouse colon width pre- and post-TTX treatment at P15	KO: 2 males/ 1 females<br>WT: 2 females/ 1 male	p=0.0255, Brown-Forsythe ANOVA test	(mean ± SD; mm)<br>KO + TTX: 1.6 ± 0.168 | KO no TTX: 1.682 ± 0.06 | Control + TTX: 1.575 ± 0.321|

				Control no TTX: 1.614 ± 0.178
Supplemental Figure 2H	*Tyr-Cre;Bap1* mouse % change in colon width pre- and post-TTX treatment at P15	KO: 2 males/ 1 females<br>WT: 2 females/ 1 male	p=0.8314, Unpaired T-test with Welch's correction	(mean ± SD; %)<br>KO: -3.017 ± 8.925, WT: -4.807 ± 10.29
Supplemental Figure 3A-D	Trichrome stain of radial cuts through full-thickness bowel tissue	Control: *Bap1 wt/wt;Tyr-Cre+* genotype (n=1 male) or *Bap1 fl/wt; Tyr-Cre+* genotype (n=2, 1 female/ 1 male)	N/A	N/A
Supplemental Figure 3E-H	Trichrome stain of radial cuts through full-thickness bowel tissue	KO: n= 2 females	N/A	N/A

Supplemental Figure 3K	Quantification of epithelial cell counts - proximal small intestine	WT: n=4 (2 females/2 males), KO: n=4 (2 females/ 1 male/ 1 unknown)	p=0.7976, Unpaired two-tailed t-test	(mean ± SD; villus length in µm) WT: 208.3 ± 55.27, KO: 222.3 ± 89.03
Supplemental Figure 3L	Quantification of epithelial cell counts - proximal small intestine	WT: n=4 (2 females/2 males), KO: n=4 (2 females/ 1 male/ 1 unknown)	p=0.7689, Unpaired two-tailed t-test	(mean ± SD; counts per villus) WT: 81.33 ± 30.15, KO: 88.36 ± 34.36
Supplemental Figure 3M	Quantification of epithelial cell counts - proximal small intestine	WT: n=4 (2 females/2 males), KO: n=4 (2 females/ 1 male/ 1 unknown)	p=0.3745, Unpaired two-tailed t-test	(mean ± SD; crypt depth in µm) WT: 40.10 ± 7.675, KO: 46.76 ± 11.56
Supplemental Figure 3N	Quantification of epithelial cell counts - proximal small intestine	WT: n=4 (2 females/2 males), KO: n=4 (2 females/ 1 male/ 1 unknown)	p=0.5818, Unpaired two-tailed t-test	(mean ± SD; counts per crypt) WT: 21.68 ± 4.656, KO: 23.90 ± 6.023

Supplemental Figure 3O	Quantification of epithelial cell counts - proximal small intestine	WT: n=4 (2 females/2 males), KO: n=4 (2 females/ 1 male/ 1 unknown)	p=0.9013, Unpaired two-tailed t-test	(mean ± SD; villus cell count per crypt cell count) WT: 3.816 ± 0.703, KO: 3.880 ± 0.691
Supplemental Figure 3T	Quantification of epithelial cell counts - distal small intestine	WT: n=4 (2 females/2 males), KO: n=4 (2 females/ 1 male/ 1 unknown)	p=0.0427, Unpaired two-tailed t-test	(mean ± SD; villus length in μm) WT: 152.7 ± 46.30, KO: 85.68 ± 24.25
Supplemental Figure 3U	Quantification of epithelial cell counts - distal small intestine	WT: n=4 (2 females/2 males), KO: n=4 (2 females/ 1 male/ 1 unknown)	p=0.1591, Unpaired two-tailed t-test	(mean ± SD; counts per villus) WT: 52.47 ± 19.97, KO: 35.15 ± 8.122
Supplemental Figure 3V	Quantification of epithelial cell counts - distal small intestine	WT: n=4 (2 females/2 males), KO: n=4 (2 females/ 1 male/ 1 unknown)	p=0.0808, Unpaired two-tailed t-test	(mean ± SD; crypt depth in μm) WT: 31.39 ±

				7.060, KO: 41.44 ± 3.244
Supplemental Figure 3W	Quantification of epithelial cell counts - distal small intestine	WT: n=4 (2 females/2 males), KO: n=4 (2 females/ 1 male/ 1 unknown)	p=0.3111, Unpaired two-tailed t-test	(mean ± SD; counts per crypt) WT: 17.60 ± 4.655, KO: 21.62 ± 5.602
Supplemental Figure 3X	Quantification of epithelial cell counts - distal small intestine	WT: n=4 (2 females/2 males), KO: n=4 (2 females/ 1 male/ 1 unknown)	p=0.0004, Unpaired two-tailed t-test	(mean ± SD; villus cell count per crypt cell count) WT: 3.158 ± 0.332, KO: 1.725 ± 0.237
Supplemental Figure 3Y	Quantification of PAS+ goblet cells - distal small intestine	WT: n=3 (2 females/ 1 male), KO: n=3 (2 females/ 1 male)	p=0.2772, Unpaired two-tailed t-test	(mean ± SD; counts per villus) WT: 7.764 ± 1.085, KO: 6.512 ± 0.523

Supplemental Figure 3Z	Quantification of PAS+ goblet cells - distal small intestine	WT: n=3 (2 females/ 1 male), KO: n=3 (2 females/ 1 male)	p=2753, Unpaired two-tailed t-test	(mean ± SD; PAS/AB villus count per epithelial cell count)<br>WT: 0.147 ± 0.047, KO: 0.201 ± 0.057
Supplemental Figure 3A'	Quantification of PAS+ goblet cells - distal small intestine	WT: n=3 (2 females/ 1 male), KO: n=3 (2 females/ 1 male)	p=0.0317, Unpaired two-tailed t-test	(mean ± SD; counts per crypt)<br>WT: 1.810 ± 0.274, KO: 5.312 ± 1.851
Supplemental Figure 3B'	Quantification of PAS+ goblet cells - distal small intestine	WT: n=3 (2 females/ 1 male), KO: n=3 (2 females/ 1 male)	p=0.0315, Unpaired two-tailed t-test	(mean ± SD; PAS/AB crypt count per epithelial cell count)<br>WT: 0.098 ± 0.017, KO: 0.238 ± 0.073

Supplemental Figure 3C’	Quantification of PAS+ goblet cells - distal small intestine	WT: n=3 (2 females/ 1 male), KO: n=3 (2 females/ 1 male)	p=0.0016, Unpaired two-tailed t-test	(mean ± SD; PAS/AB villus count per PAS/AB crypt count)<br>WT: 4.454 ± 0.525, KO: 1.621 ± 0.367
Supplemental Figure 3J’	Quantification of PAS+ goblet cells - proximal colon	WT: n=3 (2 females/ 1 male), KO: n=3 (2 females/ 1 male)	p=0.0350, Unpaired two-tailed t-test	(mean ± SD; counts per crypt)<br>WT: 12.27 ± 1.310, KO: 16.12 ± 1.677
Supplemental Figure 3K’	Quantification of PAS+ goblet cells - proximal colon	WT: n=3 (2 females/ 1 male), KO: n=3 (2 females/ 1 male)	p=0.9132, Unpaired two-tailed t-test	(mean ± SD; PAS/AB count per epithelial cell count)<br>WT: 0.485 ± 0.057, KO: 0.479 ± 0.079

Supplemental Figure 3H’	Quantification of epithelial cell counts - proximal colon	WT: n=4 (2 females/2 males), KO: n=4 (2 females/ 1 male/ 1 unknown)	p=0.0590, Unpaired two-tailed t-test	(mean ± SD; crypt depth in μm) WT: 66.27 ± 3.202, KO: 79.85 ± 11.23
Supplemental Figure 3I’	Quantification of epithelial cell counts - proximal colon	WT: n=4 (2 females/2 males), KO: n=4 (2 females/ 1 male/ 1 unknown)	p=0.0691, Unpaired two-tailed t-test	(mean ± SD; cell count per crypt) WT: 27.40 ± 4.254, KO: 34.63 ± 4.973
Supplemental Figure 3N’	Quantification of epithelial cell counts - distal colon	WT: n=4 (2 females/2 males), KO: n=4 (2 females/ 1 male/ 1 unknown)	p=0.3119, Unpaired two-tailed t-test	(mean ± SD; crypt depth in μm) WT: 94.86 ± 21.62, KO: 79.43 ± 17.72
Supplemental Figure 3O’	Quantification of epithelial cell counts - distal colon	WT: n=4 (2 females/2 males), KO: n=4 (2 females/ 1 male/ 1 unknown)	p=0.6394, Unpaired two-tailed t-test	(mean ± SD; cell count per crypt) WT: 41.05 ± 4.966, KO: 39.16 ± 5.851

Supplemental Figure 4F	*Wnt1-Cre;Bap1* mouse strain small bowel H.F. contractions pre- and post-TTX treatment at P0	Ctrl no TTX: n=10 (WT: 5 males/ 2 females | Het: 1 male/2 unknown)<br>KO no TTX: n=10 (6 females/ 1 male/ 3 unknown)<br>Ctrl +TTX: n=3 (WT: 2 males, Het: 1 unknown)<br>KO + TTX: n=5 (5 females)	p=0.0981, Brown-Forsythe ANOVA test	(mean ± SD; H.F. contractions/ min)<br>KO + TTX: 19.72 ± 8.74 | KO no TTX: 19.97 ± 7.4 | Ctrl + TTX: 13.30 ± 1.65 | Ctrl no TTX: 11.38 ± 4.62
Supplemental Figure 9E	*Tyr-Cre;Bap1* mouse strain at P15, quantification of SOX10+ cells (glia) associated with DSI myenteric ganglia	WT: 3 mice, sex unknown<br>KO: 3 mice, sex unknown	p=0.9797, Unpaired t test with Welch's correction	(mean ± SD; cells per mm^2)<br><br>WT: 1020 ± 242.3<br><br>KO: 1015 ± 83.92

Supplemental Figure 9F	Bap1; Tyr-Cre mouse strain at P15, quantification of SOX10+ cells (glia) associated with DSI myenteric plexus neurites	WT: 3 mice, sex unknown<br>KO: 3 mice, sex unknown	p=0.4104, Unpaired t test with Welch's correction	(mean ± SD; cells per mm^2)<br><br>WT: 173.9 ± 117.8<br><br>KO: 247.6 ± 65.42
Supplemental Figure 9G	Bap1; Tyr-Cre mouse strain at P15, quantification of SOX10+ cells (glia) associated with DSI submucosal ganglia	WT: 2 mice, sex unknown<br>KO: 2 mice, sex unknown	N/A	N/A

References for Supplemental Data

1. Subramanian A, Tamayo P, Mootha VK, Mukherjee S, Ebert BL, Gillette MA, et al. Gene set enrichment analysis: a knowledge-based approach for interpreting genome-wide expression profiles. *Proc Natl Acad Sci U S A.* 2005;102(43):15545-50.
2. Liberzon A, Birger C, Thorvaldsdottir H, Ghandi M, Mesirov JP, and Tamayo P. The Molecular Signatures Database (MSigDB) hallmark gene set collection. *Cell Syst.* 2015;1(6):417-25.
3. Liberzon A, Subramanian A, Pinchback R, Thorvaldsdottir H, Tamayo P, and Mesirov JP. Molecular signatures database (MSigDB) 3.0. *Bioinformatics.* 2011;27(12):1739-40.
4. Castanza AS, Recla JM, Eby D, Thorvaldsdottir H, Bult CJ, and Mesirov JP. Extending support for mouse data in the Molecular Signatures Database (MSigDB). *Nat Methods.* 2023;20(11):1619-20.
5. H W. ggplot2: Elegant Graphics for Data Analysis. https://www.google.com/url?q=https://ggplot2.tidyverse.org&sa=D&source=docs&ust=1708208612492333&usg=AOvVaw3BGm_pzOvEZeQvQr9F9o0l.
6. Korotkevich G, Sukhov V, Budin N, Shpak B, Artyomov MN, and Sergushichev A. Fast gene set enrichment analysis. *bioRxiv.* 2021:060012.
7. Dolgalev I. msigdbr: MSigDB Gene Sets for Multiple Organisms in a Tidy Data Format. R package version 7.5.1.9001. https://igordot.github.io/msigdbr/.
